# Classification and Graphical Analysis of Alzheimer’s Disease and Its Prodromal Stage Using Multimodal Features From Structural, Diffusion, and Functional Neuroimaging Data and the APOE Genotype

**DOI:** 10.3389/fnagi.2020.00238

**Published:** 2020-07-30

**Authors:** Yubraj Gupta, Ji-In Kim, Byeong Chae Kim, Goo-Rak Kwon

**Affiliations:** ^1^Department of Information and Communication Engineering, Chosun University, Gwangju, South Korea; ^2^Department of Neurology, Chonnam National University Medical School, Gwangju, South Korea

**Keywords:** Alzheimer’s disease, multimodal fusion, sMRI, FDG-PET, AV45-PET, DTI, rs-fMRI, APOE genotype

## Abstract

Graphical, voxel, and region-based analysis has become a popular approach to studying neurodegenerative disorders such as Alzheimer’s disease (AD) and its prodromal stage [mild cognitive impairment (MCI)]. These methods have been used previously for classification or discrimination of AD in subjects in a prodromal stage called stable MCI (MCIs), which does not convert to AD but remains stable over a period of time, and converting MCI (MCIc), which converts to AD, but the results reported across similar studies are often inconsistent. Furthermore, the classification accuracy for MCIs vs. MCIc is limited. In this study, we propose combining different neuroimaging modalities (sMRI, FDG-PET, AV45-PET, DTI, and rs-fMRI) with the apolipoprotein-E genotype to form a multimodal system for the discrimination of AD, and to increase the classification accuracy. Initially, we used two well-known analyses to extract features from each neuroimage for the discrimination of AD: whole-brain parcelation analysis (or region-based analysis), and voxel-wise analysis (or voxel-based morphometry). We also investigated graphical analysis (nodal and group) for all six binary classification groups (AD vs. HC, MCIs vs. MCIc, AD vs. MCIc, AD vs. MCIs, HC vs. MCIc, and HC vs. MCIs). Data for a total of 129 subjects (33 AD, 30 MCIs, 31 MCIc, and 35 HCs) for each imaging modality were obtained from the Alzheimer’s Disease Neuroimaging Initiative (ADNI) homepage. These data also include two APOE genotype data points for the subjects. Moreover, we used the 2-mm AICHA atlas with the NiftyReg registration toolbox to extract 384 brain regions from each PET (FDG and AV45) and sMRI image. For the rs-fMRI images, we used the DPARSF toolbox in MATLAB for the automatic extraction of data and the results for REHO, ALFF, and fALFF. We also used the pyClusterROI script for the automatic parcelation of each rs-fMRI image into 200 brain regions. For the DTI images, we used the FSL (Version 6.0) toolbox for the extraction of fractional anisotropy (FA) images to calculate a tract-based spatial statistic. Moreover, we used the PANDA toolbox to obtain 50 white-matter-region-parcellated FA images on the basis of the 2-mm JHU-ICBM-labeled template atlas. To integrate the different modalities and different complementary information into one form, and to optimize the classifier, we used the multiple kernel learning (MKL) framework. The obtained results indicated that our multimodal approach yields a significant improvement in accuracy over any single modality alone. The areas under the curve obtained by the proposed method were 97.78, 96.94, 95.56, 96.25, 96.67, and 96.59% for AD vs. HC, MCIs vs. MCIc, AD vs. MCIc, AD vs. MCIs, HC vs. MCIc, and HC vs. MCIs binary classification, respectively. Our proposed multimodal method improved the classification result for MCIs vs. MCIc groups compared with the unimodal classification results. Our study found that the (left/right) precentral region was present in all six binary classification groups (this region can be considered the most significant region). Furthermore, using nodal network topology, we found that FDG, AV45-PET, and rs-fMRI were the most important neuroimages, and showed many affected regions relative to other modalities. We also compared our results with recently published results.

## Introduction

Alzheimer’s disease (AD) is a neurodegenerative disorder that is characterized by chronic cortical atrophy (such as posterior cingulate atrophy and medial temporal atrophy), and by a progressive decline in cognitive function (Bishop et al., 2010; Albert, 2011). AD is typically diagnosed in people older than 65 years (Qiu and Kivipelto, 2009). As the life span of the population increases, the prevalence of AD and its costs to society are also increasing. Therefore, the detection of AD or its precursor forms, i.e., mild cognitive impairment (MCI) (Petersen, 2004) is an important aim in biomedical research for providing new therapeutics that help to slow the progression of AD. MCI is a transitional phase (which signifies an intermediate stage of functional and cognitive decline in normal aging and dementia patients) that is characterized by memory disturbance in the absence of dementia (Petersen, 2004; Angelucci et al., 2010), followed by widespread cognitive deficits in multiple domains until a disability threshold is reached. MCI is said to be prodromal AD (Petersen, 2004); subjects go on to develop an AD [this type of patient falls into an MCI-converting (MCIc) group]. Symptoms emerge, on average, within 2–3 years (Lopez et al., 2012). A prospective population-based study in the elderly showed that the conversion rate of MCIc patients to AD or to different forms of dementia is about 10–15% per year (Mitchell and Shiri-Feshki, 2009; Lopez et al., 2012; Wei et al., 2016). Despite our substantial knowledge of MCI converters, little is understood about the 47–67% (Ganguli et al., 2004; Lopez et al., 2012; Clem et al., 2017) of subjects diagnosed with MCI who neither return to normal cognition nor convert to dementia. In a study performed in a large community sample, 10 years identified as a MCI, 21% of those suspected to be at a greater risk for converting to dementia (Dubois and Albert, 2004; Jicha et al., 2006) managed to remain with a diagnosis of MCI (Ganguli et al., 2004; Clem et al., 2017). These studies suggest that certain subjects may not convert to AD, but rather remain diagnostically stable over a period of time (this type of patient falls into an MCI-stable, or MCIs group). Recent results from neuroimaging studies support the hypothesis that AD includes a disconnection syndrome (implying network-wide functional changes due to local structural changes) generated by a breakdown of the organized structure and functional connectivity (FC) of multiple brain regions, even in the early phase of MCI or before conversion to AD (Dubois and Albert, 2004; Bishop et al., 2010; Daianu et al., 2013; Clem et al., 2017; de Vos et al., 2018).

Previous studies have shown the potential of invasive and non-invasive biomarkers to predict conversion from MCI to AD dementia. For invasive markers, the APOE-ε4 genotype (for the carriers of the APOE-ε4 allele, brain alterations associated with AD may begin as early as infancy) (Liu et al., 2013; Dean et al., 2014), and amyloid-beta (Aβ) accumulation and neurofibrillary lesions are considered to be most important biomarkers for AD (Murphy and LeVine, 2010). Apolipoprotein-E (APOE) genotype polymorphism is considered to be the most common polymorphism in neurodegenerative diseases and has been consistently linked to normal cognitive decline in AD and MCI patients. APOE-ε4 is the strongest genetic risk factor, and it increases the risk for AD twofold to threefold. Furthermore, it lowers the age of AD onset (Michaelson, 2014). Recent developments in non-invasive neuroimaging techniques, including functional and structural imaging, have given rise to a variety of commonly used neuroimaging biomarkers for AD. Among the multiple neuroimaging modalities, structural magnetic resonance imaging (sMRI) has attracted significant interest due to its ready availability for mildly symptomatic patients and its high spatial resolution (Cuingnet et al., 2011; Salvatore et al., 2015; Wei et al., 2016; Long et al., 2017; Gupta et al., 2019b, c; Sun et al., 2019). sMRI can also reveal abnormalities in a wide range of brain areas, including gray matter (GM) atrophy in the medial temporal lobe and hippocampal/entorhinal cortex, which are identified as valuable AD-specific biomarkers for the discrimination or classification of AD patients (Cuingnet et al., 2011). In diffusion tensor imaging (DTI, or diffusion MRI), water diffusion in the brain is interpreted as an MR signal loss. Because neurodegenerative processes are accompanied by a loss of obstacles that restrict the motion of water molecules (Acosta-Cabronero and Nestor, 2014), DTI can reveal promising markers of microstructural white matter (WM) damage in AD and MCI patients. A connectivity-based analysis that applied graph theory to DTI data demonstrated disrupted topological properties of structural brain networks in AD, supporting the disconnection theory. Specifically, regional diffusion metrics for the limbic WM in the fornix, posterior cingulum, and parahippocampal gyrus have shown better performance than volumetric measurements of the GM in predicting MCI conversion (Sun et al., 2019). In clinical studies, fluorodeoxyglucose-positron emission tomography (FDG-PET), florbetapir-PET AV45 (amyloid protein imaging), and resting-state fMRI (rs-fMRI) are the most commonly used functional neuroimaging methods for AD diagnosis (Hojjati et al., 2018, 2017; Gupta et al., 2019a; Pan et al., 2019a, b). FDG-PET measures cerebral glucose metabolism via 18F-FDG in the brain, and helps to detect characteristic regional hypometabolism in AD patients, which reflects the neuronal dysfunctions in the brains of AD patients (Mosconi et al., 2008). In contrast, florbetapir-PET AV45 measures the accumulation of amyloid protein in AD brain homogenates and has faster *in vivo* kinetics. The use of florbetapir in amyloid imaging was recently validated in an autopsy study, and its safety profile allows its clinical application for brain imaging (Camus et al., 2012). Moreover, rs-fMRI imaging has been developed as a tool for mapping the intrinsic activity of the brain and for depicting the synchronization of interregional FC (Bi et al., 2018; de Vos et al., 2018). A recent rs-fMRI study showed that the FC pattern may be altered in some specific functional networks (default mode network) of AD and MCI patients. The authors found decreased FC between the hippocampus and several regions throughout the neocortex, i.e., reduced FC within the default mode networks and increased FC within the frontal networks (de Vos et al., 2018).

From the above studies, we observe that there is no clear evidence supporting the supremacy of any biomarker above another (CSF vs. APOE-ε4 vs. imaging) for the diagnostic estimation of AD. The choice of biomarkers mainly depends on price and availability. Nevertheless, some authors argue for the perfection of imaging above fluid biomarkers, given that imaging modalities can distinguish the different phases of the disease both anatomically and temporally (Khoury and Ghossoub, 2019; Márquez and Yassa, 2019). The above-mentioned studies used only a single modality to detect biomarkers for the detection of the conversion of MCI to AD. The proposed algorithm performance is approximately 80–90%, which is low compared with that of recently published multimodal studies (Zhang et al., 2011; Liu et al., 2014; Ritter et al., 2015; Xu et al., 2016; Gupta et al., 2019a). To date, it is true that no single imaging modality for biomarkers meets all of the diagnostic requirements set by previous studies (Hyman et al., 2012; Jack et al., 2018, 2016) (because each biomarker has their own advantage over others) and no single (whether genotype or fluid or imaging) biomarker can by itself correctly discriminate a heterogeneous disorder of AD with high accuracy, but several methods may provide complementary information, which leads to a call to develop a panel of neuroimaging biomarkers, or a combination of imaging and APOE or imaging with CSF data that merges information about the disease manner to improve diagnostic accuracy (Márquez and Yassa, 2019). Combining information (multimodal) from different types of neuroimaging (structural and functional) with genotype (APOE) or biochemical (CSF) information, as do sMRI, AV45-PET, FDG-PET, DTI, and rs-fMRI, can help to improve diagnostic performance for AD or MCI compared with single-modality methods (Zhang et al., 2011; Young et al., 2013; Schouten et al., 2016; Wei et al., 2016; Gupta et al., 2019a). Furthermore, it has been noted lately that a combination of biomarkers yields a powerful diagnostic technique for classifying the AD group with cognitively healthy subjects, with specificity and sensitivity scores reaching above 90% (Bloudek et al., 2011; Rathore et al., 2017; Khoury and Ghossoub, 2019).

Multiple studies have reported a combination of different neuroimaging modalities for investigating AD or MCI. Dai et al. (2012) used regional GM volumetric measures and functional measures (amplitude of low-frequency fluctuations, regional homogeneity, and regional FC strength) as features. They trained distinct maximum uncertainty LDA classifiers on functional and structural properties and merged the output of the classifiers by weighted voting. Zhang et al. (2011) used a multimodal method for the discrimination of healthy controls (HC) to AD patients. They used a kernel-based support vector machine (SVM) classifier for the classification, and they combined volumetric regional features with regional FDG-PET and CSF biomarkers. Young et al. (2013) proposed a method where they combined sMRI, FDG-PET, and APOE genotype data for the discrimination of AD with HC. These authors used Gaussian processes as a multimodal kernel method, and they applied an SVM classifier for the classification of MCIs vs. MCIc groups. However, their diagnostic accuracy was low. Another study proposed a system where multiple kernel learning (MKL) with the Fourier transform of the Gaussian kernels was applied to AD classification using both sMRI and rs-fMRI (Liu et al., 2014) neuroimages. Moradi et al. (2015) used GM density maps, age, and cognitive tests as features, and employed classification algorithms such as low-density separation and random forest for AD conversion discrimination. Another study proposed a novel method for the classification of AD using a multi-feature technique (regional thickness, regional correlative-calculated from thickness measures, and the APOE genotype) using an SVM classifier (Zheng et al., 2015). Schouten et al. (2016) combined regional volumetric measures, diffusion measures, and correlation measures between all brain regions calculated from functional MRI. They employed a logistic elastic net for classification. In addition, Liu et al. (2017) used independent component analysis and the COX model for the discrimination of MCIs to MCIc. In their study, they used sMRI and FDG-PET scans in combination with APOE data and some cognitive measures. Hojjati et al. (2018) combined the features extracted from sMRI (cortical thickness) and rs-fMRI (graph measures) for the detection of AD, employing SVM for the classification. It is worth noting that most of the above-presented multimodal methods used brain atrophy from a few manually extracted regions as a feature of sMRI and PET images for the detection of AD among different groups. However, using only a small number of brain regions as a feature in any imaging modality may not accurately reflect the spatiotemporal pattern of structural and physiological abnormalities as a whole (Fan et al., 2008). Furthermore, simply by increasing the number of modalities, combining modalities did not increase predictive power.

Therefore, the primary goal of this study was to combine five different imaging modalities (sMRI, AV45, FDG-PET, DTI, and rs-fMRI) with the APOE genotype to establish a multimodal system for the detection of AD. Moreover, in this study, we used three methods (that were completely different from each other) for the discrimination of AD from other groups. Moreover, we also aimed to discover which single modality of neuroimaging achieves high performance or plays a significant role in classifying all six binary classification groups (AD vs. HC, MCIs vs. MCIc, AD vs. MCIc, AD vs. MCIs, HC vs. MCIc, and HC vs. MCIs) based on these methods, and wanted to know which combined methods would perform well in the classification stage (whole-brain or voxel-wise analysis). Furthermore, we also aimed to discover the regions where these six binary groups massively differed from each other using voxel of interest (VOI) and graph methods. Whole-brain parcelation and voxel-wise methods were used to study regional and voxel differences in all six binary classification groups. We used NiftyReg (Young et al., 2013; Gupta et al., 2019a), the pyClusterROI script (Craddock et al., 2012), PANDA (Cui et al., 2013), DPRASF (Chao-Gan and Yu-Feng, 2010), and the CAT12 toolbox with the integration of SPM12 (Ashburner and Friston, 2001) for the extraction of features from the structural and functional neuroimaging data. Furthermore, graph-based analysis (John et al., 2017; Peraza et al., 2019) was performed to study the organization of (nodal and group) network connectivity using anatomical features (including GM volume, cortical thickness, and WM pathways between GM regions), and using the regional time series of the 200 brain regions included in the Craddock atlas. For this graph-based analysis, we used the BRAPH toolbox (Mijalkov et al., 2017). Later, we applied an MKL algorithm based on the EasyMKL (Aiolli and Donini, 2015; Donini et al., 2019) classifier for classification and for data fusion. This classifier works by simultaneously learning the predictor parameters and the kernel combination weights. Moreover, we applied a leave-one-out cross-validation technique that helps to find the optimal hypermeter for this MKL classifier. In this study, we also applied the radial basis function (RBF)-SVM classifier to compare its results with the results obtained from the EasyMKL. Our results showed that grouping different measurements (or complementary information) from the six different modalities exhibited much better performance for all six binary classification groups (using any combined-ROI, or combined-VOI, or a combination of all) than using both classifiers with the best individual modality.

## Materials and Methods

### Participants

The participants included in this study were enrolled via the ADNI, which was launched in 2003 as a multicenter public-private partnership, guided by Principal Investigator Michael W. Weiner, MD. The participants were enrolled from 63 locations across the United States and Canada. The primary goal of ADNI was to test whether sMRI, PET, new biological markers, and clinical and neuropsychological evaluation could be combined to measure the development of MCI and early AD. The criteria used for the inclusion of subjects were those defined in the ADNI procedure.^1^ The enrolled subjects were between 56 and 92 (inclusive) years old, had a study partner able to offer an independent assessment of functioning, and spoke either Spanish or English. All subjects were willing and able to undergo all test trials, including neuroimaging, and agreed to a longitudinal follow-up. Specific psychoactive medications were excluded. For this study, we downloaded data from all subjects for whom all five imaging modalities (sMRI, rs-fMRI, FDG-PET, AV45-PET, and DTI) with their APOE genotype were available on the ADNI homepage. A total of 129 subjects were classified as either healthy controls (HC, *n* = 35), MCIc (*n* = 31), MCIs (*n* = 30), or AD (*n* = 33), with matched sex and age ratios. The groups were classified according to the criteria set by the ADNI consortium (Petersen et al., 2010). In the HC group, participants had global clinical dementia rating (CDR) scores of 0, mini-mental state examination (MMSE) scores between 27 and 30, functional activities questionnaire (FAQ) scores between 0 and 4, and geriatric depression scale (GDS) scores between 0 and 4. In the MCIs group, the MMSE score was between 25 and 30, the FAQ score between 0 and 16, and the GDS score was between 0 and 13. In the MCIc group, the MMSE score was between 19 and 30, the FAQ score between 0 and 18, and the GDS score was between 0 and 10.

In the AD group, patients had a global CDR score of 1, an MMSE score between 14 and 24, an FAQ score between 3 and 28, and a GDS score between 0 and 7 (Morris, 1993). We did not consider MCI subjects who had been tracked for less than 18 months and did not convert within this period. Table 1 shows participant demographic information, including the mean age and the sex ratios per group. To assess statistically significant changes in the demographics and clinical features between these groups, a Student’s *t*-test was used with the significance level set to 0.05. We found no significant differences (*p*−value > 0.05) between the groups for age or sex ratio. To attain unbiased estimates of performance, the classification groups were then randomly split into two clusters in a ratio of 70:30 for the training and testing sets. The model was trained on the training set, and the performance measures of diagnostic specificity and sensitivity were carried out on a separate testing set. The splitting procedure preserved the age and sex distribution.

**TABLE 1:** Demographic and neuropsychological characteristics of the participants.

**Group**	**AD (*n* = 33)**	**MCIc (*n* = 31)**	**MCIs (*n* = 30)**	**HC (*n* = 35)**
Sex (M/F)	21/12	16/15	17/13	14/21
Age	75.65 ± 8.61	72.27 ± 7.40	72.90 ± 7.86	77.83 ± 6.17
Body weight (kg)	75.81 ± 13.60	80.71 ± 17.43	82.14 ± 15.03	75.76 ± 18.62
FAQ score	19.34 ± 6.53	6.16 ± 7.38	1.86 ± 2.99	0.13 ± 0.48
NPI-Q score	4.46 ± 4.01	2.64 ± 3.32	1.66 ± 1.61	0.33 ± 0.78
GDS score	2.37 ± 2.59	2.03 ± 2.02	1.26 ± 0.96	1.13 ± 1.79
MMSE score	19.59 ± 4.56	26.32 ± 3.85	28.03 ± 1.25	29.13 ± 1.20

*Values are numbers or means ± standard deviations. FAQ: functional activities questionnaire; NPI-Q: neuropsychiatric inventory questionnaire; GDS: geriatric depression scale; MMSE: mini-mental state examination.*

### sMRI Acquisition

We acquired 1.5-T T1-weighted MR images from the ADNI homepage. The MRI images were obtained from data centers using Philips, GE, or Siemens Medical system scanners. Because the acquisition protocol was different for each scanner, an image normalization step was carried out by ADNI. The image corrections included calibration, image geometry distortion due to gradient non-linearity (grad-warp), and reduction in the intensity non-uniformity due to waves, or residual intensity non-uniformity of the 1.5-T scans utilized on each image by ADNI. Further details about the sMRI images are available on the ADNI website.^2^ All scans had a resolution of 176×256×256 with 1mm spacing between each scan. In our study, we again pre-processed the obtained sMRI images using the FMRIB Software Library (FSL, v.6.0) (Smith, 2002) toolbox. For the anatomical sMRI images, this included the extraction of non-brain tissue from each image using the BET function. We then passed the skull-stripped images to the ANTs (Tustison et al., 2010) toolbox for N4 bias field correction to correct for inhomogeneous artifacts in each image. For co-registration to the standard Montreal Neurological Institute (MNI) 152 template (Grabner et al., 2006), we also used the FSL toolbox (Jenkinson et al., 2012).

### FDG-PET Image Acquisition

ADNI provides four different types of FDG-PET samples, which are labeled as: (1) Co-registered Dynamic; (2) Co-registered, Averaged; (3) Co-reg, Avg, Standardized image and Voxel size; and (4) Co-reg, Avg, Std Img and Vox Siz, Uniform Resolution. The type (3) baseline FDG-PET images were downloaded from the ADNI homepage.

The downloaded baseline FDG-PET samples were in DICOM format. In the first step, we converted these DICOM format images to the Nifty format using the dcm2nii (Li et al., 2016) toolbox. Later, these images were spatially normalized to the MNI 152 template using the SPM12 toolbox (integrated within MATLAB 2019b) with a standard 91×109×91 tensor dimension image grid, having a voxel size of 2×2×2mm^3^. This image grid was oriented so that the subjects’ anterior-posterior (AC-PC) axis was parallel to the AC-PC line. The above normalization step was carried out on two levels: a global affine transformation, followed by a non-rigid spatial transformation. The general affine transformation requires a 12-parameter design, whereas the non-rigid spatial transformation uses a sequence of the lowest frequency elements of the three-dimensional cosine transform. Furthermore, the intensity normalization step was performed by splitting each voxel depth-wise with the average score of the global GM, which was obtained with the help of the AAL template image. More details concerning the FDG-PET imaging can be found on the ADNI homepage.^3^ Moreover, after the completion of these pre-processing steps, we co-registered the FDG-PET images to their corresponding sMRI T1-weighted images using the SPM12 toolbox.

### AV45-PET Image Acquisition

Alzheimer’s Disease Neuroimaging Initiative provides four different types of AV45-PET samples, which are: (1) AV45 Co-registered Dynamic; (2) AV45 Co-registered, Averaged; (3) AV45 Co-reg, Avg, Standardized image and Voxel size; and (4) AV45 Co-reg, Avg, Std Img and Vox Siz, Uniform Resolution. The type (3) baseline AV45-PET images were downloaded from the ADNI homepage. For each scan, the 5-min frames (four for florbetapir, acquired 50–70 min post-injection) were co-registered to the frame (rigid-body translation/rotation, six degrees of freedom) using the NeuroStat “mcoreg” routine (followed by the ADNI organization) (Jagust et al., 2015). The downloaded baseline AV45-PET samples were in DICOM and ECAT formats. In the first step, we converted this DICOM and ECAT format images to the Nifty format using the dcm2nii (Li et al., 2016) toolbox. Later, these images were spatially normalized to the MNI 152 template using the SPM12 toolbox (integrated within MATLAB 2019b) with a standard 91×109×91 tensor dimension image grid, having a voxel size of 2×2×2mm^3^ using the same process we introduced in section “FDG-PET Image Acquisition” for the FDG-PET image. More details about AV45-PET imaging can be found on the ADNI website (see text footnote 3). Furthermore, after the completion of the above stated pre-processing steps, we co-registered the AV45-PET images to their corresponding sMRI T1-weighted images using the SPM12 toolbox.

### Resting-State Functional MR Image Acquisition

A 3.0-T Philips Medical sMRI scanner was used to acquire the fMRI images. All rs-fMRI images were obtained from the ADNI homepage. The sample for each subject consisted of 6720 DICOM images. The patients were required to relax, not to think, and to lie in the scanner during the scanning procedure. The sequence parameters were as follows: pulse sequence = GR, TR = 3000ms, TE = 30ms, flip angle = ^80°^, data matrix = 64×64, pixel spacing X, *Y* = 3.31mm and 3.31mm, slice thickness = 3.33mm, axial slices = 48, no slice gap, time points = 140. Because the signal-to-noise ratio (SNR) of the rs-fMRI images was limited, the collected data were pre-processed to reduce the impact of noise on the fMRI images. For the pre-processing of the rs-fMRI images, we used the Data Processing Assistant for Resting-state fMRI (DPARSF) (Chao-Gan and Yu-Feng, 2010) software, which can be downloaded from http://d.rnet.co/DPABI/DPABI_V2.3_170105.zip. For each subject, the entire pre-processing was divided into nine steps as follows: converting the DICOM format into the NIFTY format, removing the first ten time points, timing the slicing, head motion correction to adjust the time series of the images so that the brain was located in the same orientation in every image, normalization, smoothing by full width at half maximum (FWHM), removing the linear trend to eliminate the residual noise that systematically increases or decreases over time (Smith et al., 1999), temporal filtering to retain 0.01–0.08 Hz fluctuations, and removing covariates to eliminate physiological artifacts (Fransson, 2005), non-neuronal blood oxygen level-dependent (BOLD) fluctuations, and head motion.

### Diffusion Tensor Imaging Image Acquisition

Diffusion tensor imaging images were also downloaded from the ADNI homepage. The DTI protocol used spin-echo diffusion-weighted echo-planar imaging with a TR/TE of 12000/1046ms, a voxel size of 0.9375×0.9375×2.35mm^3^, a matrix size of 256×256, 45 slices, 30 gradient directions, and a *b*-value of 1000s/*mm*^2^. More details about DTI imaging can be found on the ADNI webpage.^4^ This ADNI protocol was chosen after conducting a detailed comparison of several different DTI protocols to optimize the SNR in a fixed scan time (Jahanshad et al., 2013; Chen et al., 2018). The pre-processing of DTI data was performed using the diffusion toolbox of FSL (Version 6.0, FMRIB, Oxford, United Kingdom). FSL pre-processing included (i) corrections for eddy currents and head motion, (ii) skull stripping, and (iii) fitting the data to the diffusion tensor model to compute maps of fractional anisotropy (FA) and mean diffusivity (MD). A single diffusion tensor was fitted at each voxel of the eddy- and EPI-corrected DWI images using the FSL toolbox. Scalar anisotropy maps were obtained from the consequential diffusion tensor eigenvalues λ_1_,λ_2_,*and*λ_3_. FA, a measure of the degree of diffusion anisotropy, was defined in the standard way as

(1) $$\mathrm{FA}=\sqrt{\frac{3}{2}}\,\frac{\sqrt{{\left(\lambda_{1}\,<\lambda >\right)}^{2}+{\left(\lambda_{2}\,<\lambda >\right)}^{2}+{\left(\lambda_{3}\,<\lambda >\right)}^{2}}}{\sqrt{\lambda_{1}^{2}+\lambda_{2}^{2}+\lambda_{3}^{2}}},\varepsilon \,\left[0,1\right]$$

(2) $$<\lambda >=\frac{\lambda_{1}+\lambda_{2}+\lambda_{3}}{3}$$

where < λ > is equal to the MD or average proportion of diffusion in all directions. The resulting images were smoothed with a Gaussian kernel of 5*mm* FWHM to improve the SNR and ensure a Gaussian distribution of the maps.

### APOE Genotype

The APOE genotype for each subject was also obtained from the ADNI homepage. The APOE genotype is known to affect the risk of developing sporadic AD in carriers. The APOE genotype of each subject was noted as a pair of numbers representing which two alleles were present in the blood. This genetic feature was a single categorical variable for each participant and could have had one of five possible values: (ε2, ε3), (ε2, ε4), (ε3, ε3), (ε3, ε4), and (ε4, ε4). The most common allele is APOE ε3, but carriers of the APOE-ε4 variant have an increased risk of developing AD, whereas the APOE ε2 variant confers some protection on carriers (Michaelson, 2014). Of the three genetic polymorphisms, APOE shows the highest correlation with MCI status and stability (Brainerd et al., 2013). Several recent studies have linked the APOE-ε4 status to a relatively late risk of preclinical progression to AD. Until recently, ε4 variants (including ε4/ε4 and ε4/ε3 combinations) were inconsistently linked to the MCI status, likely reflecting both clinical and methodological differences in status classification. In this study, the genotype data were obtained from a 10 ml blood sample taken at the time of the scan and sent immediately to the University of Pennsylvania AD Biomarker Fluid Bank Laboratory for analysis.

### Three Feature Extraction Processes

**FIGURE 1:**
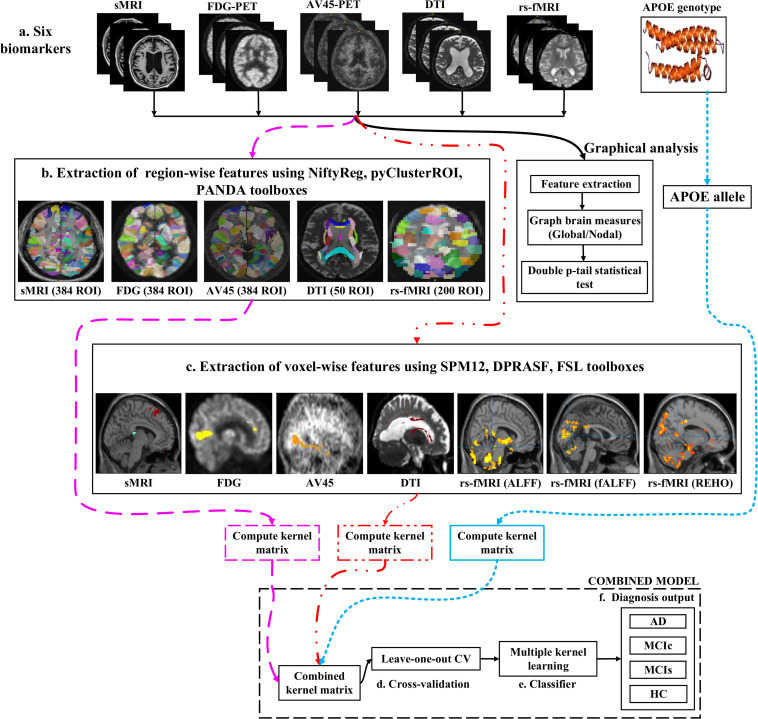
Overview of the multimodal framework. **(A)** Selection of five imaging modalities (sMRI, FDG-PET, AV45-PET, rs-fMRI, and DTI) and APOE genotype. **(B)** Extraction of regional features using NiftyReg, pyClusterROI, and PANDA toolboxes. **(C)** Extraction of voxel features using SPM12, DPARSF, and FSL toolboxes. **(D)** Leave-one-out cross-validation method. **(E)** Multiple kernel learning. **(F)** Diagnostic output.

Figure 1 shows a block diagram of the proposed framework. In this study, we performed three types of analysis for the extraction of features from each imaging modality:

(1) Whole-brain parcelation (or atlas-based segmentation) is a quantitative method that provides a non-invasive way to measure brain regions via neuroimaging. It works by assigning tissue labels to the unlabeled images using sMRI scans as well as the corresponding manual segmentation. For sMRI, FDG, and AV45-PET scans, we used the 2-mm Atlas of Intrinsic Connectivity of Homotopic Areas (AICHA) template image (Joliot et al., 2015) for the extraction of 384 regions of interest (ROIs) from each image, whereas for the rs-fMRI and DTI scans, we used the 2-mm Craddock atlas template (Craddock et al., 2012) for the extraction of 200 ROIs from each rs-fMRI image and the 2-mm Johns Hopkins University (JHU) WM labels atlas for the extraction of 50 ROIs from each DTI image (Hua et al., 2008).
(2) Voxel-wise analysis or morphometry (VBM) is a computational approach to neuroanatomy that measures differences in local concentrations of brain tissue through a voxel-wise comparison of multiple brain images. It uses statistics to recognize differences in brain structure between groups of patients, which in turn can be used to infer the presence of atrophy or normal-tissue expansion in patients with disease. For sMRI, FDG, and AV45-PET scans, we used the SPM12 toolbox to apply VBM, whereas, for the rs-fMRI scans, we used the DPRASF toolbox integrated with SPM12 to apply VBM. For the DTI scans, we used the tract-based spatial statistics (TBSS) function from FSL (v.6.0).
(3) Graph theory methods are powerful methods for quantifying the organization of a network by means of brain anatomical features, including cortical thickness, GM volume, and WM tracks between GM regions. When applying graph theory to different neuroimaging modalities, the outcome is a network with vertices or nodes that are represented by brain voxels or regions defined by a predetermined parcelation structure, while the edges are denoted by inter-individual data relations between the regions estimated, for example, as the intensity of correlation between the regional measurements. Note that the edges of a structural network are not always denoted by correlations between regional capacities, but by the density or number of WM tract-linking regions. In an analysis of sMRI, FDG, and AV45-PET networks, the nodes are generally defined using an anatomical segmentation or parcelation of the brain into different regions. In this study, we used the 2-mm AICHA atlas template image (which was already segmented into 384 distinct regions) for the extraction of 384 ROIs from each sMRI, FDG, and AV45-PET image. In the case of functional networks, for the rs-fMRI and DTI images, we used the 2-mm Craddock atlas template image for the extraction of 200 ROIs, and the 2-mm JHU-WM (ICBM-DTI-81) label atlas for the extraction of 50 ROIs from each DTI image. After the nodes of the network were defined, the edges indicating the relationship between different regions was computed. For this, we used the BRAPH toolbox (Mijalkov et al., 2017) integrated into MATLAB 2019a. In BRAPH, the edges are calculated in a GUI graphical analysis as the statistical correlation between the values of pairs of brain regions for an individual or for a group of subjects, depending on the neuroimaging technique.

#### Feature Extraction Using Atlas-Based Segmentation

After completing a series of image pre-processing steps for each imaging modality as shown in Figure 1, we extracted the features from each modality. The dashed pink line in Figure 1 shows the feature extraction for a whole-brain analysis. For sMRI, FDG, and AV45-PET images, we used the 2-*mm* AICHA atlas template image for the extraction of 384 ROIs from each image (Gupta et al., 2019a). We then processed these images using the open-source NiftyReg toolbox (Modat et al., 2010), which is a registration toolkit that performs fast diffeomorphic non-rigid registration on images. After the registration process, we obtained the subject-labeled image based on a template with 384 segmented regions. For each of the 384 ROIs in the labeled MR and PET images, we computed the GM volume and the relative cerebral metabolic rate of glucose from the baseline MRI, FDG, and AV45-PET data, respectively and later used it as a feature. Therefore, for each sMRI and PET image, we obtained 384 features. For the rs-fMRI images, we ran a pyClusterROI Python script, which was downloaded from https://ccraddock.github.io/cluster_roi/, for the extraction of 200 ROIs from each rs-fMRI image. This method employs a spatially constrained normalized-cut spectral clustering algorithm to generate individual-level and group-level parcelations. A spatial constraint was imposed to ensure that the resulting ROIs were spatially coherent, i.e., that the voxels in the resulting ROIs were connected. Moreover, for DTI images, we ran a pipeline to analyze the brain diffusion images (PANDA toolbox), which can be downloaded from https://www.nitrc.org/projects/panda/, integrated with MATLAB R2019a in the Ubuntu 18.04 operating system for the processing of diffusion MRI images. The PANDA pipeline uses the FMRIB Software Library (FSL), Pipeline System for Octave and MATLAB (PSOM), Diffusion Toolkit, and MRIcron packages for the extraction of diffusion metrics (e.g., FA and MD) that are ready for statistical analysis at the voxel level or atlas level. For the parcelation of DTI images, it uses the 2-mm JHU-WM (ICBM-DTI-81) label atlas, which is already segmented into 50 distinct ROIs. After completion of this process, we gained the subject-labeled image based on a template with 50 distinct segmented regions.

#### Feature Extraction Using Voxel-Wise Morphometry

The dashed and dotted red line in Figure 1 shows the pipeline for the voxel-wise analysis (or the extraction of features) from sMRI, FDG, AV45-PET, rs-fMRI, and DTI images. For the voxel-wise analysis of sMRI, FDG, and AV45 images, we used the statistical mapping method (SPM12) toolbox integrated with the computational anatomy toolbox (CAT version 12), which can be obtained from https://www.fil.ion.ucl.ac.uk/spm/software/spm12/ and http://www.neuro.uni-jena.de/cat/. First, the MRI data were anatomically standardized using the 12-parameter affine transformation offered by the SPM template to compensate for differences in brain size. We chose the East Asian brain image template and left all other factors at their default setting. The sMRI images were then parcellated into GM, WM, and CSF images using the unified tissue segmentation method after the image strength non-uniformity correction was complete. The obtained linearly transformed and parcellated images were then non-linearly distorted using diffeomorphic anatomical registration via exponentiated lie algebra (DARTEL) methods and modulated to create an improved template for a DARTEL-based MNI152 template image, followed by smoothing using an 8 mm FWHM kernel. The final step consisted of voxel-wise statistical assessments. To construct a statistical parametric map, we calculated contrast values based on general linear model-estimated regression parameters. This technique executes a two-sample *t*-test to determine if there are major regional density differences between two sets of GM images. We obtained these regional information values representing the major density differences between two sets of GM images after executing a false discovery rate (FDR) and family wise error rate (FWER) correction. Based on this data, cluster values were obtained to create ROI binary masks, which were subsequently used to acquire GM volumes from GM brain images for use as a morphometric feature. Moreover, before submitting FDG and AV45 images to VBM analysis, the first step was to register the FDG and AV45 images with their corresponding sMRI images. We used the SPM12 toolbox for this registration. After the registration, we followed the same method as was used for the sMRI VBM analysis. SPM12 was used to produce statistical maps of between-group alterations in the regional-to-whole-brain dimensions of the cerebral metabolic rate for glucose (CMRgl) consumption in likely AD, MCIs, MCIc, and HC groups.

**FIGURE 2:**
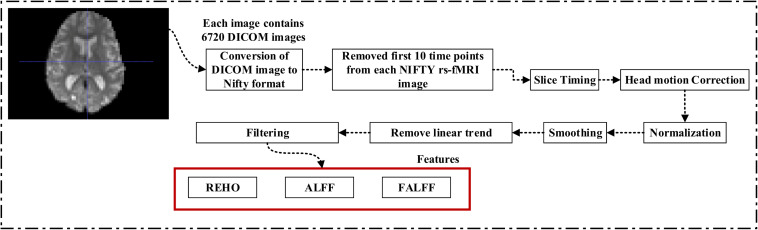
Pipeline showing feature extraction process for rs-fMRI image.

For rs-fMRI images, to decrease the influence of the SNR, the selected data should be pre-processed. In this study, we used the DPARSF toolbox,^5^ which was integrated with MATLAB R2019a, to calculate the whole-brain ALFF, fractional (fALFF), and REHO feature maps, as shown in Figure 2. Spatial smoothing was performed with a 4×4×4mm FWHM Gaussian kernel before the ALFF, FALFF, and REHO calculation. To minimize the low-frequency drift, linear trending was removed from the process. To explore the ALFF, fALFF, and REHO differences between the AD patient group and the other groups, a random-effects two-sample *t*-test was implemented on the individual ALFF, fALFF, and REHO maps in a voxel-wise way by taking the patients’ age as a confounding covariate. The ALFF was calculated by filtering the time courses of each individual voxel of the subjects with a fast Fourier transformation of the frequency area, and the power range or spectrum was then gained. Because the power of a specified frequency is relative to the square of the amplitude of this frequency, the square root was computed at each frequency domain of the power range, and then the averaged square root was found across 0.01–0.08 Hz at each individual voxel. Later, this averaged square root was taken as the ALFF index. The fALFF was calculated as the ratio of the amplitude within the low-frequency spectrum (0.01–0.08 Hz) to the total amplitude over the full frequency spectrum (0–0.25 Hz). It is generally calculated as a fraction of the sum of amplitudes across the entire frequency range detectable in a given signal. REHO is a voxel-based measure of brain activity that evaluates the similarity or synchronization between the time series of a given voxel and its nearest neighbors (Zang et al., 2004). It was calculated using Kendall’s coefficient of concordance with the time series of every 27 neighboring voxels. Then, a Kendall’s coefficient of concordance value (ranging from 0 to 1) was assigned to each voxel center. Voxels of higher strength in the REHO maps show greater similarity with the neighboring voxel’s time series. For the DTI images, FA maps were then created using the DTIfit approach and were entered into the TBSS environment to investigate changes in diffusivity measures along the WM tract. First, all FA data were non-linearly aligned to a common space (FMRIB58_FA), then the normalized FA images were averaged to create the mean FA image and a threshold set (*FA* > 0.2) (to exclude voxels that were primarily GM or CSF) to create a mean FA skeleton. Next, each participant’s FA data were projected onto the mean FA skeleton, followed by voxel-wise statistical analysis. Voxel-wise statistical analysis of FA in the WM skeleton was performed using Randomize, FSL’s non-parametric permutation interference tool. Multiple comparisons were corrected for by using threshold-free cluster enhancement (*p* < 0.05). WM regions were identified with the JHU-WM (ICBM-DTI-81) label atlas included in FSL.

#### Graph Generation and Construction of sMRI, FDG-PET, AV45-PET, fMRI, and DTI Brain Networks

To assess the sMRI, FDG-PET, and AV45-PET network topology in AD, MCIs, MCIc, and HC subjects, the T1 images of these subjects were pre-processed using the NiftyReg toolbox with the integration of the 2-mm AICHA atlas template image. In total, 384 regions were extracted from each modality and included as a node in the network study. The edges between these brain regions were computed as a Pearson correlation, and the negative correlations were set to zero. The network connectivity analyses were carried out on the binary undirected graphs while controlling the number of networks across a range of densities from 5–25% with a step size of 0.5%. To assess the functional network topology of rs-fMRI images, we used the DPARSF toolbox integrated into MATLAB 2019a. Moreover, we followed the same method for the extraction of features that we followed for the rs-fMRI images in (see section “Feature Extraction Using Voxel-Wise Morphometry”). A regional time series of the 200 brain regions included in the Craddock template atlas was extracted for each patient. To compute the relationship between these regions, we used the Pearson coefficient and performed network analysis on the binary undirected graphs. To assess the DTI network topology in AD, MCIs, MCIc, and HC subjects, the DWI images of these subjects were pre-processed using the PANDA toolbox, which was integrated into MATLAB 2019b. The PANDA toolbox uses a 2-*mm* JHU-WM (ICBM-DTI-81) label atlas template image for the extraction of 50 WM regions from each DTI image. These extracted regions were later included as a node in the network analysis. The same procedure was followed with the same parameters as that described above for constructing a network for sMRI brain images. Moreover, several graph metrics were calculated to quantify the nodal or global topological organization of the structural and functional networks, including local efficiency, characteristic path length, transitivity, and modularity. Local efficiency is a measure of the average efficiency of data transfer within local subgraphs or regions, and it is defined as the converse of the shortest average path length between the regions of a given node and all other nodes. The local efficiency of node *i* is defined as: *LE_i_* = 1/*d_i_*(*d_i_*−1)∑_*j = Gi*_1/*l*_*i,j*_, where *d_i_* represents the number of nodes in the subgraph (*G_i_*) and *l*_*i,j*_ is the length of the shortest path between nodes *i* and *j*. The distance between two vertices in a graph is the length of the shortest path between them if one exists. Otherwise, the distance is infinite, and the average of the shortest path between one node and all remaining other nodes is termed the characteristic path length. The characteristic path length *l*_*G*_ is computed as: *l*_*G*_ = 1/*n*(*n*−1)∑*_i≠j_d*(*v*_*i*_,*v*_*j*_), where *n* is the number of vertices (*v*) in a graph network, and *d*(*v*_*i*_,*v*_*j*_) denotes the shortest distance between vertices *v*_*i*_ and *v*_*j*_. Transitivity (or the clustering coefficient) is defined as the ratio of paths that cross two edges to the number of triangles. Moreover, if a node is connected to a second node, which is in turn is linked to a third node, the transitivity reproduces the probability that the initial node is linked to the third node. It can be computed by: $T=\frac{1}{N}\,\sum_{k}\frac{\sum_{i,j}a_{i\,j}\,a_{i\,k}\,a_{j\,k}}{d_{k}\,\left(d_{k}-1\right)}$, where *a*_*ij*_ is the (*i*,*j*) entry of the binary connection matrix. *a*_*ij*_ = *a*_*ji*_ = 1 if there is a link between nodes *i* and *j*, and *a*_*ij*_ = *a*_*ji*_ = 0 otherwise. There are no self-loops in the network, thus *a*_*ij*_ = 0. Modularity is the fraction of the network edges that fall within the given groups, minus the expected fraction if edges were distributed at random. It also calculates to what extent a network can be divided into communities. It can be calculated by, Q=∑i⁢ϵ⁢M[q-i⁢i(∑j⁢ϵ⁢Mq)j⁢j2], where the network is fully partitioned into *M* non-overlapping modules (or clusters), and *q*_*ij*_ represents the proportion of all links connecting nodes in module *i* with those in module *j*.

### Classification Techniques

In supervised learning, classification resembles the task of determining to which category a new sample belongs, based on a training set of data containing instances for which associations have previously been identified. In neuroimaging, different information sources may contain different imaging modalities (e.g., sMRI, FDG, AV45, DTI, and rs-fMRI), different ways of extracting features from the same modality (e.g., ROI-based or voxel-based for every image), or a different feature subset. In the present study, we applied three different methods to extract features from the same neuroimaging modality as follows: whole-brain parcelation, voxel-wise analysis, and graphical representation. We were mainly interested in the first two approaches, where we used feature subsets as a kernel for each of the two methods. Later, we will combine (or concatenate) the approaches. We were particularly interested in examining models based on subsets of features extracted according to voxels or an anatomical criterion to attain predictions that enabled us to estimate the anatomical localization.

#### Multiple Kernel Learning

**FIGURE 3:**
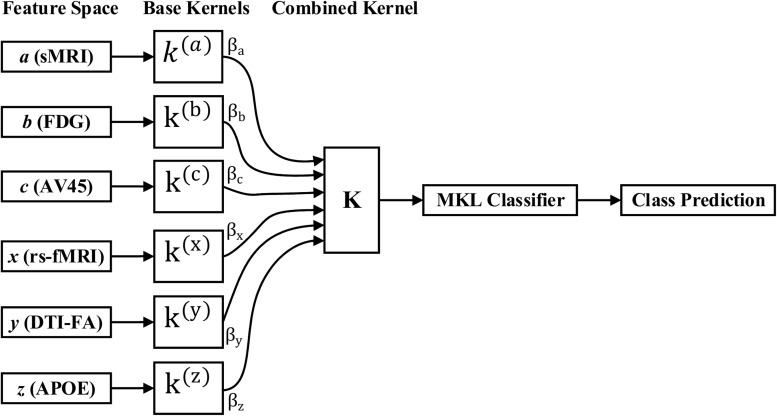
MKL classification process.

Kernel methods such as SVM (Cortes and Vapnik, 1995; Samper-González et al., 2018), which are based on similarity measures between data points, have been used with great success for dimensionality reduction and classification. Kernelization projects the native data space onto a higher-dimensional feature space. Non-linear relations between variables in the original space become linear in the transformed space. Let ${\left\{x^{\left(i\right)},y^{\left(i\right)}\right\}}_{i=1}^{N}$ be the training sample, where $x^{\left(i\right)}={\left(x_{1}^{\left(i\right)},x_{2}^{\left(i\right)},\cdots ,x_{M}^{\left(i\right)}\right)}^{T}\,\varepsilon \,{ℜ}^{M}$ is a data sample, *M* is the number of features from all modalities, and *y*^(*i*)^ε{1,−1} is the corresponding class label. The aim is to simultaneously acquire an optimal feature description and a max-margin classifier in kernel space because of its systematic and elegant way of forming complicated patterns. Therefore, to project the data, we will use the kernel trick. As we know, for any kernel (*K*) on an input space (X), there exists a Hilbert space (*f*), called the feature space, and the projection ∅ is given by the mapping ϕ:*X*→*f*, such that for any two-object (*x*,*y*) in *X*, *K* = (*x*,*y*) = < ϕ(*x*),ϕ(*y*) >, where <.,. > is the Euclidean or inner dot product of the data point. Examples of kernel functions include the linear, RBF, and others. Recent papers have shown that using multiple kernels rather than using a single kernel can improve the interpretability of a decision function, and in some instances, it improves the final performance. In MKL, the data are represented as a combination of base kernels. Each base kernel represents a different modality or feature of the entity. MKL seeks to find the optimal combination of base kernels so that the analysis tasks that follow are benefited the most. Classification tasks are represented especially well through MKL, as the optimal combination is the one that gives the maximum classification accuracy. The dual form of MKL optimization, as it is solved by conventional solvers like LIBSVM, is given as

(3) $$\begin{array}{c} \max_{\alpha }L\,\left(\alpha \right)=\sum_{i=1}^{n}\alpha_{i}-1\,/\,2\,\sum_{i,j}^{p}\sum_{m=1}^{p}\alpha_{i}\,\alpha_{j}\,y_{i}\,y_{j}\,\beta_{m}\,k^{m}\,\left(x_{i}^{m},x_{j}^{m}\right)\\ \text{Subject}\text{to}\sum_{i=1}^{n}\alpha_{i}y_{i}=0;0\leq \alpha_{i}\leq C;i=1,\ldots .,n \end{array}$$

where α_*i*_,α_*j*_ are Lagrange multipliers, which are the variables obtained on converting the primal support vectors to the dual problem, and $k^{m}\,\left(x_{i}^{m},x_{j}^{m}\right)$ is the *m-*th kernel function, which is applied to each pair of the samples, and *C* is the regularization parameter that controls the distance between the hyperplane and the support vectors. From the set of *n* training samples, the features of the *i-*th sample from the *m*-th modality are in the vector $x_{i}^{m}$, and its corresponding class label, *y*_*i*_, is either +1 or −1. The weight on the *m-*th modality kernel, represented as β_*m*_, is optimized using a grid search, or as a separate optimization problem with fixed α. For each new test sample, *s*, the kernel functions are computed against the training samples. An MKL overview is depicted in Figure 3. Recent research has shown that including the base datasets in more than one kernel, each differing in their selection of kernel parameters, improves performance. All kernels are then normalized to the unit trace through the formula $K_{m}\,\left(x,y\right)=k_{m}\,\left(x,y\right)/\sqrt{k_{m}\,\left(x,x\right)\,k_{m}\,\left(y,y\right)}$. Here, we set the weights accordingly to conform with sMRI, FDG, AV45, DTI, rs-fMRI, and APOE genotype features. The combined kernels can be described as follows,

(4) $$K\,\left(x,y\right)=w_{s\,m\,r\,i}\,k_{s\,m\,r\,i}\,\left(x,y\right)+w_{f\,d\,g}\,k_{f\,d\,g}\,\left(x,y\right)+w_{a\,v\,45}\,k_{a\,v\,45}\,\left(x,y\right)+w_{d\,t\,i}\,k_{d\,t\,i}\,\left(x,y\right)+w_{r\,s-f\,m\,r\,i}\,k_{r\,s-f\,m\,r\,i}\,\left(x,y\right)+w_{a\,p\,o\,e}\,k_{a\,p\,o\,e}\,\left(x,y\right)\,\text{With},w_{s\,m\,r\,i}+w_{f\,d\,g}+w_{a\,v\,45}+w_{d\,t\,i}+w_{r\,s-f\,m\,r\,i}+w_{a\,p\,o\,e}=1$$

Then, we use the EasyMKL (Aiolli and Donini, 2015; Donini et al., 2019) solver to search for the combination of basic kernels that maximizes the classifier performances by optimizing a simple quadratic problem addressed by SVM by computing an optimal weighting. Besides its proven empirical success, a clear advantage of EasyMKL compared with other MKL approaches is its high scalability with respect to the number of kernels to be combined. It finds the coefficients η that maximize the edges in the training dataset, where the margin is calculated as the distance between the convex hull of the positive and negative samples. In particular, the general problem that EasyMKL aims to optimize is the following,

(5) $$\left(\eta^{*},\gamma^{*}\right)=\arg\,\max_{\eta :{∥\eta ∥}_{2}=1}\min_{\gamma \,\varepsilon \,\Gamma }\lambda^{\Gamma }\,Y\,\left(\sum_{r=0}^{R}\eta_{r}\,K_{r}\right)\,Y_{\gamma }+\lambda \,{∥\gamma ∥}_{2}^{2}$$

where *y* is a diagonal matrix with training samples on the diagonal, and λ is a regularization hyperparameter, whereas domain Γ signifies two probability distributions over the set of negative and positive samples in the training set, that is $\Gamma =\left\{\gamma \varepsilon R_{+}^{l}|\sum_{y_{i}=+1}\gamma_{i}=1,\sum_{y_{i}=-1}\gamma_{i}=1|\right\}$ (Aiolli and Donini, 2015; Donini et al., 2019). Note that any element γεΓ resembles a pair of samples in the convex hull of the positive and negative training samples. At the solution, the first expression of an objective function denotes the obtained (squared) edge, which is the (squared) distance between a point in a convex hull of positive samples and a point in a convex hull of negative samples, in the considered feature space. It enforces sparsity across modalities while allowing more than one discriminative kernel to be chosen from the same modality. In other words, there is sparsity across modalities and non-sparsity within modalities, thereby making it a convex optimization problem. Moreover, in addition to the EasyMKL classifier, we also applied a RBF kernel (or Gaussian kernel) with SVM classifier for the comparison of our obtained results. An RBF kernel is a function with a score that depends on the distance from the origin (or from some points). It is represented by *K*(*x*_1_,*x*_2_) = *e*(∥*x*_1_−*x*_2_∥2/2σ^2^), where ∥*x*_1_−*x*_2_∥^2^ is the squared Euclidean distance between two data points *x*_*1*_ and *x*_*2*_. An RBF kernel has two parameters: gamma (γ) and *C*, and its performance depends on them. When the *C* value is small, the classifier is fine with misclassified input points (high bias, low variance), but when the *C* value is high, the classifier is severely penalized for misclassified data, and hence it leans over backward to avoid any misclassified input points (low bias, high variance). Moreover, when the γ value is low, the curve of the decision margin is quite low, and therefore the decision area is very large, but when the γ value is high, the curve of the decision margin is high, which creates decision-boundary bars around the input points. In our case, we applied the GridSearch method from a scikit-learn (v0.20) (Pedregosa et al., 2011) library to find the optimal hyperparameter (*C* and γ) value for the RBF-SVM classifier. The GridSearch was performed over the ranges of *C* = 1 to 9 and γ = 1^e−4^ to 7.

#### Cross Validation

Cross validation (CV) is one of the most widely utilized data resampling methods for estimating the generalization knowledge of a predictive design and for preventing under- or overfitting. CV is largely applied in settings where the aim is prediction and it is necessary to evaluate the accuracy of a predictive model. For a classification problem, a model is generally fitted with a known sample, called the training sample, and a set of unknown samples against which the model is examined, known as the test sample. The aim is to have a sample for testing the proposed model in the training period, and then present insight into how the particular model adapts to an independent sample. A round of CV involves the partitioning of samples into complementary subsets, then conducting analysis on an individual subset. After this, the study is verified on other subsets (called testing samples). To reduce variability, multiple rounds of CV are performed using several different partitions, and later an average of the outcomes is taken. CV is a powerful procedure in the evaluation of model performance. In this study, we utilized leave-one-out CV (LOOCV) from the scikit-learn (v0.20) (Pedregosa et al., 2011) library. LOOCV is a CV process in which the bulk of the fold is “1,” with “k” being fixed to the number of attributes in the dataset. This means that the number of folds equals the number of instances in the sample. Thus, the learning algorithm is employed once for each instance, utilizing all other instances as a training sample, and utilizing the selected instance as a single-item test sample. This type of CV is useful when the training samples are of limited size and the number of attributes to be verified is not high.

#### Implementation

Our classification framework and validation experiments were implemented in Python 3.5 using an interface to the scikit-learn v0.20 (Pedregosa et al., 2011) library to measure performance, and using MKLpy (v0.5)^6^ for the MKL framework. The main source code will be made available on the GitHub website.^7^ The dataset list will be available in the Supplementary Material. Nonetheless, please note that you must prepare the original image features independently.

## Results

In this section, we present the performance results of each classification (AD vs. HC, MCIs vs. MCIc, AD vs. MCI, AD vs. MCIc, HC vs. MCIs, and HC vs. MCIc) for all five neuroimaging and APOE genotype modalities using whole-brain parcelation, the voxel-wise method, and the graphical method. We implemented the multimodal fusion approaches for the integration of the sMRI, FDG, AV45, rs-fMRI, DTI, and APOE genotype data. We used the combined representation to distinguish patients with AD from healthy subjects. The combined approach is considered successful if the classification task is performed with greater accuracy and higher AUC score, higher precision, and better sensitivity and specificity against unimodal classification. Along with the unimodal approaches, we evaluated the classification of a concatenated data vector comprising data from the five neuroimaging modalities and two APOE genotype modalities, and used it as a baseline. Moreover, after the completion of extracting features from each modality using whole-brain parcelation and voxel-wise analysis, we passed these extracted features through a polynomial kernel function to map the original non-linear low-dimensional features onto a higher-dimensional space in which they became separable. After that, a data fusion technique was used to combine the multiple kernel features into a single form before passing them through the EasyMKL classifier for the binary classification of the six groups. For an unbiased performance assessment, the classification groups were randomly split into two sets at a 70:30 ratio as training and testing sets, respectively. In a training set, finding the right values for the lambda (λ) parameter is quite difficult, and their values influence the classification result. Therefore, to find the optimal hyperparameter values for a lambda from 0 to 1 of an EasyMKL algorithm, we used the leave-one-out cross-validation technique on the training set. For each method, the optimized values obtained for the hyperparameter were then used to train the EasyMKL classifier using the training group. The performance of the resulting classifier was then estimated on the remaining 30% of the data in the testing dataset, which was not used during the training step. Here, cross validation was used to assess how the result of the classification analyses could generalize to an independent group. One round of cross validation includes partitioning the data sample into disjoint subsets of instances, performing the analysis on one subsection (the training group), and validating the study on the other subset (the testing or validation set). To reduce variability, numerous rounds of cross validation are done using different subsets, and the validation outcomes are averaged over the rounds. In the present study, we used an LOOCV, which involves separating a single instance (either control or patient) from the complete example for testing while the remaining instances are used for training purposes. This splitting is iterated so that each instance in the whole sample is used once for validation. After all iterations, the final accuracy is quantified as the mean of the accuracies gained across each fold. In this way, we attained unbiased estimations of the performance for each classification problem. Moreover, after the completion of whole-brain and voxel-wise analysis, we used the BRAPH toolbox to perform the brain graphical analysis for each classification group with the same five neuroimaging features (sMRI, FDG, AV45, rs-fMRI, and DTI), which we extracted in the whole-brain analysis. For each analysis, we measured the accuracy (ACC, calculated as the average of the proportion of correctly classified subjects from each class individually), the sensitivity (SEN, described as true positive – the number of subjects correctly classified), and the specificity (SPEC, described as true negative – the number of healthy controls correctly categorized), precision (PRE, referring to how well the measurements agree with each other across multiple tests), F1-score (explained as a weighted average of recall and precision, where an F1-score attains its best value at one and its worst at zero), and AUC-ROC [a receiver operating characteristic curve (ROC curve) is a graphical plot that illustrates the diagnostic aptitude of a binary classifier scheme as its differential threshold is varied]. An AUC-ROC curve is constructed by plotting the true positive rate (TPR) against the false positive rate (FPR). The TPR is the proportion of observations that were correctly predicted to be positive out of all positive observations [*TP*/(*TP* + *FN*)]. Likewise, the FPR is the proportion of observations that are incorrectly predicted to be positive out of all negative observations [*FP*/(*TN* + *FP*)]. The ROC curve shows the trade-off between sensitivity (or TPR) and specificity (1−*FPR*). Classifiers that give curves closer to the top-left corner show better performance. As a baseline, a random classifier is expected to give points lying along the diagonal (FPR = TPR). The closer the curve comes to the 45-degree diagonal of the ROC space, the less accurate the testing data are. For each classification group, we also measured Cohen’s kappa values, which measures the inter-rater reliability between two individuals (Cohen, 1960). Kappa measures the percentage of information scores in the main diagonal of a table and then adjusts these scores for the quantity of agreement that could be assumed due to chance alone. The formula for calculating Cohen’s kappa for two raters is given by *K* = *p*_0_−*p*_*c*_/1−*p*_*c*_, where *p*_*0*_ is the relative observed agreement among raters and *p*_*c*_ is the hypothetical probability of chance agreement. Kappa is always less than or equal to 1. A value of 1 suggests perfect agreement, and scores less than 1 suggest less than the best agreement. In rare circumstances, Kappa can achieve a negative score. This signifies that the two groups agreed less than would be predicted by chance alone.

### Classification Performance Across Single and Combined Modalities Using Whole-Brain Parcelation Analysis

**FIGURE 4:**
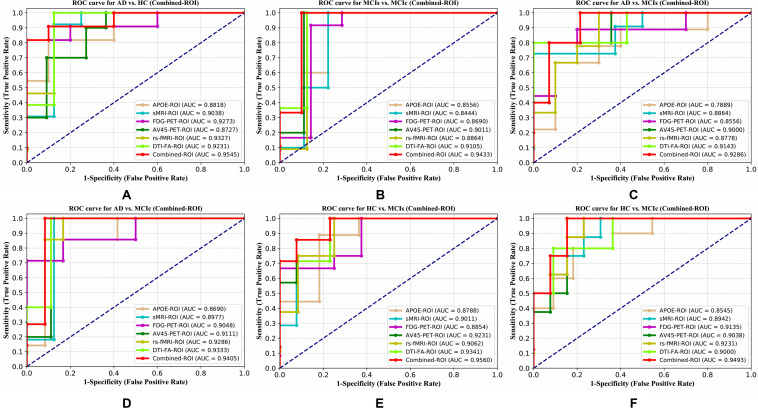
ROC curve for **(A)** AD vs. HC, **(B)** MCIs vs. MCIc, **(C)** AD vs. MCIs, **(D)** AD vs. MCIc, **(E)** HC vs. MCIs, and **(F)** HC vs. MCIc using whole-brain parcelation analysis. The red solid line shows the result of a combined-ROI curve with single modality features.

**FIGURE 5:**
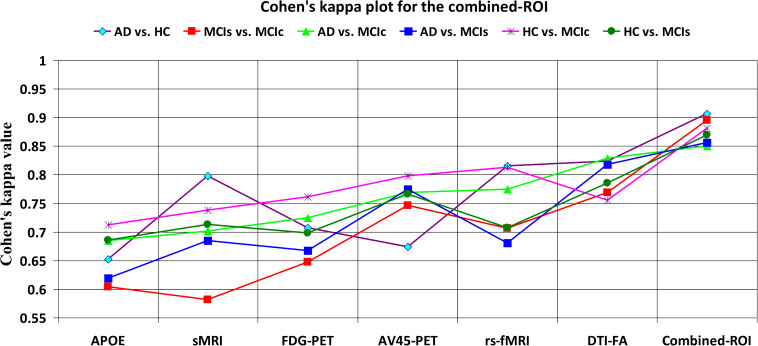
Cohen’s kappa plot for AD vs. HC, MCIs vs. MCIc, AD vs. MCIs, AD vs. MCIc, HC vs. MCIs, and HC vs. MCIc are grouped using whole-brain parcellation analysis. The above graph clearly shows the benefit of the combined-ROI modality over any single modality.

For the whole-brain analysis, we used the 2-mm AICHA atlas template image for sMRI, FDG, and AV45-PET images with the NiftyReg toolbox for the extraction of 384 ROIs from each neuroimaging modality (as shown in Figure 1B). For each rs-fMRI and DTI image, we used the 2-mm Craddock atlas template and the 2-mm JHU-WM (ICBM-DTI-81) label atlas for the extraction of 200 and 50 ROIs, from each rs-fMRI and DTI image, respectively, using the pyClusterROI Python script and the PANDA toolbox (as shown in Figure 1B). In total, we obtained 1404 features for a single image, 384 features from each sMRI, FDG, and AV45-PET image, 200 from each rs-fMRI image, 50 features from each DTI image, and two features from the APOE genotype data. Afterward, we passed these obtained features through a normalization technique to minimize the redundancy within the dataset. Furthermore, we passed these low-dimensional, normalized features from the polynomial kernel matrix to map them onto a high-dimensional feature space. Then we fused all these high-dimensional features in one form before passing them through the EasyMKL algorithm for classification. The obtained AUC-ROC graph and Cohen’s kappa scores are plotted in Figures 4, 5.

For the single modalities, whole-brain MKL analysis for AD vs. HC (Table 2), using only the APOE genotype, achieved 85.71% accuracy. Similar accuracy was obtained using sMRI (90.48%), FDG-PET (91.5%), AV45-PET (89.39%), and rs-fMRI (92.42%). Using DTI-FA, the accuracy was increased to 93.17% compared with both genotype and functional imaging. When the combined-ROI features were passed through the classifier, the accuracy increased to 96.05%. Additionally, the obtained Cohen’s kappa value was 0.9066, which is closer to 1 than the kappa values of the individual modalities. Figure 5 shows the Cohen’s kappa plot for the combined-ROI.

**TABLE 2:** Classification results for AD vs. HC, MCIs vs. MCIc, AD vs. MCIs, AD vs. MCIc, HC vs. MCIc, and HC vs. MCIs groups using ROI features (EasyMKL).

**Groups**	**Features**	**Classifier**	**Performance measure**						
			**AUC**	**ACC**	**SEN**	**SPEC**	**PRE**	**F1-score**	**Cohen’s kappa**
AD vs. HC	APOE	EasyMKL	88.18	85.71	88.89	83.33	80	84.21	0.6523
	sMRI		90.38	90.48	87.5	92.31	87.5	87.5	0.7981
	FDG-PET		92.73	91.5	**100**	84.62	80	88.89	0.7073
	AV45-PET		87.27	89.39	87.87	90.90	90.62	89.23	0.6736
	rs-fMRI		93.27	92.42	88.23	94.11	93.75	90.90	0.8150
	DTI-FA		92.31	93.17	90.90	93.93	93.75	92.30	0.8233
	**Combined-ROI**		**95.45**	**96.05**	94.11	**100**	**100**	**96.96**	**0.9066**
MCIs vs. MCIc	APOE	EasyMKL	85.56	85.24	84.37	86.20	87.09	85.71	0.6042
	sMRI		84.44	84.71	75	**100**	**100**	85.71	0.5819
	FDG-PET		86.9	86.88	87.09	86.66	87.09	87.09	0.6471
	AV45-PET		90.11	89.47	100	85.71	77.78	87.5	0.7465
	rs-fMRI		88.60	88.52	87.5	89.65	90.32	88.88	0.7060
	DTI-FA		91.05	91.80	90.62	93.10	93.54	92.06	0.7684
	**Combined-ROI**		**94.33**	**94.74**	**100**	90.91	90	**94.74**	**0.8950**
AD vs. MCIs	APOE	EasyMKL	78.89	77.68	72.73	75	80	76.19	0.6193
	sMRI		88.64	88.52	90	87.09	87.09	88.52	0.6849
	FDG-PET		85.56	84.95	83.62	85.71	84.89	84.25	0.6673
	AV45-PET		90	90.16	93.10	87.50	87.09	90	0.7746
	rs-fMRI		87.78	88.57	86.45	87.78	89.55	87.97	0.6802
	DTI-FA		91.43	91.93	90.90	93.10	93.75	92.30	0.8180
	**Combined-ROI**		**93.86**	**93.59**	**93.75**	**93.33**	**93.75**	**93.75**	**0.8562**
AD vs. MCIc	APOE	EasyMKL	86.9	85.21	80	88.89	88.89	84.21	0.6851
	sMRI		89.77	88.88	93.10	85.29	84.37	88.52	0.7014
	FDG-PET		90.48	90.47	93.33	87.87	87.5	90.32	0.7244
	AV45-PET		91.11	89.47	88.89	90	88.89	88.89	0.7689
	rs-fMRI		92.86	92.06	90.90	93.33	93.75	92.30	0.7745
	DTI-FA		93.33	93.65	93.75	93.54	**93.75**	93.75	0.8285
	**Combined-ROI**		**94.05**	**94.89**	**100**	**94.77**	91.67	**95.65**	**0.8502**
HC vs. MCIc	APOE	EasyMKL	85.45	85.71	83.33	88.89	90.91	86.96	0.7123
	sMRI		89.42	88.05	88.88	87.09	88.88	88.88	0.7377
	FDG-PET		91.35	91.04	94.11	87.87	88.88	91.42	0.7610
	AV45-PET		90.48	90.48	82.31	87.5	92.31	92.31	0.7981
	rs-fMRI		92.31	92.53	94.28	90.62	91.66	92.95	0.8129
	DTI-FA		90	89.55	93.93	85.29	86.11	89.85	0.7557
	**Combined-ROI**		**94.93**	**94.24**	**100**	**88.89**	**92.31**	**96**	**0.8814**
HC vs. MCIs	APOE	EasyMKL	87.88	87.87	86.84	89.28	91.66	89.18	0.6855
	sMRI		90.11	89.06	90.90	87.09	88.23	89.55	0.7127
	FDG-PET		88.54	89.39	89.18	89.65	91.66	90.41	0.6984
	AV45-PET		92.31	92.42	94.28	90.32	91.66	92.95	0.7661
	rs-fMRI		90.62	90.12	93.75	87.5	88.23	90.90	0.7070
	DTI-FA		93.41	93.93	94.45	**93.33**	**94.44**	94.44	0.7854
	**Combined-ROI**		**95.6**	**95.55**	**100**	90.90	91.66	**95.65**	**0.8697**

Here, for AD vs. HC classification, the combined-ROI features performed very well compared with the single modalities. Similarly, for the single modalities, whole-brain MKL analysis for MCIs vs. MCIc (Table 2) using only the APOE genotype achieved 85.24% accuracy. Similar accuracy was obtained using FDG-PET (86.88%), AV45-PET (89.47%), and rs-fMRI (88.52%). Using sMRI-extracted ROI features, the achieved accuracy was lower (84.71%) in comparison with other single modalities. Using DTI-FA, the accuracy increased to 91.80% in comparison with both genotype and functional images. Moreover, when the combined-ROI features were passed through the classifier, the accuracy increased to 94.74% and the obtained Cohen’s kappa value was 0.8950 (Figure 5), which is closer to 1 than that for the individual modalities. Here, for the MCIs vs. MCIc classification, the combined-ROI features performed very well compared with the single modalities. Likewise, for the AD vs. MCIc classification problem, the best performance was attained using a combination of six modalities of features, which achieved an accuracy of 94.89% with a Cohen’s kappa of 0.8502. In this case, the rs-fMRI and DTI-FA unimodal features performed better for classifying the (AD vs. MCIc) group than the other unimodal features, and their obtained accuracies were 92.06 and 93.65%. For the AD vs. MCIs group, our proposed technique achieved 93.59% accuracy, which was 1.66% higher than the best accuracy obtained by the DTI-FA (unimodal) feature for classifying this group. The Cohen’s kappa score obtained for the AD vs. MCIs group was 0.8562 (Figure 5) which is close to the maximum agreement value of 1. For the HC vs. MCIc classification problem, our proposed method combining all six modalities of a biomarker to distinguish between HC and MCIc achieved good results compared with the single modality biomarkers. For this classification problem, our proposed method achieved 94.24% accuracy with a Cohen’s kappa of 0.8814 (Figure 5). In this case, from Table 2, we can see that all three (FDG-PET, AV45-PET, and rs-fMRI) functional imaging features performed well compared with the other unimodal features, and their obtained Cohen’s kappa scores were 0.7610, 0.7981, and 0.8129, which are all close to 1. Likewise, for the HC vs. MCIs group, our proposed technique achieved 95.55% accuracy, which is 1.62% higher than the best accuracy obtained by the DTI-FA (unimodal) feature for classifying this group. The obtained Cohen’s kappa score for the HC vs. MCIs group was 0.8697 (Figure 5), which is close to the maximum agreement value of 1. Therefore, from Table 2 and Figures 4, 5, we can state that for all classification combinations, our proposed method attained a high level of performance compared with the individual modality of biomarkers, varying from 1 to 3%, and our proposed scheme also attained a higher level of agreement between all six classification combinations than the individual modality-based methods.

The number of extracted ROIs was slightly higher for the sMRI, FDG-PET, and AV45-PET images than for the other modalities, although the real number of features used as input for each single and combined model varied across the models. Except for the HC vs. MCIc group, in the (AD vs. HC, MCIs vs. MCIc, AD vs. MCIc, AD vs. MCIs, and HC vs. MCIs) classification sets, it is interesting to observe that even though the number of extracted ROI features for sMRI, FDG-PET, AV45-PET, and rs-fMRI were higher than the number of DTI-FA ROI features, the obtained accuracy was lower than for the DTI-FA ROI features for the stated classification groups. Out of the 1404 ROIs, 384 ROIs selected from sMRI, 384 ROIs selected from FDG-PET, 384 ROIs selected from AV45-PET, 200 ROIs from rs-fMRI, 50 ROIs from DTI-FA, and the remaining two ROIs from APOE genotype corresponded to 27.3% (each of sMRI, FDG-PET, and AV45-PET), 14.5% (rs-fMRI), 3.5% (DTI-FA), and 0.1% (APOE) of the total number of features. In Figure 4, we show the ROC curves (plots of the TPR vs. the FPR for dissimilar possible cut-points) for each study presented in Table 2. The obtained AUC is presented in each plot. Figure 4 shows that our proposed method achieved higher AUC values for all classification sets than did the individual modalities. For the AD vs. HC and HC vs. MCIs classification groups, our proposed method achieved an AUC greater than 95%, while for the MCIs vs. MCIc, AD vs. MCIs, AD vs. MCIc, and HC vs. MCIc groups, the proposed method achieved an AUC less than 95%, (MCIs vs. MCIc, AD vs. MCIs, AD vs. MCIc, and HC vs. MCIc) < 95% < (AD vs. HC and HC vs. MCIs). The obtained result using the RBF-SVM classifier can be found in Supplementary Table S31. From Supplementary Table S31, we can say that the combined-ROI features performed very well as compared with the individual modality outcomes for all six binary classification groups. Table 2 and Supplementary Table S31 clearly show the advantage of using combined features over individual ones (see Supplementary Table S31).

### Classification Performance Across Single and Combined Modalities Using Voxel-Wise Analysis

**FIGURE 6:**
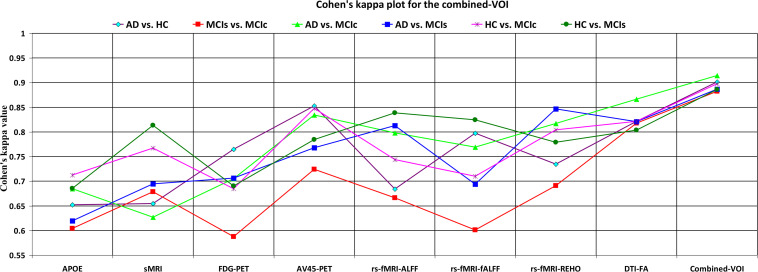
Cohen’s kappa plot for AD vs. HC, MCIs vs. MCIc, AD vs. MCIs, AD vs. MCIc, HC vs. MCIs, and HC vs. MCIc are grouped using voxel-wise analysis. The above graph clearly shows the benefit of the combined-VOI modality over any single modality.

For the voxel-wise analysis of the sMRI, FDG-PET, and AV45-PET images, we used the SPM12 toolbox with the integration of the CAT12 toolbox in MATLAB R2019a. For the DTI images, we used the DTIfit and TBSS functions from the FSL toolbox. For the voxel-wise analysis of rs-fMRI images, we used the DPARSF toolbox in MATLAB R2019a. Afterward, we passed these obtained features with two features from the APOE genotype data through a normalization technique to minimize the redundancy within the dataset. Furthermore, we passed these low-dimensional, normalized features from the polynomial kernel matrix to map them onto a high-dimensional feature space. Then we fused all these high-dimensional features in one form before passing them through the EasyMKL algorithm for classification. The obtained result are shown in Table 3 and Figure 6 shows the Cohen’s kappa plot for all six classification groups using voxel-wise analysis.

**TABLE 3:** Classification results for AD vs. HC, MCIs vs. MCIc, AD vs. MCIs, AD vs. MCIc, HC vs. MCIc, and HC vs. MCIs groups using voxel-wise (VOI) features (EasyMKL).

**Groups**	**Features**	**Classifier**	**Performance measure**						
			**AUC**	**ACC**	**SEN**	**SPEC**	**PRE**	**F1-score**	**Cohen’s kappa**
AD vs. HC	APOE	EasyMKL	88.18	87.71	88.89	83.33	80	84.21	0.6523
	sMRI		87.5	87.87	**100**	81.81	73.33	87.61	0.6547
	FDG-PET		91.35	89.39	92.59	87.17	83.33	87.71	0.7651
	AV45-PET		93.64	93.93	90.62	97.05	96.66	93.54	0.8528
	rs-fMRI-ALFF		89.81	90.12	92.85	89.47	86.66	89.65	0.6839
	rs-fMRI-fALFF		91.52	90.90	90	91.60	90	90	0.7973
	rs-fMRI-REHO		90.38	89.74	89.65	89.18	86.66	88.13	0.7345
	DTI-FA		92.86	92.42	93.10	91.89	90	91.52	0.8213
	**Combined-VOI**		**95.55**	**95.24**	93.33	**100**	**100**	**96.55**	**0.9014**
MCIs vs. MCIc	APOE	EasyMKL	85.56	85.24	84.37	86.20	87.09	85.71	0.6042
	sMRI		87.88	88.52	87.5	89.65	90.32	88.88	0.6788
	FDG-PET		83.33	84.21	85.71	80	92.31	88.89	0.5874
	AV45-PET		91.03	91.80	90.32	93.33	93.33	91.80	0.7243
	rs-fMRI-ALFF		87.78	89.47	**100**	83.33	77.78	87.5	0.6665
	rs-fMRI-fALFF		85.56	85.24	84.37	86.20	87.09	85.71	0.6011
	rs-fMRI-REHO		89.74	90.16	90.32	90	90.32	90.32	0.6907
	DTI-FA		92.31	92.75	90.65	93.10	93.54	92.06	0.8175
	**Combined-VOI**		**94.9**	**93.57**	92.86	**100**	**100**	**96.3**	**0.8825**
AD vs. MCIs	APOE	EasyMKL	78.89	77.68	72.73	75	80	76.19	0.6193
	sMRI		88.18	88.70	87.09	90.32	90	88.52	0.6944
	FDG-PET		90.91	90.32	90	90.62	90	90	0.7065
	AV45-PET		89.09	89.48	87.5	93.34	93.34	90.32	0.7679
	rs-fMRI-ALFF		92.73	92.45	90.32	93.54	93.33	91.80	0.8126
	rs-fMRI-fALFF		87.27	87.09	86.67	87.5	86.7	86.7	0.6943
	rs-fMRI-REHO		92.5	92.72	90	96	96.42	93.10	0.8467
	DTI-FA		90	91.93	93.10	90.90	90	91.52	0.8211
	**Combined-VOI**		**94.96**	**95.16**	**93.54**	**96.77**	**96.66**	**95.08**	**0.8869**
AD vs. MCIc	APOE	EasyMKL	86.9	85.21	80	88.89	88.89	84.21	0.6851
	sMRI		84.62	85.48	86.20	84.84	83.83	84.74	0.6272
	FDG-PET		88.54	88.89	85.29	93.10	93.54	89.23	0.7049
	AV45-PET		92.71	93.75	96.67	91.17	90.32	93.54	0.8342
	rs-fMRI-ALFF		90.62	90.47	87.87	93.34	93.54	90.62	0.7978
	rs-fMRI-fALFF		87.5	87.30	87.09	87.5	87.09	87.09	0.7691
	rs-fMRI-REHO		91.67	92.18	93.54	90.91	90.62	92.06	0.8173
	DTI-FA		93.75	93.65	88.57	**100**	**100**	93.93	0.8666
	**Combined-VOI**		**96.67**	**96.20**	**96.77**	96.87	96.77	**96.77**	**0.9145**
HC vs. MCIc	APOE	EasyMKL	85.45	85.71	83.33	88.89	90.91	86.96	0.7123
	sMRI		90.28	90.76	91.42	90	91.42	91.42	0.7673
	FDG-PET		86.36	86.88	86.48	86.67	88.89	87.68	0.6851
	AV45-PET		93.75	94.02	90.90	97.05	96.74	93.74	0.8481
	rs-fMRI-ALFF		90	90.36	91.42	87.5	88.89	90.14	0.7437
	rs-fMRI-fALFF		88.31	89.55	87.5	91.42	90.32	88.89	0.7099
	rs-fMRI-REHO		92.31	92.53	90.62	94.28	93.54	92.06	0.8043
	DTI-FA		93.18	92.87	88.23	**96.96**	**96.74**	92.30	0.8217
	**Combined-VOI**		**95.56**	**95.52**	**96.67**	94.59	93.54	**95.08**	**0.8977**
HC vs. MCIs	APOE	EasyMKL	87.88	87.87	86.84	89.28	91.66	89.18	0.6855
	sMRI		91.11	91.42	92.10	90.62	92.10	92.10	0.8136
	FDG-PET		85.23	85.33	86.45	82.85	85.36	86.41	0.6905
	AV45-PET		90	90.90	91.67	90	91.66	91.67	0.7847
	rs-fMRI-ALFF		92.86	92.30	94.28	91.14	91.67	92.95	0.8386
	rs-fMRI-fALFF		93.33	92.75	94.59	90.62	92.10	93.34	0.8248
	rs-fMRI-REHO		89.77	89.39	91.42	87.09	88.89	90.14	0.7788
	DTI-FA		91.68	90.14	89.74	91.25	91.32	90.90	0.8034
	**Combined-VOI**		**94.67**	**94.43**	**94.59**	**96.67**	**97.22**	**95.89**	**0.8864**

#### AD vs. HC

**FIGURE 7:**
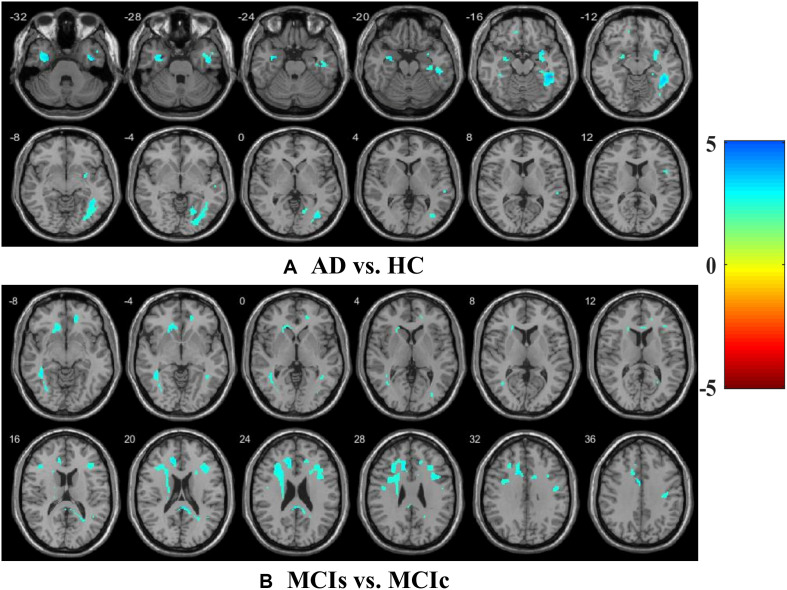
Affected region for **(A)** AD vs. HC, and **(B)** MCIs vs. MCIc is shown using AV45-PET image.

**FIGURE 8:**
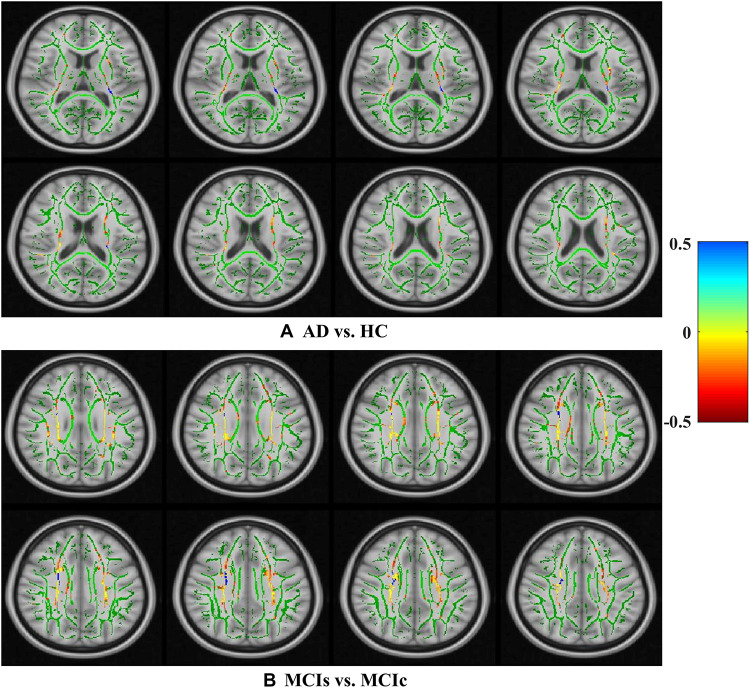
Selected WM voxels for the **(A)** AD vs. HC, and **(B)** MCIs vs. MCIc classification using DTI image.

We calculated the statistical values that represented the significance levels of the groups in the activation map as presented in Supplementary Tables S1–S5 after comparing the outcomes of the statistical two-sample *t*-tests for the AD vs. HC group. These tables specify the main affected area observed in the AD vs. HC set and the obtained voxel cluster with detailed information, including its peak areas in the form of the MNI space, cluster-level *p-score*, and the peak intensity in the *T-score* of each group. We used an uncorrelated threshold value of *p*_*uncorrected*_≤0.001 at the voxel level, an FDR value of *p*_*FDR*_ = 0.05, and an FWER value of *p*_*FWER*_ = 0.05 at the cluster level to achieve a bias alteration for multiple comparisons. An ROI binary mask was created from the selected clusters of each modality, and later the GM and WM volumes were removed from the two sets of images (AD vs. HC) (see Supplementary Tables S1–S5). Figure 7A shows the most significant regions where these groups differ from each other using an AV45-PET neuroimage, and the obtained voxel cluster is shown in Supplementary Table S3. Likewise, Figure 8A shows the most significant regions where these groups differ from each other using a DTI-FA neuroimage, and the obtained voxel cluster is shown in Supplementary Table S5 (see Supplementary Tables S3, S5).

#### MCIs vs. MCIc

Supplementary Tables S6–S10 show the main affected areas in the MCIs vs. MCIc group, the obtained voxel clusters with detailed information about its peak coordinates in the MNI space, the cluster-level *p-*score, and the peak intensity in the *T-*score of each group. For the MCIs vs. MCIc group, we used an uncorrelated threshold value of *p*_*uncorrected*_≤0.001 at the voxel level, an FDR value of *p*_*FDR*_ = 0.05, and an FWER value of *p*_*FWER*_ = 0.05 at the cluster level to accomplish a bias alteration for multiple comparisons. An ROI binary mask was created from the selected clusters of each modality and later GM and WM volumes were removed from the two sets of images (MCIs vs. MCIc) (see Supplementary Tables S6–S10). Figure 7B shows the most significant region where these groups differ from each other using an AV45-PET neuroimage, and the obtained voxel cluster is shown in Supplementary Table S8. Likewise, Figure 8B shows the most significant region where these groups differ from each other using a DTI-FA neuroimage, and the obtained voxel cluster is shown in Supplementary Table S10 (see Supplementary Tables S8, S10). Moreover, we followed the same procedure for the calculation of voxel clusters for the AD vs. MCIc, AD vs. MCIs, HC vs. MCIc, and HC vs. MCIs groups as we followed for the AD vs. HC and MCIs vs. MCIc groups. The obtained voxel clusters with detailed information about its peak coordinates in the MNI space, the cluster-level *p-score*, and the peak intensity in the *T*-score of each group are shown in Supplementary Tables S11–S30 (see Supplementary Tables S11–S30). Likewise, Supplementary Figures S1a,b show the most significant region for the AD vs. MCIc and HC vs. MCIc groups using the DTI-FA modality of the biomarker.

**FIGURE 9:**
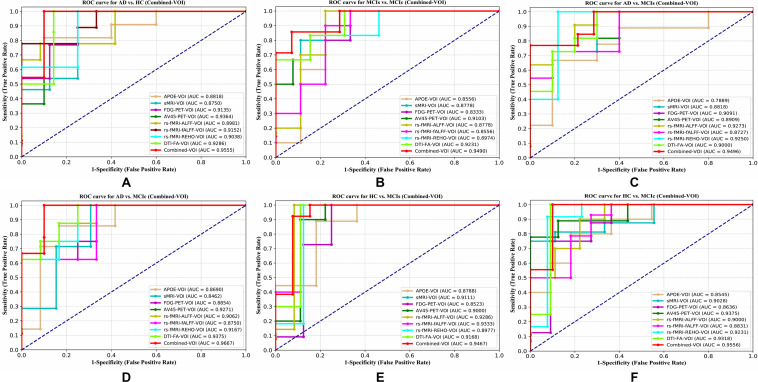
ROC curves for **(A)** AD vs. HC, **(B)** MCIs vs. MCIc, **(C)** AD vs, MCIs, **(D)** AD vs, MCIc, **(E)** HC vs. MCIs, and **(F)** HC vs. MCIc using voxel-wise (VOI) analysis. The red solid line shows the result of the combined-VOI curve, including all single-modality features.

The obtained voxel cluster for these two groups is shown in Supplementary Tables S15, S25 (see Supplementary Figures S1a,b and Supplementary Tables S15, S25). After completing the extraction of the series of features from each individual modality, we passed these obtained features through the polynomial kernel matrix to map these low-dimensional features onto a high-dimensional feature space. We then fused all these high-dimensional features in one form before passing them through the EasyMKL algorithm for classification. Table 3 shows the classification results for the AD vs. HC group using voxel-wise features. It shows that the combined features performed very well for this group compared with the single-modality results. The combined features achieved a 95.55% AUC for classifying the AD vs. HC group, as shown in Figure 9, with a Cohen’s kappa value of 0.9014, which is close to 1, as shown in Table 3 and Figure 6. This indicates that these two groups have a good level of agreement between them. Table 3 shows the classification results for the MCIs vs. MCIc group using voxel-wise features. This table also shows that the combined features performed very well for classifying the MCIs vs. MCIc group compared with the single-modality performances. The combined features achieved an AUC of 94.90% when classifying the MCIs vs. MCIc group, as shown in Figure 9. The Cohen’s kappa value was 0.8825, which is close to 1, as shown in Table 3 and Figure 6. This indicates a good level of agreement between the two groups. Furthermore, for the AD vs. MCIc and AD vs. MCIs classification groups, our proposed system attained a high level of performance and agreement (0.9145 and 0.8869) compared with the individual modalities biomarkers. For the AD vs. MCIc group, AV45-PET and DTI-FA attained high classification accuracy compared with other unimodal biomarkers, but their gained accuracy was 3% less than the accuracy gained by the combined-VOI process, which was 96.20% with a 0.9145 (Figure 6) Cohen’s kappa score. Likewise, for the AD vs. MCIs group, the proposed method achieved 95.16% accuracy with a 0.8869 (Figure 6) Cohen’s kappa score. For the HC vs. MCIc classification group, the AV45-PET individual modality biomarkers performed very well compared with the other single modality biomarkers. The obtained accuracy and Cohen’s kappa score using AV45-PET biomarkers were 94.02% and 0.8481 (Figure 6). Moreover, we then passed the combined-VOI features through the EasyMKL classifier for the classification of the HC vs. MCIc group, and after the classifier was applied, the accuracy increased by 1.5%. This suggests that the combined-VOI features were beneficial for classifying this group. Likewise, for the HC vs. MCIs classification group, the rs-fMRI features of ALFF and fALFF achieved a good level of performance and agreement (0.8386 and 0.8248) compared with the other individual modality biomarkers. In this case, the individual features of the APOE genotype also performed well compared with the FDG-PET imaging modality biomarker, but the individual modality biomarker’s performance was not very good compared with the combined-VOI result (Table 3). The combined-VOI features achieved a 94.43% accuracy and an AUC of 94.67% with a 0.8864 Cohen’s kappa score for classifying the HC vs. MCIs group. Figure 9 shows the ROC curve for all six classification groups; the red solid line in every classification task indicates the combined-VOI features for that particular group. Consequently, from Table 3 and Figures 6, 9, we can state that for all classification combinations, our proposed method attained a high level of performance compared with the individual modality biomarkers, varying from 1 to 3%, and our proposed scheme also attained a high level of agreement between each of the six classification combinations compared with the individual modality-based methods. The obtained result using the RBF-SVM classifier can be found in Supplementary Table S32. From Supplementary Table S32, we can say that the combined-VOI features performed very well as compared with the individual modality outcomes for all six binary classification groups. Table 3 and Supplementary Table S32 clearly show the advantage of using combined features over individual ones (see Supplementary Table S32).

While searching for the most significant region where AD vs. HC subjects differed from each other, we found that the (left/right) temporal-mid, (left/right) frontal-sup, (left/right) occipital-mid, (left/right) occipital-inf, (left/right) temporal-sup, (left/right) fusiform, (left/right) hippocampus, (left/right) temporal-inf, (left/right) precentral, and the (left/right) sagittal stratum were the regions where these subjects differed from each other most (Supplementary Tables S1–S5). Likewise, for the MCIs vs. MCIc group, we found that the (left/right) precentral, (left/right) precuneus, (left/right) frontal-mid, (left/right) cingulum-mid, (left/right) temporal-inf, (left/right) temporal-sup, (left/right) frontal-dup-medial, (left/right) cerebellum-9, (left/right) thalamus, and (left/right) fusiform were the regions where these subjects differed from each other most significantly (Supplementary Tables S6–S10). For the AD vs. MCIc group, we found that the (left/right) frontal-inf-tri, (left/right) frontal-inf-oper, (left/right) frontal-inf-orb, (right) hippocampus, (left/right) precentral, (left/right) thalamus, (left) pallidum, (left/right) lingual, and (left/right) inferior longitudinal fasciculus were the regions where these group subjects differed from each other most (Supplementary Tables S11–S15). As for the AD vs. MCIs group, we found that the (left/right) precentral, (left/right) frontal-mid, (left/right) hippocampus, (left/right) temporal-inf, (left/right) frontal-inf-orb, (left/right) occipital-mid, (right) posterior corona radiate, and (left/right) precuneus were the most significant regions where these group subjects differed from each other (Supplementary Tables S16–S20). For the HC vs. MCIc and HC vs. MCIs groups, we found that the (left/right) precentral, (left/right) cerebellum-6, (left/right) precuneus, (left/right) frontal-mid, (left/right) corticospinal tract, (left/right) lingual, (right) amygdala, and the (left/right) occipital-sup were the most significant regions where these two groups differed from each other (Supplementary Tables S21–S30). It is interesting to note that the (left/right) precentral region was found in every classification problem when computing voxel clusters (see Supplementary Tables S1–S30).

Table 4 shows the combined (VOI + ROI) classification results for all six classification groups after concatenating the extracted features from the whole-brain and voxel-wise methods with the APOE genotype. Before passing these features to the EasyMKL classifier, we applied a polynomial kernel matrix to map these low-dimensional features onto a high-dimensional feature space, so that every feature revealed its importance to the classifier. These high-dimensional features were fused into one form. We then passed these features to the MKL algorithm for classification.

**TABLE 4:** Classification result of AD vs. HC, MCIs vs. MCIc, AD vs. MCIs, AD vs. MCIc, HC vs. MCIc, and HC vs. MCIs groups using combined-(VOI + ROI) features, with both whole-brain and voxel-wise features (EasyMKL).

**Groups**	**Features**	**Classifier**	**Performance measure**						
			**AUC**	**ACC**	**SEN**	**SPEC**	**PRE**	**F1-score**	**Cohen’s kappa**
AD vs. HC	Combined-ROI	EasyMKL	95.45	96.05	94.11	100	100	96.96	0.9066
	Combined-VOI		95.55	95.24	93.33	100	100	96.55	0.9014
	**Combined (VOI + ROI)**		**97.78**	**98.52**	**96.97**	**100**	**100**	**98.46**	**0.9456**
MCIs vs. MCIc	Combined-ROI	EasyMKL	94.33	94.74	**100**	90.91	90	94.74	0.8950
	Combined-VOI		94.9	93.57	92.86	**100**	**100**	96.3	0.8825
	**Combined (VOI + ROI)**		**96.94**	**95.08**	**100**	93.93	93.54	**96.66**	**0.9247**
AD vs. MCIs	Combined-ROI	EasyMKL	93.86	93.59	93.75	93.33	93.75	93.75	0.8562
	Combined-VOI		94.96	95.16	93.54	96.77	96.66	95.08	0.8869
	**Combined (VOI + ROI)**		**96.25**	**96.68**	**94.12**	**100**	**100**	**96.97**	**0.9011**
AD vs. MCIc	Combined-ROI	EasyMKL	94.05	94.89	100	94.77	91.67	95.65	0.8502
	Combined-VOI		**96.67**	**96.20**	**96.77**	**96.87**	96.77	**96.77**	**0.9145**
	**Combined (VOI + ROI)**		95.56	95.23	93.94	96.68	**96.88**	95.39	0.9044
HC vs. MCIc	Combined-ROI	EasyMKL	94.93	94.24	**100**	88.89	92.31	**96**	0.8814
	Combined-VOI		95.56	95.52	96.67	**94.59**	93.54	95.08	0.8977
	**Combined (VOI + ROI)**		**96.67**	**96.65**	97.29	93.75	**94.74**	95.99	**0.9237**
HC vs. MCIs	Combined-ROI	EasyMKL	95.6	95.55	**100**	90.90	91.66	95.65	0.8697
	Combined-VOI		94.67	94.43	94.59	96.67	97.22	95.89	0.8864
	**Combined (VOI + ROI)**		**96.59**	**96.97**	93.75	**100**	**100**	**96.78**	**0.9187**

**FIGURE 10:**
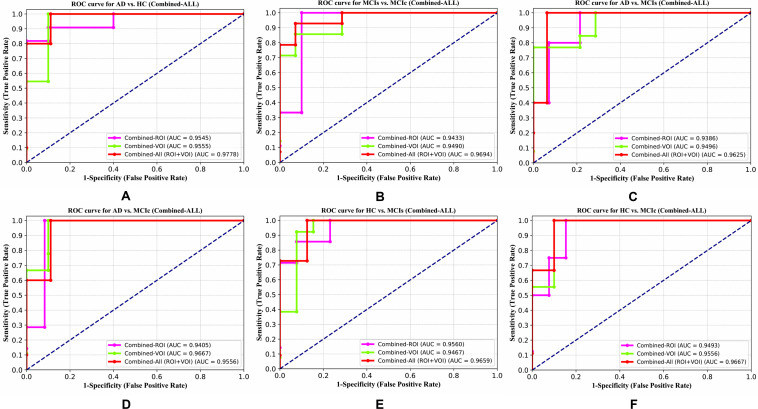
ROC curve for **(A)** AD vs. HC, **(B)** MCIs vs. MCIc, **(C)** AD vs, MCIs, **(D)** AD vs, MCIc, **(E)** HC vs. MCIs, and **(F)** HC vs. MCIc using combined-(VOI + ROI). The red solid line shows the result of the combined-(VOI + ROI) curve, with combined-ROI and combined-VOI features.

For the AD vs. HC classification group, the combined-(VOI + ROI) feature performed very well compared with the combined-VOI and combined-ROI features. The AUC and Cohen’s kappa scores were increased by 2–2.5% (97.78%, 0.9456) compared with those of combined-ROI and combined-VOI (Figure 10 and Table 4). Moreover, for the MCIs vs. MCIc classification group, Table 4 and Figure 10 show that the combined-(VOI + ROI) features achieved an AUC of 96.94% (2% increment), which was very high compared with the other two combined features. The obtained Cohen’s kappa value (0.9247) was also high compared with that of the combined-VOI and combined-ROI. Furthermore, for the AD vs. MCIs, HC vs. MCIs, and HC vs. MCIc classification groups, our proposed system performed very well compared with the results obtained by the combined-VOI and the combined-ROI for these groups. From Table 4, we can see that for these three (AD vs. MCIs, HC vs. MCIs, and HC vs. MCIc) groups, there is a 2–3% increment in every measured performance. The obtained AUC scores for these three groups are 96.25, 96.59, and 96.67%. Likewise, in the case of the AD vs. MCIc group, the Combined-ROI method performed very well compared with the combined-(VOI + ROI) and combined-VOI methods. The measured performance difference was not exceedingly high between the combined-ROI and the combined (VOI + ROI) method (just 1%) for the AD vs. MCIc group. The obtained result using the RBF-SVM classifier can be found in Supplementary Table S33. From Supplementary Table S33, we can say that the combined-(VOI + ROI) features performed very well as compared with the combined-ROI and combined-VOI features for all six binary classification groups. Table 4 and Supplementary Table S33 clearly show the advantage of using combined-(VOI + ROI) features over those of combined-ROI and combined-VOI (see Supplementary Table S33).

### Graph Network Construction and Analysis for All Six Classification Groups

**FIGURE 11:**
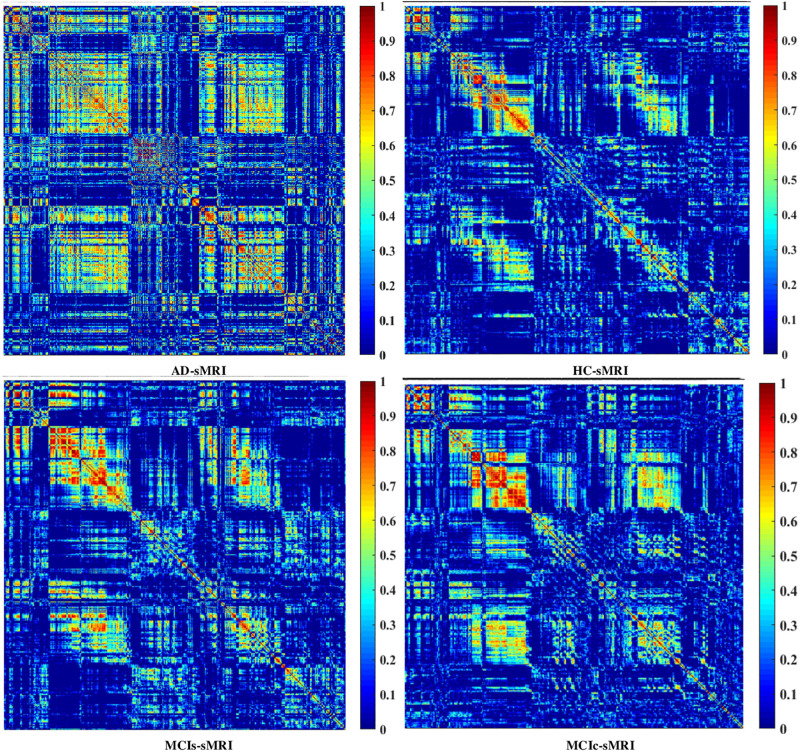
Weighted correlation matrices graph (of 384 regions) for AD, HC, MCIs, and MCIc for sMRI biomarkers.

**FIGURE 12:**
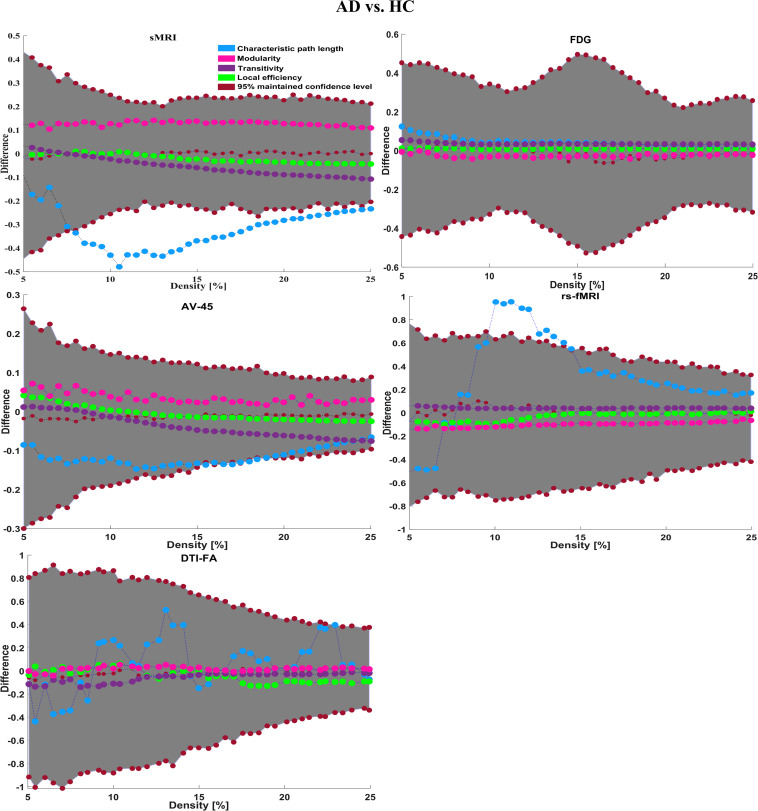
Differences between the AD vs. HC group in global structural topology. The blue sphere represents characteristic path length, green sphere represents local efficiency, pink sphere represents modularity, purple sphere represents transitivity, and the dark red sphere represents 95% confidence intervals for these measures.

**FIGURE 13:**
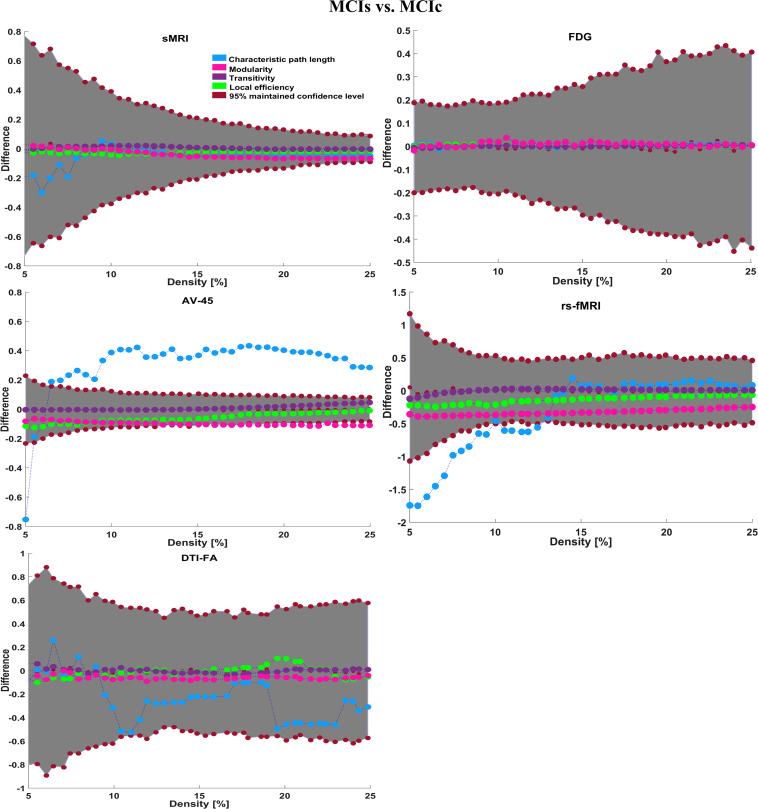
Differences between the MCIs vs. MCIc group and global structural topology. The blue sphere represents characteristic path length, green sphere represents local efficiency, pink sphere represents modularity, purple sphere represents transitivity, and the dark red sphere represents 95% confidence intervals for these measures.

For graph analysis or construction of a graph network, we used the BRAPH toolbox integrated into MATLAB 2019a. Additionally, we performed structural and functional graph theory for all six binary classification groups (AD vs. HC, MCIs vs. MCIc, AD vs. MCIc, AD vs. MCIs, HC vs. MCIc, and HC MCIs). Nodal measures were taken and comparisons were performed using the binary undirected graphs, and their measures were assessed over a set of network densities, which refers to the ratio between the number of connections in the network and the number of possible connections, ranging from 5–25% with a step size of 0.5%. Several graph metrics were calculated to quantify the nodal or global topological organization of the structural and functional networks, including local efficiency, characteristic path length, transitivity, and modularity. For all six binary classification groups, non-parametric permutation test samples with 1000 permutations each were conducted to assess the differences between the groups, which were significant for a two-tailed test of the null hypothesis at *p* < 0.05. The structural correlation matrices graph of AD, HC, MCIs, and MCIc of the sMRI subjects is shown in Figure 11. All groups showed strong correlations between bilaterally homologous regions. The plots in Figures 12, 13 and Supplementary Figures S2–S5 show the lower and upper bounds (dark red spheres) of the 95% confidence intervals (CI) (dark gray shade) as a function of density. The blue, green, pink and purple spheres show the differences between sets and, when falling beyond the CI, indicate that the change was statistically significant at *p* < 0.05.

The small dark red dot in the middle with a value around zero specifies the mean value of the change in the global network measures between the randomized sets after the permutation tests.

**FIGURE 14:**
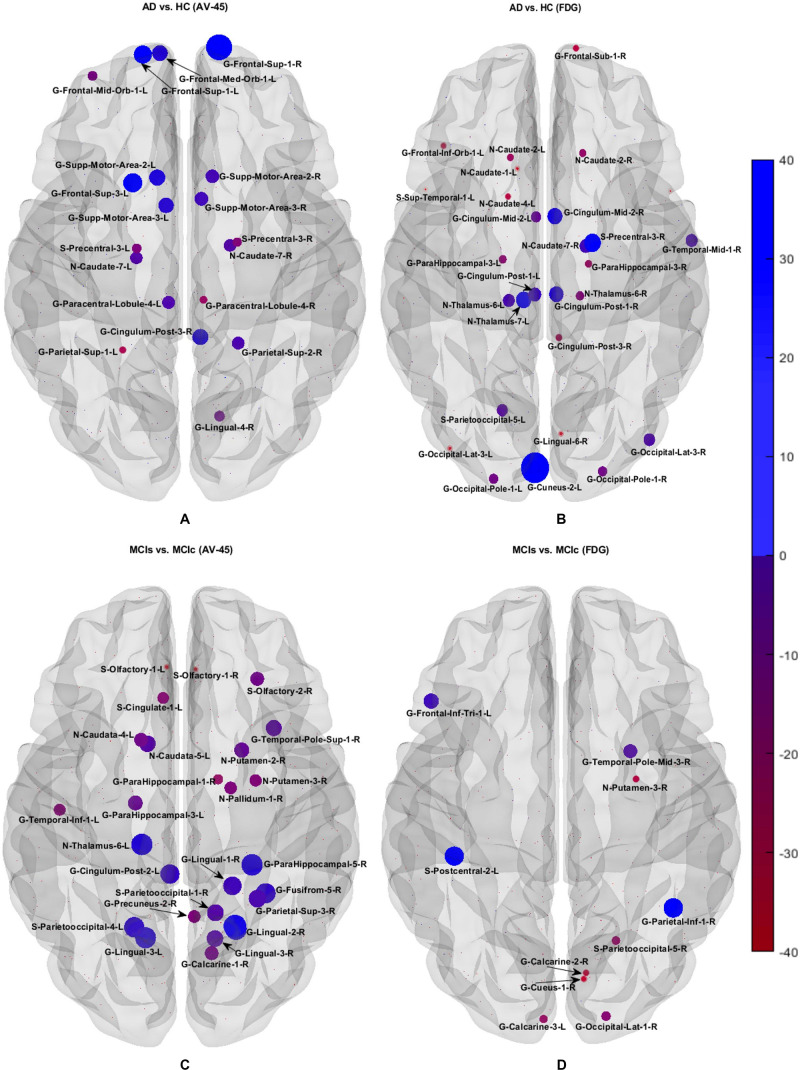
Brain maps representing the most predictive regions for distinguishing between the AD vs. HC and MCIs vs. MCIs groups. Differences between groups in nodal measures. Nodes showing significant differences among groups in the nodal degree after FDR corrections. For both classification groups, AV45 and FDG show the most significantly affected regions. Dark blue shows the most significant region, whereas light red indicates the least significant region.

We also compared the nodal degree for all six binary classification groups using all modalities. The FDR correction value was kept constant at 0.05 for all six binary classification groups. Regarding the global network topology shown for AD vs. HC in Figure 12, we found a longer characteristic path length using only FDG (which started at 0.15) than that with other neuroimaging modalities, and the path length was above the mean value of the difference. In the case of local efficiency, we found that the AV45 modality was the only one that started (at 0.05) above the mean value. Moreover, by comparing the modularity graph in the AD vs. HC group, we found that sMRI showed the greatest difference (modularity started at 0.14) of all modalities, and its network densities were almost constant until 25% (showing that the network topology is widely spread). The rs-fMRI modality performed very well for the transitivity graph compared with the other modalities, starting from 0.06 and increasing to 25%, although it decreased in some network densities. Moreover, we also computed the regional or nodal network topology for the AD vs. HC group, which is shown in Figures 14A,B. Figures 14A,B show that the AV45 and FDG-PET modalities are the only one neuroimage that shows the numbers of significant region changes in a nodal network topology for the AD vs. HC group. The nodal degree showed significant increases in the right g-frontal-sup-1, left g-cuneus-2, left g-frontal-sup-3, left g-frontal-sup-1, left g-frontal-med-orb-1, and right s-precentral-3 regions. Likewise, for the MCIs vs. MCIc group, we found increases in the characteristic path length and local efficiency using FDG (in both cases, it started at 0.01, as shown in Figure 13). For the modularity, sMRI attained 5% network density, but after that, it decreased to 25%. At the same time, the modularity in DTI neuroimages increased in network density from 10 to 25%. Likewise, for transitivity, compared with other modalities that started below the mean value, the AV45 modality was the only modality that started from 0 (difference), and its network increased in every single network density.

This transitivity plot shows the most widespread topological changes for the MCIs vs. MCIc group. Figures 14C,D show that the AV45-FDG is the most important modality to show the numbers of significant region changes in the nodal topology of the MCIs vs. MCIc group. Left s-postcentral-2, right g-parietal-inf-1, left g-frontal-inf-tri, right g-lingual-2, right g-parahippocampus-5, left n-thalamus-6, left s-parietooccipital-4, left-lingual-3, and left g-cingulum-post-2 are the most significant regions shown by the nodal degree for the MCIs vs. MCIc classification group. For the AD vs. MCIc classification group, the global network topology is shown in Supplementary Figure S2. The rs-fMRI is the only modality that shows an increment in the characteristic path length measure. It starts at 0.38 (difference), and lies inside the CI and above the mean value, but at 15–17% network density, some of its networks lie outside of the CI upper bound, and again from 18% density, its network lies inside the CI until 25% density is reached. We can also see that at around 13% network density, some of its networks are close to the mean value (which is shown by small dark red dots). It shows the most widespread topological changes for the AD vs. MCIc group. The FDG-PET modality shows an increase only for the local efficiency measure. Modularity and transitivity increase across almost all network densities for the sMRI and AV45-PET modality compared with the other individual modalities. Supplementary Figures S6a,b show that AV45 and rs-fMRI modalities are the only one that shows the numbers of significant region changes in a nodal network topology for the AD vs. MCIc group. The nodal degree shows significant increases in the left g-lingual, (left/right) g-lingual-3, (left/right) occipital pole, right cerebellum, left n-caudate-5, right n-caudate-2, left n-thalamus-2, and left frontal pole regions (see Supplementary Figures S2, S6). Likewise, for the AD vs. MCIs group, the global network topology is shown in Supplementary Figure S3. The FDG-PET modality is the only one showing a characteristic path length that lies inside the CI and above the mean value. In comparing local efficiency measures in every modality, we found that the rs-fMRI modality was the only one neuroimage that lies above the mean value (which is plotted by small dark red), although every modality lies inside the CI. The modularity increased, and at the same time, the transitivity decreased for the AD vs. MCIs group, as shown by the sMRI modality, but both plots lie inside the 95% CI. The right n-caudate-2, left g-insula-anterior-2, left g-frontal-mid-orb-1, left g-cuneus-2, right g-angular-3, right g-occipital-pole-1, right n-thalamus-6 were the most significant regions as shown by the nodal degree for the AD vs. MCIs classification group using FDG and sMRI images (Supplementary Figures S3, S6c,d). The global network topology is plotted in Supplementary Figure S4 for the HC vs. MCIc classification group. From this plot we can see that the characteristic path length begins at 0.8 (difference) at a 5% density in the rs-fMRI modality (which is very high compared with other modalities). However, it suddenly begins to decrease at 8–13% density, and crosses lower bound (represented by a dark red sphere), and again later, it increases at 14% network density. The rs-fMRI modality is the only one neuroimage where the local efficiency, modularity, and transitivity lie above the mean values (small dark red sphere). Moreover, this plot lies in the middle of the 95% confidence interval. Supplementary Figures S7a,b show that the DTI-FA and rs-fMRI modalities are the only one neuroimage that show the numbers of significant region changes in the nodal network topology for the HC vs. MCIc group. The nodal degree shows significant increases in the right inferior-parietal-g, left superior occipital-g, right brainstem, right midbrain, left postcentral-g, left parahippocampal-g, left corticospinal tract, (left/right) posterior limb of the internal capsule, and the left uncinate fasciculus regions (see Supplementary Figures S4, S7). For the HC vs. MCIs classification group, the global network topology is shown in Supplementary Figure S5. In this case, characteristic path length using the sMRI modality is the only one that lies above the mean value. The local efficiency increases with both (sMRI and rs-fMRI) modalities, and their network lies above the mean value (which is represented by the small dark red sphere). The modularity plot shows that the rs-fMRI image is the only one in which there is an increment in the network density from 6 to 25%, whereas in the same image, the transitivity decreases from 6 to 25% density. However, both of these networks lie inside the CI. Supplementary Figures S7c,d show that the sMRI and FDG modalities are the only one neuroimage that shows the numbers of significant region changes in a nodal network topology for the HC vs. MCIs group. The nodal degree shows significant increases in the right g-cuneus-2, left g-occipital-sup-1, left g-Supp-motor-area-1, left n-Caudata-6, left n-thalamus-2, right n-thalamus-1, and the right temporal-pole-mid-3 regions (see Supplementary Figures S5, S7).

## Discussion

The present study outcomes provided insights into multimodal behavior for classifying all six binary classification groups, and it also showed which regions or single modality are most significant for future analysis for the discrimination of AD at a clinical level. Here, our proposed idea was to combine multiple neuroimaging modalities (sMRI, AV45, FDG, rs-fMRI, and DTI) with a genetic biomarker (APOE) for the classification of AD patients and other groups using whole-brain, voxel-wise, and graphical analysis methods. In this study, we utilized three different types of atlas (AICHA, pyClusterROI, and JHU-WM) to parcellate the sMRI/PET, rs-fMRI, and DTI neuroimages. Furthermore, to parcellate the sMRI and PET images, we employed the AICHA atlas, which is already segmented into 384 ROIs. Likewise, for rs-fMRI neuroimages, we applied pyClusterROI Python script with the Craddock atlas to parcellate the rs-fMRI images into 200 brain regions (because this is a well-known technique for parcellating fMRI images using a spatially constrained normalized-cut spectral clustering process). Moreover, we also knew that fMRI images are made up of a time series (ADNI rs-fMRI data is made up of 140 time series or time points), so to parcellate the brain using fMRI data, voxels with similar time points needed to be grouped to form a region. This is typically done using data-driven clustering methods, where each cluster constitutes one region. For this reason, we chose the pyClusterROI script for the rs-fMRI images. Moreover, for DTI images, we applied the JHU-WM (ICBM-DTI-81) label atlas, which was already segmented into 50 brain regions (these 50 WM tract labels were created by the hand segmentation of a standard-space average of the diffusion MRI tensor maps from 81 subjects). In this study, we selected different atlases for the different modalities of neuroimages because of their advantages, and also for the extraction of higher brain ROIs for that particular modality of neuroimage. After the completion of feature extraction from each method, we sent these low-dimensional extracted features through a polynomial kernel function to map them onto a high-dimensional feature. Afterward, we fused all six high-dimensional features into one form before further analysis. Later, we passed these unimodal and multimodal features through the EasyMKL classifier for classification and reported the average accuracy for each method. This procedure is widely used for comparing the performance of machine learning approaches. Although previous studies have already applied the multimodal method for the classification of AD (Zhang et al., 2011; Young et al., 2013; Liu et al., 2014; Ritter et al., 2015; Schouten et al., 2016; Hojjati et al., 2018; Gupta et al., 2019a) with other groups, this study was the first to combine five different types of neuroimage modalities with two APOE genotype scores for the classification of all six binary classification groups (AD vs. HC, MCIs vs. MCIc, AD vs. MCIc, AD vs. MCIs, HC vs. MCIc, and HC vs. MCIs). Our proposed method clearly showed an improvement using multimodal features in terms of performance over the unimodal features for classifying these six binary groups compared with the latest published results. Furthermore, in this study, we also adopted graph-theoretical strategies (in a global and nodal network topology) to study the plots of the six binary groups (characteristic path length, local efficiency, modularity, and transitivity), and to find the most significant regions where these groups differed from each other.

### Influence of the Different Types of Neuroimage Modality

We compared the Cohen’s kappa score obtained for each modality using both ROI and VOI features. The score was calculated for each of the six classification groups. The Cohen’s kappa outcomes, displayed in Figure 5, were computed using ROI-based features. Likewise, the Cohen’s kappa outcomes displayed in Figure 6 were computed using VOI-based features. The results displayed in Figures 5, 6 clearly show that the DTI-FA modality biomarker achieved a high level of agreement between groups while classifying the six binary classification groups compared with the other five biomarkers. Furthermore, we can say that VOI-based DTI-FA (lies above 0.8–0.87) feature performed slightly better than the ROI-based DTI-FA (lies above 0.75–0.84) feature.

### Influence of the Type of Features (ROI and VOI)

We compared the Cohen’s kappa score obtained for the regional (ROI) features with the reference atlases (AICHA, Craddock, and JHU-WM) to the ones obtained for the voxel (VOI) features (SPM12, DPARSF, TBSS) for five different types of neuroimages using the EasyMKL classifier. The score was evaluated for the same six binary classification groups. The Cohen’s kappa outcomes displayed in Figure 5 are for an ROI-based analysis, and likewise, the Cohen’s kappa outcomes displayed in Figure 6 are for a VOI-based analysis. The results, displayed in Figures 5, 6, do not show notable differences in the Cohen’s kappa scores obtained using regional or voxel features; both features performed very well and achieved a high level of agreement between each other for all six binary classification groups.

### Influence of the Classification Method

**FIGURE 15:**
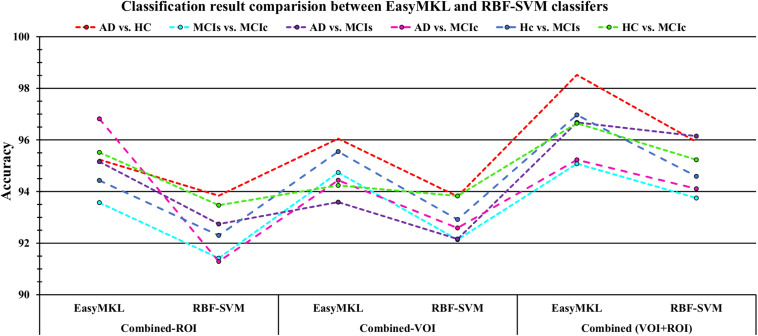
Comparison of EasyMKL classifier results with RBF-SVM classifier results based on obtained accuracy score. In the above figure, we can see that the combined-ROI, combined-VOI, and combined-(VOI + ROI) result obtained by EasyMKL classifier outperformed the results [combined-ROI, combined-VOI, and combined-(VOI + ROI)] obtained by the RBF-SVM classifier for all six classification groups.

We compared the result obtained by the EasyMKL classifier with that of the RBF-SVM classifier. Tables 2–4 and Supplementary Tables S31–S33 show the obtained classification result for all six binary groups using the EasyMKL and RBF-SVM classifiers. Likewise, Figure 15 shows the plot where we compared the accuracy obtained for all six binary classification tasks using the EasyMKL and RBF-SVM classifiers. The result displayed in Figure 15 shows that the EasyMKL classifier achieved high classification accuracy for all six binary groups compared with RBF-SVM. It also suggests that EasyMKL optimized a simple quadratic problem addressed by SVM in a more efficient way (see Supplementary Tables S31–S33).

### Most Significant Brain Regions Where Each Group Differs From the Others

Table 5 shows the obtained most important brain regions for each binary classification group, using voxel-wise and graphical methods. For the AD vs. HC group, using VOI analysis, we found that (L) Thalamus, (L) Occipital Inferior, (R) Fusiform, (R) Temporal Inferior, (R) Temporal Mid, (R) Temporal Inferior, and (L) Sagittal stratum were the brain regions where the AD group differs from the HC group. Likewise, using graphical analysis, we found that (L) G-Cuneus-2, and (R) G-Frontal-Superior-1 were the brain regions where the AD group differs from the HC group. Furthermore, for the MCIs vs. MCIc group, using VOI analysis, we found that (L) Precuneus Vermis_9, (L) Frontal Superior Medial, (L) Pallidum, (R) Lingual, (L) Frontal Mid, (R) External capsule were the brain regions where the MCIs group differs from the MCIc group. Likewise, after applying graphical analysis, we found that the (R) G-Parietal Inferior-1, and (R) G-Lingual-2 were the brain regions where the MCIs group differs from the MCIc group. For the AD vs. MCIs group, using the VOI method, our study found that the (L/R) Frontal Inferior Triangular, (R) Temporal Superior, (R) Frontal Inferior Orbital, (R) Thalamus, (R) Hippocampus, and (R) Anterior thalamic radiation were the brain regions where the AD group differs from the MCIs group. Moreover, after applying the graphical method, we found that the (R) N-Caudate-2 and (L) G-Cuneus-2 were the brain regions where the AD differs from the MCIs the most. Furthermore, for the AD vs. MCIc group, we found using VOI analysis that the (R) Frontal Middle orbital, (R) Occipital Middle, (R) Precentral, (L) Fusiform, (L) Occipital Middle, (L) Cingulum Posterior, and (R) Posterior corona radiata were the brain regions where the AD group differs from the MCIc group. Likewise, after applying graphical analysis, we found that the (L) N-Caudate-5 and (L) G-Lingual were the brain regions where the AD group differs from the MCIc group. For the HC vs. MCIs group, using the VOI method, our study found that the (R/L) Precentral, (L) Cerebelum_9, (L) Parietal Inferior, (L) Putamen, and (L) Superior longitudinal fasciculus were the brain regions where the HC group differs from the MCIs group. Moreover, after applying the graphical method, we found that the (L) N-Caudate-6 and (L) G-Occipital Superior-1 were the brain regions where the HC differs from the MCIs the most. Likewise, for the HC vs. MCIc group, using the VOI method, we found that the (L) Lingual, (L) Occipital Superior, (R) Frontal Inferior Opercular, (L) Frontal Inferior Orbital, (R) Frontal Superior Orbital, (L) Lingual, and (L) Superior corona radiata were the brain regions where the HC group differs from the MCIc group the most. Moreover, using graph-based analysis, we found that the (R) G-Parietal Inferior and (L) Corticospinal tract were the brain regions where the HC group differs from the MCIc group the most. It is interesting to note that most of the affected brain regions lies in the left hemisphere rather than the right hemisphere.

**TABLE 5:** Most significant brain regions found for all six binary classification groups using voxel-wise and graphical analysis.

**Groups**	**Modality**	**Found to be the most significant brain region by applying VOI analysis**	**Modality**	**Found to be the most significant brain region by applying graphical analysis**
AD vs. HC	sMRI	(L) Thalamus		
	FDG-PET	(L) Occipital Inferior		
	AV45-PET	(R) Fusiform		
	rs-fMRI-ALFF	(R) Temporal Inferior	FDG-PET	(L) G-Cuneus-2
	rs-fMRI-fALFF	(R) Temporal Mid	AV45-PET	(R) G-Frontal-Superior-1
	rs-fMRI-REHO	(R) Temporal Inferior		
	DTI-FA	(L) Sagittal stratum		
MCIs vs. MCIc	sMRI	(L) Precuneus		
	FDG-PET	Vermis_9	FDG-PET	(R) G-Parietal Inferior-1
	AV45-PET	(L) Frontal superior Medial	AV45-PET	(R) G-Lingual-2
	rs-fMRI-ALFF	(L) Pallidum		
	rs-fMRI-fALFF	(R) Lingual		
	rs-fMRI-REHO	(L) Frontal Mid		
	DTI-FA	(R) External capsule		
AD vs. MCIs	sMRI	(R) Frontal Inferior Triangular	sMRI	(R) N-Caudate-2
	FDG-PET	(L) Frontal Inferior Triangular	FDG-PET	(L) G-Cuneus-2
	AV45-PET	(R) Temporal Superior		
	rs-fMRI-ALFF	(R) Frontal Inferior Orbital		
	rs-fMRI-fALFF	(R) Thalamus		
	rs-fMRI-REHO	(R) Hippocampus		
	DTI-FA	(R) Anterior thalamic radiation		
AD vs. MCIc	sMRI	(R) Frontal Middle Orbital		
	FDG-PET	(R) Occipital Middle		
	AV45-PET	(R) Precentral	AV45-PET	(L) N-Caudate-5
	rs-fMRI-ALFF	(L) Fusiform	rs-fMRI	(L) G-Lingual
	rs-fMRI-fALFF	(L) Occipital Middle		
	rs-fMRI-REHO	(L) Cingulum Posterior		
	DTI-FA	(R) Posterior corona radiata		
HC vs. MCIs	sMRI	(R) Precentral	sMRI	(L) N-Caudate-6
	FDG-PET	(L) Precentral	FDG-PET	(L) G-Occipital Superior-1
	AV45-PET	(L) Cerebellum_9		
	rs-fMRI-ALFF	(L) Precentral		
	rs-fMRI-fALFF	(L) Parietal Inferior		
	rs-fMRI-REHO	(L) Putamen		
	DTI-FA	(L) Superior longitudinal fasciculus		
HC vs. MCIc	sMRI	(L) Lingual		
	FDG-PET	(L) Occipital Superior		
	AV45-PET	(R) Frontal Inferior Opercular		
	rs-fMRI-ALFF	(L) Frontal Inferior Orbital	rs-fMRI	(R) G-Parietal Inferior
	rs-fMRI-fALFF	(R) Frontal Superior Orbital	DTI-FA	(L) Corticospinal tract
	rs-fMRI-REHO	(L) Lingual		
	DTI-FA	(L) Superior corona radiata		

### Exploring the Improvement in Performance Using Multimodal Features Compared With Unimodal Features

In this study, we applied two different types of methods for the classification of AD with another group. In the first method, we used the whole-brain parcelation approach to segment the brain images according to the atlas using some toolbox (see section “Three Feature Extraction Processes”) and later, after the completion of the data pre-processing step, we sent these features through the MKL algorithm to classify AD and the other groups. To verify the efficiency of this proposed method, we calculated the performance of each classification group using both unimodal and combined-ROI (or multimodal) features. Table 2 shows the obtained classification results for all six binary classification groups using whole-brain analysis features. From Table 2, we can say that the combined features (combined-ROI) performed very well compared with the single-modality features in every classification problem. Their obtained Cohen’s kappa values were close to 1, which indicates that our proposed method achieved a good level of agreement between groups while classifying each set. Table 2 further shows that the DTI-FA unimodal features attained high classification performance for every classification group except for the HC vs. MCIc group (where rs-fMRI features achieved better performance than DTI-FA features) in comparison with the performance of other individual modalities. This result demonstrates that the DTI-FA image can be used as a notable biomarker when investigating AD, and therefore should be included in clinical research.

Likewise, in the second step, we utilized the voxel-wise approach for the extraction of features from sMRI, FDG, AV45-PET, rs-fMRI, and DTI images using some toolbox (see section “Three Feature Extraction Processes”) and later, after the completion of data pre-processing step, we sent these features through the MKL classifier to classify AD with the other groups. To check the efficiency of this proposed method, we calculated the performance of each classification group using both unimodal and combined-VOI (or multimodal) features. Table 3 shows the obtained classification results for all six binary classification groups using voxel-wise analysis features. From Table 3, we can say that our proposed approach (to combine all unimodal features into one before passing them through the MKL classifier) gained a high level of performance and agreement compared with the unimodal results for every classification group. Table 3 further suggests that AV45-PET, rs-fMRI (ALFF, REHO), and DTI-FA individual features performed very well compared with sMRI, APOE, and FDG-PET individual modalities. It is interesting to note that the DTI-FA modality performed well for both whole-brain and voxel-wise analysis methods.

Tables 2, 3 satisfied our hypothesis by showing that classification performance increased by 2–4% in every classification group after we passed concatenated features (combined-ROI and combined-VOI) through the MKL classifier. Moreover, the obtained result demonstrates that the multimodal method is more powerful than the unimodal system, and the obtained outcomes also indicate that every single biomarker has some important information, so it is better to combine the complementary information together for the classification of AD. Furthermore, we also examined the performance of every six classification groups by concatenating both ROI and VOI features into a single form. Table 4 shows the obtained results for all six binary classification groups using combined (VOI + ROI) features. From Table 4, we can say that after applying combined (VOI + ROI) features, the classification performance increased slightly for every classification group except the AD vs. MCIc group (where combined-VOI dominated the combined-ROI and combined (VOI + ROI) methods in terms of performance). Their obtained Cohen’s kappa values were close to 1, which indicates that our proposed scheme achieved a good level of agreement between groups while classifying each set. Supplementary Tables S1–S30 show the most significantly affected regions for all six binary classification groups measured by the sMRI, FDG-PET, AV45-PET, rs-fMRI, and DTI-FA modalities of neuroimage.

It is interesting to note that the (left/right) precentral region was found in every classification group when extracting the most significant voxel region. This finding suggests that, in the coming days, the precentral region can also be used as an important biomarker for the classification of AD and other groups.

### Global and Nodal Network Topology Result Analysis

We studied the global and nodal network topology for all six binary classification groups using graph theory. We calculated characteristic path length, local efficiency, modularity, and transitivity for every classification group, which can be seen in Figures 12, 13 and Supplementary Figures S2–S5. In the global network topology analysis, our study found that in general, FDG, sMRI, and rs-fMRI were the modalities where characteristic path length increased efficiently compared with the other modalities. Likewise, for local efficiency, AV45, FDG-PET, rs-fMRI, and sMRI were the modalities where it increased compared with the remaining modalities. It is interesting to note that modularity increased in every classification group using sMRI neuroimages, except for the HC vs. MCIs group (where modularity increased efficiently by utilizing rs-fMRI images instead of sMRI images). Furthermore, in general, rs-fMRI, AV45-PET, and sMRI were the modalities where transitivity increased and decreased frequently. These changes symbolize that the regions of their networks communicated (more or less) efficiently within other brain regions. The increments in modularity (suggesting that their modules should have higher within-module connectivity and poor inter-module connectivity, or vice-versa) and increment or decrement of transitivity graph (suggesting that the regions of their network were connected very well to neighboring areas, or vice-versa). In the nodal network topology analysis, our study found that every neuroimage was important to finding the most significant region where the six binary classification groups differ from each other; the found regions can be seen in Figure 14 and Supplementary Figures S6, S7. The most significant regions were right g-frontal-sup-1, right s-precentral-3, left g-frontal-inf-tri, (left/right) n-thalamus-6, left s-parietooccipital-4, left g-cingulum-post-2, (left/right) occipital pole, left n-thalamus-2, left g-insula-anterior-2, (left/right) g-cuneus-2, left corticospinal tract, left n-Caudata-6, right temporal-pole-mid-3, left lingual-g, right fusiform, left g-precuneus-8, right n-Caudata-2, and (left/right) g-parahippocampal-4.

Recently, several studies have investigated neuroimaging techniques for the discrimination of AD, with the main focus on MCI subjects, who may or may not convert to AD, and separating patients with AD from healthy controls. However, it is difficult to make direct comparisons with these state-of-the-art methods because most of the studies used different validation methods and datasets, which both influence the classification performance. The first study by Cui et al. (2011) obtained an AUC of 79.6% for the classification of MCIs vs. MCIc groups. The authors used multimodal features (NM: neuropsychological and functional measures, CSF, and sMRI) for the classification. Young et al. (2013) used a Gaussian process method with SVM for the classification of MCIs vs. MCIc with three modalities (MRI, PET, and APOE). They reported an accuracy of 69.9% for classifying MCIs vs. MCIc groups. In another study, domain transfer learning was introduced using multimodal data (i.e., MRI, CSF, and FDG) with an accuracy of 79.4% for MCIs vs. MCIc with an AUC of 84.8% (Cheng et al., 2015). In another study by Xu et al. (2016), the authors used three different imaging modalities (sMRI, FDG, and AV45) for the discrimination of the conversion of MCIs subjects to MCIc patients. The authors used a weighted multi-modality sparse representation-based classification (wmSRC) classifier for classification. Their method achieved an ACC of 82.5% for classifying MCIs vs. MCIc with a sensitive biomarker selected using different numbers (from 1 to 90) of ranked features from each modality. Liu et al. (2017) proposed an algorithm that combines two imaging modalities of independent component study and the Cox model for discrimination of MCI progression. Their method achieved 80.8% of AUC with 73.5% of accuracy when classifying MCIs vs. MCIc. Hojjati et al. (2017) used a subset of optimal features in an SVM classifier for the discrimination of the conversion of MCIs to MCIc subjects. Their proposed method with multivariate minimal redundancy maximal relevance feature selection achieved 94.9% AUC and 91.4% ACC. In another study, Pan et al. (2019b) used an ensemble classification with a feature ranking method for the classification of MCIs vs. MCIc. Their proposed method achieved 75.38% AUC and 80.70% ACC for classifying this group. Gupta et al. (2019a) used a multimodal feature (sMRI, FDG, CSF, and APOE) for the discrimination of the conversion from MCIs to MCIc. They used the NiftyReg toolbox for the extraction of 246 ROIs from each imaging modality and applied a kernel-based SVM for the classification of MCIs vs. MCIc. Their method achieved an AUC of 93.59% for classifying these groups. Table 6 shows the comparison result for MCIs vs. MCIc classification. Our proposed multimodal method [combined-(ROI + VOI)] outperforms the latest published state-of-the-art methods in terms of AUC and ACC for MCIs vs. MCIc. The proposed method achieved 96.94% AUC and 95.08% ACC for classifying the MCIs vs. MCIc group.

**TABLE 6:** Performance comparison for MCIs vs. MCIc classification group.

**Method**	**Modality**	**Total sample size**	**AUC**	**ACC**
Cui et al., 2011	NM + CSF + sMRI	143	79.6	67.13
Young et al., 2013	sMRI + FDG + APOE	143	79.5	69.9
Cheng et al., 2015	MRI + FDG + CSF	99	84.8	79.4
Xu et al., 2016	sMRI + AV45 + FDG	110	-	82.5
Liu et al., 2017	sMRI + FDG	234	80.8	73.5
Hojjati et al., 2017	rs-fMRI	80	94.9	91.4
Pan et al., 2019b	FDG	248	80.70	75.38
Gupta et al., 2019a	APOE + sMRI + FDG + CSF	82	93.59	94.86
Combined-(ROI + VOI)	sMRI + FDG + AV45 + rs-fMRI + DTI + APOE	61	**96.94**	**95.08**

## Conclusion

The novelty of the present study is that we combined five neuroimaging modalities (sMRI, FDG-PET, AV45-PET, rs-fMRI, and DTI) with the APOE genotype score for the discrimination between AD and other groups. Furthermore, we employed three different approaches [whole-brain (ROI), voxel-wise (VOI), and graph-based] on six binary classification groups to analyze each method performance independently. The combined-(ROI + VOI) feature performance outperformed the combined-ROI and combined-VOI features for all classification groups except the AD vs. MCIc group (where combined-VOI performed well). When the performance of each imaging modality in brain graph analysis was compared for all six binary classification sets, we found that FDG-PET, AV45-PET, and rs-fMRI were the only three modalities that revealed the most affected brain regions for all six classification groups. These highlighted central brain regions are an early indicator of developing dementia in healthy subjects.

## Data Availability Statement

The datasets used in this study were acquired from the ADNI homepage, which is available freely for all researcher and scientist for experiments on Alzheimer’s disease and can be easily downloaded from ADNI websites: http://adni.loni.usc.edu/about/contact-us/. Furthermore, all datasets presented in this study are included in the article/Supplementary Material.

## Ethics Statement

As per ADNI protocols, all procedures performed in studies involving human participants were in accordance with the ethical standards of the institutional and/or national research committee and with the 1964 Helsinki declaration and its later amendments or comparable ethical standards. More details can be found at adni.loni.usc.edu. (This article does not contain any studies with human participants performed by any of the authors).

## Author Contributions

YG and J-IK designed the study and collected the original imagining data from the ADNI homepage. YG wrote the manuscript and did the coding section. J-IK and G-RK analyzed the imaging dataset. YG and BK analyzed the obtained results. All authors contributed and approved the final manuscript.

## Footnotes

1 https://www.adni-info.org/Scientistis/AboutADNI.aspx#

2 http://adni.loni.usc.edu/methods/mri-tool/mri-analysis/

3 http://adni.loni.usc.edu/methods/pet-analysis-method/pet-analysis/

4 http://adni.loni.usc.edu/data-samples/data-types/

5 http://d.rnet.co/DPABI/DPABI_V2.3_170105.zip

6 https://github.com/IvanoLauriola/MKLpy

7 https://github.com/Alzheimer1/Classification-of-Alzheimer-s-disease
