# Supplementary material for: Classification and Graphical Analysis of Alzheimer’s Disease and Its Prodromal Stage Using Multimodal Features From Structural, Diffusion, and Functional Neuroimaging Data and the APOE Genotype
Source: Front Aging Neurosci. 2020 Jul 30;12:238. doi: 10.3389/fnagi.2020.00238 (PMC7406801; doi:10.3389/fnagi.2020.00238)
Supplement: Supplementary file 1 [file Table_1.DOCX]

Subject IDs in ADNI dataset (129 subjects)

| No. | Subject ID | Clinical stage | No. | Subject ID | Clinical stage | No. | Subject ID | Clinical stage |
| --- | --- | --- | --- | --- | --- | --- | --- | --- |
| **1** | 003_S_6264 | AD | **2** | 003_S_6833 | AD | **3** | 006_S_6689 | AD |
| **4** | 011_S_4827 | AD | **5** | 011_S_6303 | AD | **6** | 013_S_6768 | AD |
| **7** | 016_S_6708 | AD | **8** | 016_S_6839 | AD | **9** | 022_S_6013 | AD |
| **10** | 022_S_6796 | AD | **11** | 023_S_6661 | AD | **12** | 027_S_6648 | AD |
| **13** | 027_S_6733 | AD | **14** | 027_S_6849 | AD | **15** | 032_S_6600 | AD |
| **16** | 032_S_6602 | AD | **17** | 033_S_6705 | AD | **18** | 035_S_6660 | AD |
| **19** | 036_S_6179 | AD | **20** | 036_S_6231 | AD | **21** | 037_S_6216 | AD |
| **22** | 082_S_6690 | AD | **23** | 094_S_6736 | AD | **24** | 098_S_6601 | AD |
| **25** | 098_S_6655 | AD | **26** | 098_S_6658 | AD | **27** | 100_S_6713 | AD |
| **28** | 114_S_6039 | AD | **29** | 114_S_6347 | AD | **30** | 116_S_6100 | AD |
| **31** | 116_S_6543 | AD | **32** | 126_S_6683 | AD | **33** | 126_S_6721 | AD |
| **34** | 002_S_4473 | MCIs | **35** | 002_S_4799 | MCIs | **36** | 003_S_2374 | MCIs |
| **37** | 005_S_4185 | MCIs | **38** | 007_S_2394 | MCIs | **39** | 007_S_4272 | MCIs |
| **40** | 011_S_4547 | MCIs | **41** | 011_S_4893 | MCIs | **42** | 012_S_4188 | MCIs |
| **43** | 013_S_2389 | MCIs | **44** | 013_S_4268 | MCIs | **45** | 014_S_2308 | MCIs |
| **46** | 016_S_5031 | MCIs | **47** | 018_S_2133 | MCIs | **48** | 018_S_2155 | MCIs |
| **49** | 018_S_2180 | MCIs | **50** | 018_S_4868 | MCIs | **51** | 021_S_4659 | MCIs |
| **52** | 021_S_4744 | MCIs | **53** | 022_S_2263 | MCIs | **54** | 022_S_2379 | MCIs |
| **55** | 022_S_5004 | MCIs | **56** | 023_S_2068 | MCIs | **57** | 024_S_2239 | MCIs |
| **58** | 024_S_4674 | MCIs | **59** | 027_S_2219 | MCIs | **60** | 027_S_2245 | MCIs |
| **61** | 027_S_4919 | MCIs | **62** | 029_S_2395 | MCIs | **63** | 031_S_2018 | MCIs |
| **64** | 002_S_4229 | MCIc | **65** | 002_S_4654 | MCIc | **66** | 003_S_4354 | MCIc |
| **67** | 006_S_4713 | MCIc | **68** | 006_S_4960 | MCIc | **69** | 007_S_4611 | MCIc |
| **70** | 009_S_4324 | MCIc | **71** | 012_S_4094 | MCIc | **72** | 016_S_4902 | MCIc |
| **73** | 019_S_4293 | MCIc | **74** | 023_S_4115 | MCIc | **75** | 027_S_4869 | MCIc |
| **76** | 031_S_4721 | MCIc | **77** | 035_S_4114 | MCIc | **78** | 035_S_4414 | MCIc |
| **79** | 036_S_4430 | MCIc | **80** | 036_S_4538 | MCIc | **81** | 036_S_4715 | MCIc |
| **82** | 037_S_4030 | MCIc | **83** | 037_S_4214 | MCIc | **84** | 037_S_4302 | MCIc |
| **85** | 041_S_4510 | MCIc | **86** | 067_S_4767 | MCIc | **87** | 067_S_4782 | MCIc |
| **88** | 068_S_4061 | MCIc | **89** | 094_S_4630 | MCIc | **90** | 109_S_4531 | MCIc |
| **91** | 114_S_5047 | MCIc | **92** | 123_S_4170 | MCIc | **93** | 126_S_4507 | MCIc |
| **94** | 126_S_4896 | MCIc | **95** | 002_S_0413 | HC | **96** | 002_S_1261 | HC |
| **97** | 002_S_1280 | HC | **98** | 002_S_4213 | HC | **99** | 002_S_4225 | HC |
| **100** | 003_S_4119 | HC | **101** | 003_S_4288 | HC | **102** | 003_S_4350 | HC |
| **103** | 003_S_4441 | HC | **104** | 003_S_4644 | HC | **105** | 003_S_4900 | HC |
| **106** | 005_S_0602 | HC | **107** | 005_S_0610 | HC | **108** | 006_S_0498 | HC |
| **109** | 006_S_4357 | HC | **110** | 006_S_4485 | HC | **111** | 007_S_4387 | HC |
| **112** | 007_S_4488 | HC | **113** | 007_S_4516 | HC | **114** | 007_S_4620 | HC |
| **115** | 007_S_4637 | HC | **116** | 009_S_0751 | HC | **117** | 009_S_4388 | HC |
| **118** | 009_S_4612 | HC | **119** | 010_S_0419 | HC | **120** | 011_S_0021 | HC |
| **121** | 011_S_4105 | HC | **122** | 011_S_4278 | HC | **123** | 012_S_4643 | HC |
| **124** | 013_S_4580 | HC | **125** | 014_S_4401 | HC | **126** | 014_S_4576 | HC |
| **127** | 016_S_4121 | HC | **128** | 016_S_4951 | HC | **129** | 016_S_4952 | HC |
