# Supplementary material for: Classification and Graphical Analysis of Alzheimer’s Disease and Its Prodromal Stage Using Multimodal Features From Structural, Diffusion, and Functional Neuroimaging Data and the APOE Genotype
Source: Front Aging Neurosci. 2020 Jul 30;12:238. doi: 10.3389/fnagi.2020.00238 (PMC7406801; doi:10.3389/fnagi.2020.00238)
Supplement: Supplementary file 2 [file Data_Sheet_1.docx]

Supplementary Material

Classification and graphical analysis of Alzheimer’s disease and its prodromal stage using multimodal features from structural, diffusion, and functional neuroimaging data and the APOE genotype

Yubraj Gupta, Ji-In Kim, and Goo-Rak Kwon*, and for the Alzheimer’s Disease Neuroimaging Initiative¶

Department of Information and Communication Engineering, Chosun University, 309 Pilmun-Daero, Dong-Gu, Gwangju 61452, Republic of Korea

* **Correspondence**: Goo-Rak Kwon (<grkwon@chosun.ac.kr>), Tel: +82-062-230-7707

**Table S1.** Cluster information (AD vs. HC) using sMRI images

| Cluster | Number of voxels | Peak MNI coordinates | | | Peak MNI coordinate region | Peak intensity  (T-Value) |
| --- | --- | --- | --- | --- | --- | --- |
|  |  | X | Y | Z |  |  |
| Cluster 1 | 1108 | -21 | -26 | 8 | Thalamus_L | 4.76 |
| Cluster 2 | 217 | 30 | 44 | 11 | Frontal_Sup_R | 3.81 |
| Cluster 3 | 54 | -21 | 74 | 3 | Precentral_L | 3.6 |
| Cluster 4 | 592 | -47 | 5 | -30 | Temporal_Mid_L | 3.52 |
| Cluster 5 | 23 | -9 | 68 | 32 | Pallidum_R | 3.29 |
| Cluster 6 | 175 | 18 | -20 | 2 | Thalamus_R | 3.14 |
| Cluster 7 | 105 | -62 | -8 | -9 | Pallidum_L | 2.92 |
| Cluster 8 | 19 | 53 | 5 | -27 | Temporal_Mid_R | 2.54 |

Table S1 shows the main affected areas observed in the AD vs. HC group analyzed using sMRI images and the obtained voxel clusters including their detailed information. The minimum cluster size was kept at 19 voxels because we found a huge number of changes in their GM brain regions while operating two-sample *t-tests* in this set. Each cluster contains more than 19 adjacent voxels that display the significant differences in those diffusion factors. The selected significant voxels are shown with their *T-values*. Here, ROI was defined by including the suprathreshold intensity voxels. Furthermore, the integral of suprathreshold intensities inside a cluster naturally joins both signal extent and intensity. Hence, Table S1 shows that for an AD vs. HC set, positive suprathreshold intensities have revealed the significantly affected area by overlapping AD patient images and HC subject images for extracting differences in the GM area as a cluster, which was presented by their *T-value*. Furthermore, Table S1 shows that the left hemisphere of the thalamus region (1108 voxels) had a significantly smaller GM volume when comparing the AD group with the HC set. Their peak intensity (*T-value*) score was 4.76.

**Table S2.** Cluster information (AD vs. HC) using FDG-PET imaging

| Cluster | Number of voxels | Peak MNI coordinates | | | Peak MNI coordinate region | Peak intensity  (T-Value) |
| --- | --- | --- | --- | --- | --- | --- |
|  |  | X | Y | Z |  |  |
| Cluster 1 | 66 | -20 | -92 | -6 | Occipital_Inf_L | 4.29 |
| Cluster 2 | 887 | 10 | -66 | 8 | Calcarine_R | 4.24 |
| Cluster 3 | 59 | -52 | -30 | -6 | Temporal_Mid_L | 3.98 |
| Cluster 4 | 19 | 24 | -22 | -24 | ParaHippocampal_R | 3.79 |
| Cluster 5 | 27 | 56 | -10 | -24 | Temporal_Mid_R | 3.68 |
| Cluster 6 | 21 | 46 | 30 | 14 | Frontal_Inf_Tri_R | 3.67 |
| Cluster 7 | 8 | -20 | -18 | -26 | ParaHippocampal_L | 3.58 |
| Cluster 8 | 10 | 64 | -14 | 8 | Temporal_Sup_R | 3.55 |
| Cluster 9 | 7 | -40 | 36 | 8 | Frontal_Inf_Tri_L | 3.48 |
| Cluster 10 | 8 | -42 | -68 | -10 | Frontal_Sup_L | 3.4 |

Table S2 shows the main influenced area distributed in the AD vs. HC group via FDG-PET imaging and the obtained voxel clusters with detailed information. The minimum cluster size, in this case, was kept at seven voxels because we found a huge number of changes in their GM brain region while operating two-sample *t-tests* in this set. Here, each cluster contains more than seven adjacent voxels that displayed a significant difference in those diffusion factors. The selected significant voxels are shown with their *T-values*. Table S2 shows that for AD vs. HC set, positive suprathreshold intensities revealed the significantly affected area via overlapping AD patient images with HC subject images for extracting the GM difference area as a cluster, which was presented by their *T-value*. Furthermore, Table S2 shows that the left hemisphere of the Occipital_Inf_L region (66 voxels) had a significantly reduced GM volume when comparing an AD group with the HC set. Their peak intensity (*T-value*) score was 4.29.

**Table S3.** Cluster information (AD vs. HC) using AV45-PET imaging

| Cluster | Number of voxels | Peak MNI coordinates | | | Peak MNI coordinate region | Peak intensity  (T-Value) |
| --- | --- | --- | --- | --- | --- | --- |
|  |  | X | Y | Z |  |  |
| Cluster 1 | 1004 | 40 | -38 | -16 | Fusiform_R | 3.94 |
| Cluster 2 | 293 | -28 | -6 | -22 | Hippocampus_L | 3.52 |
| Cluster 3 | 17 | 42 | -4 | -38 | Temporal_Inf_R | 3.37 |
| Cluster 4 | 149 | 28 | 4 | -18 | Amygdala_R | 3.36 |
| Cluster 5 | 40 | 42 | 6 | 16 | Lingual_R | 3.02 |
| Cluster 6 | 87 | 20 | -60 | -4 | Temporal_Sup_R | 2.92 |
| Cluster 7 | 28 | -28 | -60 | 32 | Occipital_Mid_L | 2.7 |
| Cluster 8 | 14 | -34 | -36 | -18 | Temporal_Mid_R | 2.58 |

Table S3 shows the main affected area dispersed in the AD vs. HC group via AV45-PET imaging and the obtained voxel clusters including their detailed information. The minimum cluster size, in this case, was kept at 14 voxels because we found a huge number of changes in their GM brain regions via two-sample *t-tests* in this set. Here, each cluster contained more than 14 adjacent voxels that displayed a significant difference in those diffusion factors. The selected significant voxels are shown with their *T-values*. Moreover, Table S3 shows that the right hemisphere of the fusiform (1004 voxels) region had a significantly reduced GM volume when comparing the AD group with the HC group. Their peak intensity (*T-value*) score was 3.94. Figure 7 illustrates the selected regions for the AD vs. HC group comparison.

**Table S4.** Cluster information (AD vs. HC) using rs-fMRI imaging

| Cluster | Number of voxels | Peak MNI coordinates | | | Peak MNI coordinate region | Peak intensity  (T-Value) |
| --- | --- | --- | --- | --- | --- | --- |
|  |  | X | Y | Z |  |  |
| ALFF | | | | | | |
| Cluster 1 | 1192 | 30 | -42 | 21 | Temporal_Inf_R | 5.39 |
| Cluster 2 | 397 | 6 | -84 | -42 | Cerebelum_Crus2_R | 5.28 |
| Cluster 3 | 294 | -6 | -45 | -42 | Cerebelum_9_L | 5.07 |
| Cluster 4 | 300 | -36 | -42 | 6 | Fusiform_L | 4.79 |
| Cluster 5 | 80 | -6 | 0 | -9 | Hippocampus_L | 4.31 |
| Cluster 6 | 45 | 24 | -102 | -12 | Occipital_Inf_R | 4.5 |
| Cluster 7 | 30 | -60 | -39 | -21 | Temporal_Inf_L | 4.04 |
| FALFF | | | | | | |
| Cluster 1 | 157 | 48 | -75 | 21 | Temporal_Mid_R | 5.19 |
| Cluster 2 | 36 | -12 | -75 | -27 | Cerebelum_Crus1_L | 4.86 |
| Cluster 3 | 30 | -42 | -78 | 24 | Occipital_Mid_L | 4.43 |
| Cluster 4 | 44 | 39 | -60 | 42 | Angular_R | 4.25 |
| Cluster 5 | 24 | 9 | -57 | 30 | Precuneus_R | 4.08 |
| Cluster 6 | 17 | -6 | -93 | 12 | Calcarine_L | 3.85 |
| Cluster 7 | 15 | -27 | -69 | 30 | Temporal_Inf_L | 4.37 |
| REHO | | | | | | |
| Cluster 1 | 60 | 15 | -27 | 30 | Temporal_Inf_R | 5.96 |
| Cluster 2 | 27 | 9 | -36 | 15 | Fusiform_L | 5 |
| Cluster 3 | 52 | 3 | -93 | -21 | Cerebelum_Crus2_L | 4.99 |
| Cluster 4 | 35 | 48 | -6 | -24 | Occipital_Mid_L | 4.7 |
| Cluster 5 | 39 | -30 | 0 | -9 | Temporal_Mid_L | 4.59 |
| Cluster 6 | 17 | 30 | -45 | -48 | Cerebelum_8_R | 4.48 |
| Cluster 7 | 12 | 36 | -54 | -6 | Cingulate Gyrus | 3.96 |
| Cluster 8 | 11 | 42 | -78 | -33 | Cerebelum_Crus1_R | 4.79 |

Table S4 shows the main affected areas in the AD vs. HC group analyzed via rs-fMRI and the obtained voxel clusters including their detailed information using three different whole-brain feature maps (ALFF, fALFF, REHO). The minimum cluster size for all cases was kept at 11 voxels because we found a huge number of changes in their GM brain regions while operating two-sample *t-tests* in this set. Each cluster contained more than 11 adjacent voxels that displayed significant differences in those diffusion factors. The selected significant voxels are shown with their *T-values*. Table S4 shows that for the AD vs. HC set, positive suprathreshold intensities revealed the major affected regions by overlapping AD patient images over HC subject images for extracting GM difference areas as a cluster, which was presented by their *T-value* in every feature map. Furthermore, Table S4 shows that in the case of ALFF, the right hemisphere of the Temporal_Inf_R region (1192 voxels) had a significantly reduced GM volume when comparing the AD group with the HC group. Their peak intensity (*T-value*) score was 5.39. Likewise, in the case of fALFF, the right hemisphere of the Temporal_Mid_R region (157 voxels) displayed a significantly reduced GM volume. Their peak intensity value was 5.19. Moreover, in the REHO feature map, the right hemisphere of the Temporal_Inf_R region (60 voxels) displayed a significantly reduced GM volume compared with other clusters. Its obtained peak intensity (*T-value*) value was 5.96. It is interesting to note that, even though the number of voxels of cluster 1 of ALFF and fALFF was higher than REHO, the obtained peak intensity value was higher (5.96) in REHO compared to other feature maps.

**Table S5.** Cluster information (AD vs. HC) using DTI-FA imaging

| Cluster | Number of voxels | Peak MNI coordinates | | | Peak MNI coordinate region | Peak intensity  (T-Value) |
| --- | --- | --- | --- | --- | --- | --- |
|  |  | X | Y | Z |  |  |
| Cluster 1 | 124 | -36.3 | -50 | -9.42 | Sagittal stratum L | 4.38 |
| Cluster 2 | 2469 | 27.9 | -17.2 | 28.3 | Superior corona radiata R | 4.22 |
| Cluster 3 | 3722 | -26.3 | -10.8 | 20.6 | Superior corona radiata L | 4.09 |
| Cluster 4 | 425 | 39.3 | -44.7 | -5.47 | Sagittal stratum R | 3.16 |
| Cluster 5 | 226 | -11.4 | -5.6 | -6.42 | Cerebral peduncle | 3.14 |
| Cluster 6 | 176 | 5.54 | 32 | -24.1 | Superior cerebellar peduncle | 3.11 |
| Cluster 7 | 100 | 17.4 | -32.1 | -34.2 | Middle cerebellar peduncle | 3.46 |
| Cluster 8 | 94 | 28.9 | 12.4 | 7.64 | External capsule | 2.31 |
| Cluster 9 | 191 | -38.1 | -0.82 | 24.2 | Superior longitudinal fasciculus | 2.76 |
| Cluster 10 | 78 | -10.5 | -24.5 | 32 | Cingulum gyrus L | 2.57 |
| Cluster 11 | 31 | 4.04 | -32.5 | -31.9 | Pontine crossing tract (MCP) | 2.39 |

For the DTI images, we have only extracted FA maps for all subjects using the FMRIB diffusion toolbox. These extracted FA images were further processed and analyzed using the TBSS tool available in FSL. Table S5 shows the main affected areas observed in the AD vs. HC group analyzed via DTI-FA imaging and the obtained voxel clusters including their detailed information. The minimum cluster size kept at 31 voxels because we found a huge number of changes in their WM brain regions while conducting two-sample *t-tests* in this set. Each cluster contained more than 31 adjacent voxels that showed the major variation in those diffusion factors. The selected significant voxels are displayed with their *T-values*. Furthermore, Table S5 shows that the left hemisphere of the sagittal stratum region (124 voxels) displayed a significantly reduced WM volume when comparing the AD group with the HC group. Their peak intensity (*T-value*) score was 4.38. Figure 8 illustrates the selected regions for the AD vs. HC group comparison.

**Table S6.** Cluster information (MCIs vs. MCIc) using sMRI imaging

| Cluster | Number of voxels | Peak MNI coordinates | | | Peak MNI coordinate region | Peak intensity  (T-Value) |
| --- | --- | --- | --- | --- | --- | --- |
|  |  | X | Y | Z |  |  |
| Cluster 1 | 1329 | 8 | -72 | -60 | Precuneus_L | 5.03 |
| Cluster 2 | 471 | 29 | -27 | 80 | Parietal_Sup_L | 3.91 |
| Cluster 3 | 342 | 8 | -92 | 39 | Cingulum_Mid_L | 4.13 |
| Cluster 4 | 557 | 17 | 20 | 42 | Frontal_Mid_R | 3.93 |
| Cluster 5 | 223 | 39 | 35 | 26 | Frontal_Inf_Tri_R | 3.39 |
| Cluster 6 | 579 | 50 | 6 | 11 | Rolandic_Oper_R | 3.35 |
| Cluster 7 | 287 | -18 | -80 | -11 | Lingual_L | 3.33 |
| Cluster 8 | 25 | 6 | -48 | -38 | Cerebelum_9_R | 2.6 |
| Cluster 9 | 326 | 44 | -24 | 0 | Temporal_Sup_R | 2.88 |

Table S6 shows the main affected areas in the MCIs vs. MCIc groups obtained via sMRI imaging and the achieved voxel clusters with detailed information. The minimum cluster size, in this case, was kept at 25 voxels because we found a huge number of changes in their GM brain regions while operating two-sample *t-tests* in this set. Each cluster contained more than 25 adjacent voxels that showed significant differences in those diffusion factors. The selected major voxels are shown with their *T-values*. Here, ROIs were defined by including the suprathreshold intensity voxels. Furthermore, the integral of suprathreshold intensities inside a cluster naturally joins both signal intensity and signal extent. Hence, Table S6 shows that for MCIs vs. MCIc group, positive suprathreshold intensities revealed the significantly affected area by overlapping MCIs subject images over MCIc subject images for extracting the GM alteration region as a cluster, which was presented by their *T-value*. Moreover, Table S6 shows that the left hemisphere of the precuneus region (1329 voxels) displayed a significantly reduced GM volume when comparing the MCIs group with the MCIc group. Their peak intensity (*T-value*) score was 5.03.

**Table S7.** Cluster information (MCIs vs. MCIc) using FDG-PET imaging

| Cluster | Number of voxels | Peak MNI coordinates | | | Peak MNI coordinate region | Peak intensity  (T-Value) |
| --- | --- | --- | --- | --- | --- | --- |
|  |  | X | Y | Z |  |  |
| Cluster 1 | 3369 | 0 | -60 | -40 | Vermis_9 | 4.31 |
| Cluster 2 | 33 | -36 | -56 | -46 | Cerebelum_8_L | 3.93 |
| Cluster 3 | 855 | -46 | -68 | -8 | Temporal_Inf_L | 3.86 |
| Cluster 4 | 20 | -36 | -78 | 16 | Occipital_Mid_L | 3.6 |
| Cluster 5 | 470 | -4 | 50 | 26 | Frontal_Sup_Medial_L | 3.44 |
| Cluster 6 | 10 | -42 | -76 | 4 | Thalamus_R | 3.39 |
| Cluster 7 | 50 | -4 | 36 | 34 | Precuneus_L | 3.29 |
| Cluster 8 | 14 | 8 | 42 | 28 | Cingulum_Ant_R | 2.65 |
| Cluster 9 | 7 | 54 | -20 | 42 | Postcentral_R | 2.58 |

Table S7 shows the main affected area in the MCIs vs. MCIc group analyzed via FDG-PET imaging and the obtained voxel clusters with detailed information. The minimum cluster size, in this case, was kept at seven voxels because we found a huge number of changes in their GM brain regions while conducting two-sample *t-tests* in this set. Each cluster contained more than 7 adjacent voxels that displayed the significant differences in those diffusion factors. The selected significant voxels are shown with their *T-values*. Table S7 also shows that the vermis region (3369 voxels) displayed a significantly reduced GM volume when comparing the MCIs group with the MCIc group. Their peak intensity (*T-value*) value was 4.31.

**Table S8.** Cluster information (MCIs vs. MCIc) using AV45-PET imaging

| Cluster | Number of voxels | Peak MNI coordinates | | | Peak MNI coordinate region | Peak intensity  (T-Value) |
| --- | --- | --- | --- | --- | --- | --- |
|  |  | X | Y | Z |  |  |
| Cluster 1 | 1007 | -14 | 36 | 22 | Frontal_Sup_Medial_L | 3.41 |
| Cluster 2 | 29 | -4 | 2 | 34 | Cingulum_Mid_L | 3.3 |
| Cluster 3 | 143 | -2 | -36 | 22 | Precuneus_R | 3.29 |
| Cluster 4 | 305 | -40 | -46 | -6 | Cerebelum_9_L | 3.1 |
| Cluster 5 | 111 | 14 | 42 | -10 | Frontal_Med_Orb_R | 3.09 |
| Cluster 6 | 94 | 46 | -8 | 32 | Precentral_R | 3.01 |
| Cluster 7 | 386 | 36 | 10 | 26 | Lingual_L | 2.98 |
| Cluster 8 | 186 | -14 | 28 | -6 | Precentral_L | 2.96 |
| Cluster 9 | 29 | -46 | -6 | 28 | Fusiform_R | 2.86 |

Table S8 shows the main affected area in the MCIs vs. MCIc group obtained via AV45-PET imaging and the achieved voxel clusters including their detailed information. The minimum cluster size, in this case, was kept at 29 voxels because we found a huge number of changes in their GM brain regions while operating two-sample *t-tests* in this set. Each cluster contained more than 29 adjacent voxels that displayed significant differences in those diffusion factors. The selected significant voxels are shown with their *T-values*. Table S8 also shows that the left hemisphere of the Frontal_Sup_Medial region (1007 voxels) displayed a significantly reduced GM volume when comparing the MCIs group with the MCIc group. Their peak intensity (*T-value*) value was 3.41. Figure 7 illustrates the selected regions for the MCIs vs. MCIc group comparison.

**Table S9.** Cluster information (MCIc vs. MCIc) using rs-fMRI imaging

| Cluster | Number of voxels | | Peak MNI coordinates | | | Peak MNI coordinate region | Peak intensity  (T-Value) | |
| --- | --- | --- | --- | --- | --- | --- | --- | --- |
|  |  |  | X | Y | Z |  |  |  |
| ALFF | | | | | | | | |
| Cluster 1 | 16790 | | -15 | -30 | -21 | Pallidum_L | | 6.94 |
| Cluster 2 | 185 | | 6 | -45 | -57 | Cerebelum_9_R | | 5.54 |
| Cluster 3 | 68 | | 45 | -72 | -21 | Parietal_Inf_R | | 4.45 |
| Cluster 4 | 27 | | -54 | 9 | -30 | Temporal_Pole_Mid_L | | 4.68 |
| Cluster 5 | 25 | | -24 | -72 | -27 | Cerebelum_Crus1_L | | 4.31 |
| Cluster 6 | 22 | | 51 | -57 | 54 | Precuneus_L | | 4.91 |
| Cluster 7 | 16 | | 3 | -87 | 39 | ParaHippocampal_R | | 5.18 |
| FALFF | | | | | | | | |
| Cluster 1 | 4228 | | 12 | -66 | -3 | Lingual_R | 6.77 | |
| Cluster 2 | 46 | | 9 | -12 | 3 | Thalamus_R | 5.48 | |
| Cluster 3 | 43 | | 12 | 57 | -3 | Frontal_Med_Orb_R | 4.21 | |
| Cluster 4 | 40 | | 33 | 0 | 48 | Precentral_R | 5.25 | |
| Cluster 5 | 37 | | -24 | 36 | 39 | Frontal_Mid_L | 4.31 | |
| Cluster 6 | 29 | | -12 | -15 | 0 | Thalamus_L | 5.06 | |
| Cluster 7 | 15 | | 39 | -9 | 42 | Fusiform_L | 4.89 | |
| Cluster 8 | 25 | | 39 | -60 | 12 | Temporal_Mid_R | 4.58 | |
| Cluster 9 | 6 | | 42 | -30 | 30 | Precuneus_L | 4.82 | |
| REHO | | | | | | | | |
| Cluster 1 | | 511 | -42 | 57 | 12 | Frontal_Mid_L | 10.57 | |
| Cluster 2 | | 209 | 27 | 66 | 24 | Occipital_Mid_R | 8.99 | |
| Cluster 3 | | 250 | 0 | -66 | 30 | Cuneus_L | 7.91 | |
| Cluster 4 | | 234 | 27 | -87 | -51 | Cerebelum_6_R | 6.53 | |
| Cluster 5 | | 97 | 3 | -36 | 57 | ParaHippocampal_R | 6.35 | |
| Cluster 6 | | 234 | 27 | -87 | -51 | Cerebelum_6_R | 6.53 | |
| Cluster 7 | | 115 | 3 | -90 | -39 | Precuneus_L | 6.03 | |
| Cluster 8 | | 167 | 3 | -69 | 24 | Temporal_Inf_L | 5.67 | |
| Cluster 9 | | 41 | 3 | -9 | -48 | Vermis_4_5 | 5.46 | |
| Cluster 10 | | 216 | 3 | -27 | 15 | Thalamus_L | 5.26 | |
| Cluster 11 | | 130 | 3 | -36 | 57 | Paracentral_Lobule_L | 5 | |
| Cluster 12 | | 16 | 3 | -6 | -9 | Temporal_Sup_L | 4.78 | |

Table S9 shows the main affected areas in the MCIs vs. MCIc group obtained via rs-fMRI imaging and the obtained voxel clusters including their detailed information using three different whole-brain feature maps (ALFF, fALFF, REHO). The minimum cluster size for all cases was kept at six voxels because we found a huge number of changes in their GM brain regions while conducting two-sample *t-tests* in this set. Each cluster contained more than six adjacent voxels that displayed a significant difference in those diffusion factors. The selected significant voxels are shown with their *T-values*. Table S9 shows that for MCIs vs. MCIc group, positive suprathreshold intensities revealed the significantly affected areas by overlapping MCIs subject images over MCIc subject images for removing GM different regions as a group, which was represented by their *T-value* in each feature map. Table S9 also shows that, in the case of ALFF, the left hemisphere of the pallidum region (16790 voxels) displayed a significantly reduced GM volume when comparing the MCIs group with the MCIc group. Their peak intensity (*T-value*) score was 6.94. Likewise, in the case of fALFF, the right hemisphere of the lingual region (4228 voxels) had a significantly reduced GM volume. Their peak intensity value was 6.77. In the REHO feature map, the left hemisphere of the Frontal_Mid region (511 voxels) had a significantly reduced GM volume compared with other clusters. Its obtained peak intensity (*T-value*) value was 10.57. It is interesting to note that, even though the number of voxels of cluster 1 of ALFF and fALFF was higher than of REHO, the obtained peak intensity value was higher in REHO (10.57) compared to other feature maps.

**Table S10.** Cluster information (MCIs vs. MCIc) using DTI-FA imaging

| Cluster | Number of voxels | Peak MNI coordinates | | | Peak MNI coordinate region | Peak intensity  (T-Value) |
| --- | --- | --- | --- | --- | --- | --- |
|  |  | X | Y | Z |  |  |
| Cluster 1 | 431 | 26.6 | 14.7 | -9.04 | External capsule R | 4.05 |
| Cluster 2 | 548 | -20.7 | 43.8 | -0.501 | Cingulum (hippocampus) L | 3.94 |
| Cluster 3 | 157 | 46.3 | 3.09 | 18.4 | Cingulum (cingulate gyrus) L | 3.91 |
| Cluster 4 | 349 | -44.7 | -46.4 | 15.3 | Anterior limb-internal capsule L | 3.47 |
| Cluster 5 | 423 | -26.7 | -63.6 | -0.788 | Body of corpus callosum | 3.35 |
| Cluster 6 | 454 | 38.5 | -50.6 | 30.9 | Medial lemniscus R | 3.31 |
| Cluster 7 | 831 | -27.5 | -66.2 | 26.1 | Superior longitudinal fasciculus L | 3.26 |
| Cluster 8 | 447 | 3.61 | -33.3 | -25.7 | Cerebral peduncle R | 3.16 |
| Cluster 9 | 496 | 24.7 | 35.8 | -2.81 | Anterior corona radiata R | 3.01 |
| Cluster 10 | 106 | -29.3 | 9.39 | 6.54 | External capsule L | 2.96 |
| Cluster 11 | 234 | -13 | -7.45 | -11.3 | Middle cerebellar peduncle | 2.91 |
| Cluster 12 | 299 | -48.3 | -24.5 | 1.04 | Superior corona radiata L | 2.77 |

For the DTI imaging, we only extracted FA maps for all subjects using the FMRIB diffusion toolbox. These extracted FA images were further processed and analyzed using the TBSS tool available in FSL. Table S10 shows the main affected areas in the MCIs vs. MCIc group obtained via DTI-FA imaging and the achieved voxel clusters including their detailed information. The minimum cluster size, in this case, was kept at 106 voxels because we found a huge number of changes in their WM brain regions while conducting two-sample *t-tests* in this set. Each cluster contained more than 106 adjacent voxels that displayed significant differences in those diffusion parameters. The selected significantly different voxels are displayed with their *T-values*. Table S10 also shows that the right hemisphere of the external-capsule region (431 voxels) had a significant loss in WM volume when comparing the MCIs group with the MCIc group. Their peak intensity (*T-value*) value was 4.05. Figure 8 illustrates the selected regions for the MCIs vs. MCIc group comparison.

**Table S11.** Cluster information (AD vs. MCIc) using sMRI imaging

| Cluster | Number of voxels | Peak MNI coordinates | | | Peak MNI coordinate region | Peak intensity  (T-Value) |
| --- | --- | --- | --- | --- | --- | --- |
|  |  | X | Y | Z |  |  |
| Cluster 1 | 241 | 60 | 26 | 12 | Frontal_Inf_Tri_R | 3.83 |
| Cluster 2 | 171 | 62 | 11 | 6 | Rolandic_Oper_R | 3.49 |
| Cluster 3 | 110 | 60 | 9 | 15 | Precentral_R | 3.12 |
| Cluster 4 | 171 | 51 | 11 | 9 | Frontal_Inf_Oper_R | 3.05 |
| Cluster 5 | 15 | -56 | 14 | 3 | Frontal_Inf_Oper_L | 2.55 |
| Cluster 6 | 9 | -54 | 15 | 39 | Middle Frontal Gyrus | 2.40 |
| Cluster 7 | 64 | 33 | 33 | 3 | Inferior Frontal Gyrus | 2.21 |

Table S11 shows the main affected areas in the AD vs. MCIc groups obtained via sMRI imaging and the achieved voxel clusters with detailed information. The minimum cluster size, in this case, was kept at 5 voxels because we found a huge number of changes in their GM brain regions while operating two-sample *t-tests* in this set. The selected major voxels are shown with their *T-values*. Table S11 shows that for theAD vs. MCIc group, positive suprathreshold intensities revealed the significantly affected area by overlapping AD subject images over MCIc subject images for extracting the GM alteration region as a cluster, which was presented by their *T-value*. Moreover, Table S11 shows that the right hemisphere of the frontal-inf-tri region (241 voxels) displayed a significantly reduced GM volume when comparing the AD group with the MCIc group. Their peak intensity (*T-value*) score was 3.83.

**Table S12.** Cluster information (AD vs. MCIc) using FDG-PET imaging

| Cluster | Number of voxels | Peak MNI coordinates | | | Peak MNI coordinate region | Peak intensity  (T-Value) |
| --- | --- | --- | --- | --- | --- | --- |
|  |  | X | Y | Z |  |  |
| Cluster 1 | 8919 | -14 | 4 | 86 | Frontal_Inf_Tri_L | 4.67 |
| Cluster 2 | 236 | 14 | 12 | 82 | Middle Occipital Gyrus | 4.25 |
| Cluster 3 | 26 | -30 | 30 | 64 | Superior Temporal Gyrus | 3.64 |
| Cluster 4 | 12 | 38 | -26 | 80 | Occipital Lobe | 3.41 |
| Cluster 5 | 78 | -52 | -70 | -50 | Inferior Semi-Lunar Lobule | 2.94 |
| Cluster 6 | 5 | -32 | 18 | 70 | Middle Temporal Gyrus | 2.53 |

Table S12 shows the main affected area in the AD vs. MCIc group analyzed via FDG-PET imaging and the obtained voxel clusters with detailed information. The minimum cluster size, in this case, was kept at 5 voxels because we found a huge number of changes in their GM brain regions while conducting two-sample *t-tests* in this set. Each cluster contained more than 5 adjacent voxels that displayed the significant differences in those diffusion factors. The selected significant voxels are shown with their *T-values*. Table S12 also shows that the frontal-inf-tri region (8919 voxels) displayed a significantly reduced GM volume when comparing the AD group with the MCIc group. Their peak intensity (*T-value*) value was 4.67.

**Table S13.** Cluster information (AD vs. MCIc) using AV45-PET imaging

| Cluster | Number of voxels | Peak MNI coordinates | | | Peak MNI coordinate region | Peak intensity  (T-Value) |
| --- | --- | --- | --- | --- | --- | --- |
|  |  | X | Y | Z |  |  |
| Cluster 1 | 479 | 62 | -8 | -8 | Temporal_Sup_R | 5.13 |
| Cluster 2 | 179 | -44 | 38 | 16 | Frontal_Mid_L | 4.39 |
| Cluster 3 | 159 | -60 | -34 | -8 | Temporal_Mid_L | 4.27 |
| Cluster 4 | 95 | -64 | -34 | -8 | Occipital-Mid-L | 4.26 |
| Cluster 5 | 28 | 64 | -24 | 6 | Frontal-Inf-Tri-R | 3.82 |

Table S13 shows the main affected area in the AD vs. MCIc group obtained via AV45-PET imaging and the achieved voxel clusters including their detailed information. The minimum cluster size, in this case, was kept at 20 voxels because we found a huge number of changes in their GM brain regions while operating two-sample *t-tests* in this set. Each cluster contained more than 20 adjacent voxels that displayed significant differences in those diffusion factors. The selected significant voxels are shown with their *T-values*. Table S13 also shows that the right hemisphere of the Temporal-Sup region (479 voxels) displayed a significantly reduced GM volume when comparing the AD group with the MCIc group. Their peak intensity (*T-value*) value was 5.13.

**Table S14.** Cluster information (AD vs. MCIc) using rs-fMRI imaging

| Cluster | Number of voxels | | Peak MNI coordinates | | | Peak MNI coordinate region | Peak intensity  (T-Value) | |
| --- | --- | --- | --- | --- | --- | --- | --- | --- |
|  |  |  | X | Y | Z |  |  |  |
| ALFF | | | | | | | | |
| Cluster 1 | 1132 | | 33 | 24 | -9 | Frontal_Inf_Orb_R | | 5.05 |
| Cluster 2 | 783 | | -21 | -24 | -24 | ParaHippocampal_L | | 4.78 |
| Cluster 3 | 139 | | -6 | -27 | 9 | Putamen_L | | 4.75 |
| Cluster 4 | 114 | | 24 | -12 | -6 | Hippocampus_R | | 4.19 |
| Cluster 5 | 11 | | 45 | 6 | -12 | Insula_R | | 4.17 |
| Cluster 6 | 24 | | 0 | -27 | -24 | Lingual_L | | 3.99 |
| Cluster 7 | 8 | | -21 | -36 | -21 | Cerebelum_4_5_L | | 3.87 |
| FALFF | | | | | | | | |
| Cluster 1 | 1118 | | 18 | -9 | 6 | Thalamus_R | 5.27 | |
| Cluster 2 | 201 | | 63 | -51 | -3 | Precentral_L | 4.85 | |
| Cluster 3 | 116 | | 3 | -48 | -18 | Vermis_3 | 4.54 | |
| Cluster 4 | 75 | | -18 | -12 | 6 | Pallidum-L | 4.41 | |
| Cluster 5 | 50 | | 12 | -66 | -3 | Lingual_R | 4.12 | |
| Cluster 6 | 36 | | -54 | -63 | -36 | Cerebelum-Crus1-L | 3.96 | |
| Cluster 7 | 7 | | 0 | -75 | -3 | Lingual_L | 3.81 | |
| Cluster 8 | 5 | | 21 | -93 | -30 | Cerebelum_Crus2_R | 3.63 | |
| REHO | | | | | | | | |
| Cluster 1 | | 661 | 18 | -36 | 6 | Hippocampus_R | 6.21 | |
| Cluster 2 | | 461 | -15 | -27 | 9 | Thalamus-L | 5.13 | |
| Cluster 3 | | 282 | -21 | -3 | 3 | Pallidum_L | 4.74 | |
| Cluster 4 | | 172 | -14 | -50 | 3 | Calcarine-L | 3.11 | |
| Cluster 5 | | 82 | 5 | -60 | 3 | Precuneus_L | 3.06 | |

Table S14 shows the main affected areas in the AD vs. MCIc group obtained via rs-fMRI imaging and the obtained voxel clusters including their detailed information using three different whole-brain feature maps (ALFF, fALFF, REHO). The minimum cluster size for all cases was kept at 5 voxels because we found a huge number of changes in their GM brain regions while conducting two-sample *t-tests* in this set. Each cluster contained more than five adjacent voxels that displayed a significant difference in those diffusion factors. The selected significant voxels are shown with their *T-values*. Table S14 shows that for the AD vs. MCIc group, positive suprathreshold intensities revealed the significantly affected areas by overlapping AD subject images over MCIc subject images for removing GM different regions as a group, which was represented by their *T-value* in each feature map. Table S14 also shows that, in the case of ALFF, the right hemisphere of the frontal-inf-orb region (1132 voxels) displayed a significantly reduced GM volume when comparing the AD group with the MCIc group. Their peak intensity (*T-value*) score was 5.05. Likewise, in the case of fALFF, the right hemisphere of the thalamus region (1118 voxels) had a significantly reduced GM volume. Their peak intensity value was 5.27. In the REHO feature map, the right hemisphere of the hippocampus region (661 voxels) had a significantly reduced GM volume compared with other clusters. Its obtained peak intensity (*T-value*) value was 6.21.

**Table S15.** Cluster information (AD vs. MCIc) using DTI-FA imaging

| Cluster | Number of voxels | Peak MNI coordinates | | | Peak MNI coordinate region | Peak intensity  (T-Value) |
| --- | --- | --- | --- | --- | --- | --- |
|  |  | X | Y | Z |  |  |
| Cluster 1 | 647 | 2 | -31 | -24 | Anterior thalamic radiation R | 3.72 |
| Cluster 2 | 284 | 38 | -47 | -11 | Inferior longitudinal fasciculus R | 3.62 |
| Cluster 3 | 261 | 53 | 5 | 14 | Superior longitudinal fasciculus R | 3.60 |
| Cluster 4 | 180 | -30 | 8 | 5 | Uncinate fasciculus L | 3.52 |
| Cluster 5 | 162 | 11 | -32 | -26 | Corticospinal tract R | 3.35 |
| Cluster 6 | 73 | -37 | -46 | -13 | Inferior longitudinal fasciculus L | 3.03 |
| Cluster 7 | 91 | 33 | 30 | 19 | Uncinate fasciculus R | 2.64 |
| Cluster 8 | 77 | -24 | -55 | -36 | Middle cerebellar peduncle | 2.62 |
| Cluster 9 | 114 | -35 | -48 | 32 | Superior longitudinal fasciculus R | 2.60 |

For the DTI imaging, we only extracted FA maps for all subjects using the FMRIB diffusion toolbox. These extracted FA images were further processed and analyzed using the TBSS tool available in FSL. Table S15 shows the main affected areas in the AD vs. MCIc group obtained via DTI-FA imaging and the achieved voxel clusters including their detailed information. The minimum cluster size, in this case, was kept at 60 voxels because we found a huge number of changes in their WM brain regions while conducting two-sample *t-tests* in this set. Each cluster contained more than 60 adjacent voxels that displayed significant differences in those diffusion parameters. The selected significantly different voxels are displayed with their *T-values*. Table S15 also shows that the right hemisphere of the anterior thalamic radiation region (647 voxels) had a significant loss in WM volume when comparing the AD group with the MCIc group. Their peak intensity (*T-value*) value was 3.72.

**Table S16.** Cluster information (AD vs. MCIs) using sMRI imaging

| Cluster | Number of voxels | Peak MNI coordinates | | | Peak MNI coordinate region | Peak intensity  (T-Value) |
| --- | --- | --- | --- | --- | --- | --- |
|  |  | X | Y | Z |  |  |
| Cluster 1 | 574 | 24 | 54 | -9 | Frontal_Mid_Orb_R | 3.77 |
| Cluster 2 | 498 | 56 | -5 | -21 | Temporal_Mid_R | 3.65 |
| Cluster 3 | 134 | 29 | 33 | 27 | Frontal_Mid_R | 3.44 |
| Cluster 4 | 82 | -23 | -27 | 9 | Pulvinar | 3.12 |
| Cluster 5 | 155 | 41 | 29 | 8 | Frontal_Inf_Tri_R | 2.57 |
| Cluster 6 | 115 | -33 | 11 | 15 | Insula_L | 2.82 |
| Cluster 7 | 115 | -41 | 11 | 9 | Frontal_Inf_Oper_L | 2.61 |
| Cluster 8 | 228 | 56 | -8 | 41 | Precentral_R | 2.39 |
| Cluster 9 | 638 | -63 | -17 | 35 | Postcentral_L | 2.02 |

Table S16 shows the main affected areas in the AD vs. MCIs groups obtained via sMRI imaging and the achieved voxel clusters with detailed information. The minimum cluster size, in this case, was kept at 55 voxels because we found a huge number of changes in their GM brain regions while operating two-sample *t-tests* in this set. The selected major voxels are shown with their *T-values*. Moreover, Table S16 shows that the right hemisphere of the frontal-mid-orb region (574 voxels) displayed a significantly reduced GM volume when comparing the AD group with the MCIs group. Their peak intensity (*T-value*) score was 3.77.

**Table S17.** Cluster information (AD vs. MCIs) using FDG-PET imaging

| Cluster | Number of voxels | Peak MNI coordinates | | | Peak MNI coordinate region | Peak intensity  (T-Value) |
| --- | --- | --- | --- | --- | --- | --- |
|  |  | X | Y | Z |  |  |
| Cluster 1 | 2917 | 36 | -76 | 34 | Occipital_Mid_R | 3.79 |
| Cluster 2 | 1167 | 6 | -68 | 28 | Precuneus_R | 3.68 |
| Cluster 3 | 277 | -30 | -68 | 34 | Occipital_Mid_L | 3.37 |
| Cluster 4 | 112 | -58 | -58 | -6 | Temporal_Inf_L | 2.81 |
| Cluster 5 | 159 | 32 | 58 | 20 | Frontal_Mid_R | 2.75 |
| Cluster 6 | 66 | -32 | -26 | 56 | Precentral_L | 2.39 |
| Cluster 7 | 17 | -8 | -56 | -10 | Cerebelum_4_5_L | 2.02 |

Table S17 shows the main affected area in the AD vs. MCIs group analyzed via FDG-PET imaging and the obtained voxel clusters with detailed information. The minimum cluster size, in this case, was kept at 10 voxels because we found a huge number of changes in their GM brain regions while conducting two-sample *t-tests* in this set. Each cluster contained more than 10 adjacent voxels that displayed the significant differences in those diffusion factors. The selected significant voxels are shown with their *T-values*. Table S17 also shows that the occipital-mid region (2917 voxels) displayed a significantly reduced GM volume when comparing the AD group with the MCIs group. Their peak intensity (*T-value*) value was 3.79.

**Table S18.** Cluster information (AD vs. MCIs) using AV45-PET imaging

| Cluster | Number of voxels | Peak MNI coordinates | | | Peak MNI coordinate region | Peak intensity  (T-Value) |
| --- | --- | --- | --- | --- | --- | --- |
|  |  | X | Y | Z |  |  |
| Cluster 1 | 996 | 12 | -18 | 80 | Precentral_R | 3.84 |
| Cluster 2 | 109 | 68 | -84 | 30 | Frontal_Sup_R | 3.80 |
| Cluster 3 | 80 | -10 | -16 | 80 | Paracentral_Lobule_L | 2.76 |
| Cluster 4 | 49 | -50 | 72 | -12 | Supp_Motor_Area_L | 2.48 |
| Cluster 5 | 5 | 66 | -94 | -16 | Precentral_L | 2.38 |

Table S18 shows the main affected area in the AD vs. MCIs group obtained via AV45-PET imaging and the achieved voxel clusters including their detailed information. The minimum cluster size, in this case, was kept at 5 voxels because we found a huge number of changes in their GM brain regions while operating two-sample *t-tests* in this set. The selected significant voxels are shown with their *T-values*. Table S18 also shows that the right hemisphere of the precentral region (996 voxels) displayed a significantly reduced GM volume when comparing the AD group with the MCIs group. Their peak intensity (*T-value*) value was 3.84.

**Table S19.** Cluster information (AD vs. MCIs) using rs-fMRI imaging

| Cluster | Number of voxels | | Peak MNI coordinates | | | Peak MNI coordinate region | Peak intensity  (T-Value) | |
| --- | --- | --- | --- | --- | --- | --- | --- | --- |
|  |  |  | X | Y | Z |  |  |  |
| ALFF | | | | | | | | |
| Cluster 1 | 208 | | -33 | -48 | -9 | Fusiform_L | | 4.89 |
| Cluster 2 | 186 | | 36 | -57 | 0 | Fusiform_R | | 4.69 |
| Cluster 3 | 107 | | -60 | -54 | -18 | Temporal_Inf_L | | 4.42 |
| Cluster 4 | 68 | | 54 | -51 | -30 | Cerebelum_Crus1_R | | 3.30 |
| Cluster 5 | 37 | | 45 | -9 | -24 | Hippocampus_R | | 3.05 |
| Cluster 6 | 6 | | 51 | -36 | 21 | Temporal_Sup_R | | 2.74 |
| FALFF | | | | | | | | |
| Cluster 1 | 114 | | -30 | -81 | 21 | Occipital_Mid_L | 4.74 | |
| Cluster 2 | 55 | | 24 | -66 | 30 | Precuneus_L | 4.65 | |
| Cluster 3 | 37 | | -6 | -60 | 18 | Cuneus_L | 4.39 | |
| Cluster 4 | 19 | | 21 | -60 | 18 | Calcarine_R | 3.22 | |
| Cluster 5 | 14 | | 36 | -78 | 45 | Occipital_Sup_R | 3.02 | |
| Cluster 6 | 5 | | -21 | -45 | -9 | ParaHippocampal_L | 2.77 | |
| REHO | | | | | | | | |
| Cluster 1 | | 166 | -6 | -39 | 15 | Cingulum_Post_L | 5.67 | |
| Cluster 2 | | 38 | 27 | 15 | -9 | Frontal_Inf_Orb_R | 4.50 | |
| Cluster 3 | | 22 | 9 | 18 | 6 | Caudate_R | 4.47 | |
| Cluster 4 | | 17 | 15 | -36 | 3 | Hippocampus_R | 3.74 | |
| Cluster 5 | | 7 | -3 | -12 | 12 | Thalamus_L | 3.14 | |
| Cluster 6 | | 6 | 48 | -3 | -45 | Temporal_Inf_R | 2.24 | |

Table S19 shows the main affected areas in the AD vs. MCIs group obtained via rs-fMRI imaging and the obtained voxel clusters including their detailed information using three different whole-brain feature maps (ALFF, fALFF, REHO). The minimum cluster size for all cases was kept at 5 voxels because we found a huge number of changes in their GM brain regions while conducting two-sample *t-tests* in this set. Each cluster contained more than 5 adjacent voxels that displayed a significant difference in those diffusion factors. The selected significant voxels are shown with their *T-values*. Table S19 shows that, in the case of ALFF, the left hemisphere of the fusiform region (208 voxels) displayed a significantly reduced GM volume when comparing the AD group with the MCIs group. Their peak intensity (*T-value*) score was 4.89. Likewise, in the case of fALFF, the left hemisphere of the occipital-mid region (114 voxels) had a significantly reduced GM volume. Their peak intensity value was 4.74. In the REHO feature map, the left hemisphere of the cingulum-post region (166 voxels) had a significantly reduced GM volume compared with other clusters. Its obtained peak intensity (*T-value*) value was 5.67.

**Table S20.** Cluster information (AD vs. MCIs) using DTI-FA imaging

| Cluster | Number of voxels | Peak MNI coordinates | | | Peak MNI coordinate region | Peak intensity  (T-Value) |
| --- | --- | --- | --- | --- | --- | --- |
|  |  | X | Y | Z |  |  |
| Cluster 1 | 2661 | 21 | -27 | 30 | Posterior corona radiata R | 4.47 |
| Cluster 2 | 1970 | -29 | -25 | 33 | Superior longitudinal fasciculus L | 3.62 |
| Cluster 3 | 590 | -20 | 26 | -8 | Anterior corona radiata L | 3.38 |
| Cluster 4 | 390 | 2 | -17 | -29 | Middle cerebellar peduncle | 3.18 |
| Cluster 5 | 353 | -42 | -32 | -9 | Sagittal stratum L | 3.05 |
| Cluster 6 | 223 | -28 | -31 | 10 | Retrolenticular-internal capsule L | 2.97 |
| Cluster 7 | 101 | -30 | 12 | -5 | Inferior fronto-occip-fasciculus L | 2.81 |

For the DTI imaging, we only extracted FA maps for all subjects using the FMRIB diffusion toolbox. These extracted FA images were further processed and analyzed using the TBSS tool available in FSL. Table S20 shows the main affected areas in the AD vs. MCIs group obtained via DTI-FA imaging and the achieved voxel clusters including their detailed information. The minimum cluster size, in this case, was kept at 80 voxels because we found a huge number of changes in their WM brain regions while conducting two-sample *t-tests* in this set. Each cluster contained more than 80 adjacent voxels that displayed significant differences in those diffusion parameters. Table S20 shows that the right hemisphere of the posterior corona radiata region (2661 voxels) had a significant loss in WM volume when comparing the AD group with the MCIs group. Their peak intensity (*T-value*) value was 4.47.

**Table S21.** Cluster information (HC vs. MCIc) using sMRI imaging

| Cluster | Number of voxels | Peak MNI coordinates | | | Peak MNI coordinate region | Peak intensity  (T-Value) |
| --- | --- | --- | --- | --- | --- | --- |
|  |  | X | Y | Z |  |  |
| Cluster 1 | 1505 | 24 | -28 | 78 | Precentral_R | 5.97 |
| Cluster 2 | 733 | 24 | -61 | -64 | Paracentral_Lobule_R | 4.41 |
| Cluster 3 | 279 | 45 | -76 | -45 | Cerebelum_Crus2_R | 3.88 |
| Cluster 4 | 133 | 22 | -79 | -18 | Cerebelum_6_R | 3.67 |
| Cluster 5 | 79 | -3 | 9 | 4 | Caudate Head | 3.33 |
| Cluster 6 | 34 | 34 | -66 | 7 | Caudate_L | 2.60 |
| Cluster 7 | 15 | 31 | -63 | 36 | Occipital_Mid_R | 2.43 |
| Cluster 8 | 8 | 45 | 10 | 16 | Frontal_Inf_Oper_R | 2.33 |

Table S21 shows the main affected areas in the HC vs. MCIc groups obtained via sMRI imaging and the achieved voxel clusters with detailed information. The minimum cluster size, in this case, was kept at 5 voxels because we found a huge number of changes in their GM brain regions while operating two-sample *t-tests* in this set. The selected major voxels are shown with their *T-values*. Moreover, Table S21 shows that the right hemisphere of the precentral region (1505 voxels) displayed a significantly reduced GM volume when comparing the HC group with the MCIc group. Their peak intensity (*T-value*) score was 5.97.

**Table S22.** Cluster information (HC vs. MCIc) using FDG-PET imaging

| Cluster | Number of voxels | Peak MNI coordinates | | | Peak MNI coordinate region | Peak intensity  (T-Value) |
| --- | --- | --- | --- | --- | --- | --- |
|  |  | X | Y | Z |  |  |
| Cluster 1 | 1432 | -30 | -16 | 84 | Precentral_L | 4.74 |
| Cluster 2 | 1049 | 4 | -30 | 32 | Cingulum_Mid_L | 4.40 |
| Cluster 3 | 794 | 8 | -26 | 86 | Parietal_Sup_R | 4.29 |
| Cluster 4 | 463 | 2 | -80 | -30 | Cerebelum_6_L | 3.94 |
| Cluster 5 | 136 | -6 | -10 | 4 | Thalamus_L | 3.78 |
| Cluster 6 | 75 | 32 | 0 | 30 | Precentral_R | 3.54 |
| Cluster 7 | 42 | 48 | -16 | -18 | Middle Temporal Gyrus | 3.46 |
| Cluster 8 | 26 | 48 | 4 | -22 | Temporal_Pole_Mid_R | 3.20 |
| Cluster 9 | 9 | -30 | -32 | -22 | Parahippocampa Gyrus | 2.94 |

Table S22 shows the main affected area in the HC vs. MCIc group analyzed via FDG-PET imaging and the obtained voxel clusters with detailed information. The minimum cluster size, in this case, was kept at 5 voxels because we found a huge number of changes in their GM brain regions while conducting two-sample *t-tests* in this set. Each cluster contained more than 5 adjacent voxels that displayed the significant differences in those diffusion factors. The selected significant voxels are shown with their *T-values*. Table S22 also shows that the precentral region (1432 voxels) displayed a significantly reduced GM volume when comparing the HC group with the MCIc group. Their peak intensity (*T-value*) value was 4.74.

**Table S23.** Cluster information (HC vs. MCIc) using AV45-PET imaging

| Cluster | Number of voxels | Peak MNI coordinates | | | Peak MNI coordinate region | Peak intensity  (T-Value) |
| --- | --- | --- | --- | --- | --- | --- |
|  |  | X | Y | Z |  |  |
| Cluster 1 | 289 | 48 | 26 | -42 | Cerebelum_9_L | 3.02 |
| Cluster 2 | 171 | 2 | -36 | -24 | Frontal_Mid_L | 2.89 |
| Cluster 3 | 97 | -50 | -26 | -64 | Rolandic_Oper_L | 2.77 |
| Cluster 4 | 40 | -12 | -64 | 86 | Precuneus_L | 2.58 |
| Cluster 5 | 18 | 14 | -68 | 86 | Postcentral_R | 2.23 |
| Cluster 6 | 9 | 30 | -60 | 86 | Occipital_Inf_L | 2.01 |

Table S23 shows the main affected area in the HC vs. MCIc group obtained via AV45-PET imaging and the achieved voxel clusters including their detailed information. The minimum cluster size, in this case, was kept at 5 voxels because we found a huge number of changes in their GM brain regions while operating two-sample *t-tests* in this set. The selected significant voxels are shown with their *T-values*. Table S23 also shows that the left hemisphere of the cerebellum-9 region (289 voxels) displayed a significantly reduced GM volume when comparing the HC group with the MCIc group. Their peak intensity (*T-value*) value was 3.02.

**Table S24.** Cluster information (HC vs. MCIc) using rs-fMRI imaging

| Cluster | Number of voxels | | Peak MNI coordinates | | | Peak MNI coordinate region | Peak intensity  (T-Value) | |
| --- | --- | --- | --- | --- | --- | --- | --- | --- |
|  |  |  | X | Y | Z |  |  |  |
| ALFF | | | | | | | | |
| Cluster 1 | 20407 | | 12 | -30 | -12 | Precentral_L | | 6.34 |
| Cluster 2 | 202 | | 48 | -78 | -36 | Cerebelum_Crus1_R | | 5.51 |
| Cluster 3 | 186 | | 0 | -66 | 3 | Vermis_4_5 | | 4.65 |
| Cluster 4 | 140 | | -18 | -81 | -30 | Cerebelum_Crus1_L | | 4.60 |
| Cluster 5 | 70 | | 27 | -93 | 30 | Cuneus_R | | 4.47 |
| Cluster 6 | 58 | | 0 | -63 | 39 | Precuneus_L | | 4.31 |
| Cluster 7 | 21 | | 27 | -72 | 42 | Occipital_Sup_R | | 3.81 |
| Cluster 8 | 18 | | 24 | -66 | -21 | Cerebelum_6_R | | 3.71 |
| FALFF | | | | | | | | |
| Cluster 1 | 2118 | | -45 | -42 | 51 | Parietal_Inf_L | 6.11 | |
| Cluster 2 | 247 | | -27 | 3 | 54 | Frontal_Mid_L | 6.04 | |
| Cluster 3 | 186 | | -6 | -45 | 27 | Cingulum_Post_L | 5.76 | |
| Cluster 4 | 148 | | 42 | -45 | 39 | Parietal_Inf_R | 5.45 | |
| Cluster 5 | 77 | | 21 | 6 | 51 | Frontal_Sup_R | 5.31 | |
| Cluster 6 | 48 | | -48 | 6 | 33 | Precentral_L | 4.90 | |
| Cluster 7 | 30 | | -18 | -60 | -18 | Cerebelum_6_L | 4.85 | |
| REHO | | | | | | | | |
| Cluster 1 | | 1486 | -21 | -3 | 6 | Putamen_L | 6.50 | |
| Cluster 2 | | 111 | -33 | -9 | 12 | Insula_L | 5.47 | |
| Cluster 3 | | 55 | 33 | -36 | -27 | Cerebelum_6_R | 4.35 | |
| Cluster 4 | | 26 | -39 | -9 | -24 | Fusiform_L | 4.28 | |
| Cluster 5 | | 11 | 3 | 57 | 27 | Frontal-Sup-Medial_R | 3.69 | |

Table S24 shows the main affected areas in the HC vs. MCIc group obtained via rs-fMRI imaging and the obtained voxel clusters including their detailed information using three different whole-brain feature maps (ALFF, fALFF, REHO). The minimum cluster size for all cases was kept at 5 voxels because we found a huge number of changes in their GM brain regions while conducting two-sample *t-tests* in this set. Each cluster contained more than 5 adjacent voxels that displayed a significant difference in those diffusion factors. The selected significant voxels are shown with their *T-values*. Table S24 shows that, in the case of ALFF, the left hemisphere of the precentral region (20407 voxels) displayed a significantly reduced GM volume when comparing the HC group with the MCIc group. Their peak intensity (*T-value*) score was 6.34. Likewise, in the case of fALFF, the left hemisphere of the parietal-inf region (2118 voxels) had a significantly reduced GM volume. Their peak intensity value was 6.11. In the REHO feature map, the left hemisphere of the putamen region (1486 voxels) had a significantly reduced GM volume compared with other clusters. Its obtained peak intensity (*T-value*) value was 6.5.

**Table S25.** Cluster information (HC vs. MCIc) using DTI-FA imaging

| Cluster | Number of voxels | Peak MNI coordinates | | | Peak MNI coordinate region | Peak intensity  (T-Value) |
| --- | --- | --- | --- | --- | --- | --- |
|  |  | X | Y | Z |  |  |
| Cluster 1 | 706 | -35 | -64 | 26 | Superior longitudinal fasciculus L | 3.66 |
| Cluster 2 | 466 | 10 | -35 | -28 | Corticospinal tract R | 3.50 |
| Cluster 3 | 309 | -12 | 9 | -2 | Anterior limb-internal capsule L | 3.37 |
| Cluster 4 | 266 | -21 | -82 | 13 | Forceps major | 3.04 |
| Cluster 5 | 172 | 10 | -9 | -11 | Cerebral peduncle R | 2.92 |
| Cluster 6 | 71 | 29 | -68 | 23 | Inferior longitudinal fasciculus R | 2.41 |
| Cluster 7 | 26 | -15 | -16 | 59 | Corticospinal tract L | 2.31 |

For the DTI imaging, we only extracted FA maps for all subjects using the FMRIB diffusion toolbox. These extracted FA images were further processed and analyzed using the TBSS tool available in FSL. Table S25 shows the main affected areas in the HC vs. MCIc group obtained via DTI-FA imaging and the achieved voxel clusters including their detailed information. The minimum cluster size, in this case, was kept at 20 voxels because we found a huge number of changes in their WM brain regions while conducting two-sample *t-tests* in this set. Each cluster contained more than 20 adjacent voxels that displayed significant differences in those diffusion parameters. Table S25 shows that the left hemisphere of the superior longitudinal fasciculus region (706 voxels) had a significant loss in WM volume when comparing the HC group with the MCIs group. Their peak intensity (*T-value*) value was 3.66.

**Table S26.** Cluster information (HC vs. MCIs) using sMRI imaging

| Cluster | Number of voxels | Peak MNI coordinates | | | Peak MNI coordinate region | Peak intensity  (T-Value) |
| --- | --- | --- | --- | --- | --- | --- |
|  |  | X | Y | Z |  |  |
| Cluster 1 | 369 | -24 | -82 | -16 | Lingual_L | 4.29 |
| Cluster 2 | 153 | -12 | -16 | 51 | Medial Frontal Gyrus | 3.67 |
| Cluster 3 | 103 | 52 | 43 | 13 | Frontal_Mid_R | 3.65 |
| Cluster 4 | 75 | -12 | -4 | 1 | Pallidum_L | 3.62 |
| Cluster 5 | 30 | 18 | -52 | 48 | Precuneus | 3.53 |

Table S26 shows the main affected areas in the HC vs. MCIs groups obtained via sMRI imaging and the achieved voxel clusters with detailed information. The minimum cluster size, in this case, was kept at 20 voxels because we found a huge number of changes in their GM brain regions while operating two-sample *t-tests* in this set. The selected major voxels are shown with their *T-values*. Moreover, Table S26 shows that the left hemisphere of the lingual region (369 voxels) displayed a significantly reduced GM volume when comparing the HC group with the MCIs group. Their peak intensity (*T-value*) score was 4.29.

**Table S27.** Cluster information (HC vs. MCIs) using FDG-PET imaging

| Cluster | Number of voxels | Peak MNI coordinates | | | Peak MNI coordinate region | Peak intensity  (T-Value) |
| --- | --- | --- | --- | --- | --- | --- |
|  |  | X | Y | Z |  |  |
| Cluster 1 | 1055 | -20 | -4 | -1.81 | Occipital_Sup_L | 4.53 |
| Cluster 2 | 970 | 11 | -38 | 1.79 | Paracentral_Lobule_L | 4.31 |
| Cluster 3 | 879 | -17 | -35 | 5.39 | ParaHippocampal_L | 3.30 |
| Cluster 4 | 248 | 15 | -31 | 1.79 | Amygdala_R | 2.74 |
| Cluster 5 | 27 | -14 | -34 | -25 | Cerebelum_10_L | 2.32 |
| Cluster 6 | 10 | 39 | -4 | 1.79 | Vermis_3 | 2.21 |

Table S27 shows the main affected area in the HC vs. MCIs group analyzed via FDG-PET imaging and the obtained voxel clusters with detailed information. The minimum cluster size, in this case, was kept at 5 voxels because we found a huge number of changes in their GM brain regions while conducting two-sample *t-tests* in this set. Each cluster contained more than 5 adjacent voxels that displayed the significant differences in those diffusion factors. The selected significant voxels are shown with their *T-values*. Table S27 shows that the occipital-sup region (1055 voxels) displayed a significantly reduced GM volume when comparing the HC group with the MCIs group. Their peak intensity (*T-value*) value was 4.53.

**Table S28.** Cluster information (HC vs. MCIs) using AV45-PET imaging

| Cluster | Number of voxels | Peak MNI coordinates | | | Peak MNI coordinate region | Peak intensity  (T-Value) |
| --- | --- | --- | --- | --- | --- | --- |
|  |  | X | Y | Z |  |  |
| Cluster 1 | 846 | -22 | 20 | 4 | Frontal_Inf_Oper_R | 4.36 |
| Cluster 2 | 657 | -10 | -4 | -4 | Insula_R | 4.22 |
| Cluster 3 | 195 | -12 | 32 | -26 | Thalamus_L | 3.59 |
| Cluster 4 | 125 | 44 | 22 | 24 | Frontal_Sup_Orb_L | 3.58 |
| Cluster 5 | 88 | -8 | -28 | 0 | Frontal_Inf_Tri_R | 3.38 |

Table S28 shows the main affected area in the HC vs. MCIs group obtained via AV45-PET imaging and the achieved voxel clusters including their detailed information. The minimum cluster size, in this case, was kept at 50 voxels because we found a huge number of changes in their GM brain regions while operating two-sample *t-tests* in this set. The selected significant voxels are shown with their *T-values*. Table S28 also shows that the right hemisphere of the frontal-inf region (846 voxels) displayed a significantly reduced GM volume when comparing the HC group with the MCIs group. Their peak intensity (*T-value*) value was 4.36.

**Table S29.** Cluster information (HC vs. MCIs) using rs-fMRI imaging

| Cluster | Number of voxels | | Peak MNI coordinates | | | Peak MNI coordinate region | Peak intensity  (T-Value) | |
| --- | --- | --- | --- | --- | --- | --- | --- | --- |
|  |  |  | X | Y | Z |  |  |  |
| ALFF | | | | | | | | |
| Cluster 1 | 316 | | -39 | 48 | -15 | Frontal_Inf_Orb_L | | 2.87 |
| Cluster 2 | 233 | | -60 | -36 | -24 | Temporal_Inf_L | | 2.75 |
| Cluster 3 | 113 | | -15 | -60 | 63 | Precuneus_L | | 2.67 |
| Cluster 4 | 110 | | 9 | 63 | 6 | Frontal_Sup_Medial_R | | 2.65 |
| Cluster 5 | 70 | | 41 | -12 | 33 | Postcentral_R | | 2.29 |
| Cluster 6 | 54 | | -15 | 3 | -18 | Olfactory_L | | 2.19 |
| Cluster 7 | 23 | | 39 | -87 | -12 | Occipital_Inf_R | | 1.47 |
| FALFF | | | | | | | | |
| Cluster 1 | 828 | | 18 | 57 | -12 | Frontal_Sup_Orb_R | 4.19 | |
| Cluster 2 | 234 | | 48 | -6 | 27 | Precentral_R | 3.26 | |
| Cluster 3 | 185 | | -21 | -45 | -9 | Lingual_L | 3.25 | |
| Cluster 4 | 78 | | 39 | 48 | -15 | Frontal_Mid_Orb_R | 3.02 | |
| Cluster 5 | 44 | | -12 | -57 | 57 | Precuneus_L | 2.94 | |
| Cluster 6 | 22 | | 36 | -30 | -21 | Fusiform_R | 2.82 | |
| REHO | | | | | | | | |
| Cluster 1 | | 781 | -21 | -48 | -9 | Lingual_L | 4.74 | |
| Cluster 2 | | 136 | 51 | -6 | -3 | Temporal_Sup_R | 4.56 | |
| Cluster 3 | | 77 | 27 | -42 | -9 | Fusiform_R | 4.01 | |
| Cluster 4 | | 22 | -66 | -24 | -6 | Temporal_Mid_L | 3.68 | |
| Cluster 5 | | 5 | 24 | 6 | -18 | Amygdala_R | 3.15 | |

Table S29 shows the main affected areas in the HC vs. MCIs group obtained via rs-fMRI imaging and the obtained voxel clusters including their detailed information using three different whole-brain feature maps (ALFF, fALFF, REHO). The minimum cluster size for all cases was kept at 5 voxels because we found a huge number of changes in their GM brain regions while conducting two-sample *t-tests* in this set. Each cluster contained more than 5 adjacent voxels that displayed a significant difference in those diffusion factors. The selected significant voxels are shown with their *T-values*. Table S29 shows that, in the case of ALFF, the left hemisphere of the frontal-inf-orb region (316 voxels) displayed a significantly reduced GM volume when comparing the HC group with the MCIs group. Their peak intensity (*T-value*) score was 2.87. Likewise, in the case of fALFF, the right hemisphere of the frontal-sup-orb region (828 voxels) had a significantly reduced GM volume. Their peak intensity value was 4.19. In the REHO feature map, the left hemisphere of the lingual region (781 voxels) had a significantly reduced GM volume compared with other clusters. Its obtained peak intensity (*T-value*) value was 4.74.

**Table S30.** Cluster information (HC vs. MCIs) using DTI-FA imaging

| Cluster | Number of voxels | Peak MNI coordinates | | | Peak MNI coordinate region | Peak intensity  (T-Value) |
| --- | --- | --- | --- | --- | --- | --- |
|  |  | X | Y | Z |  |  |
| Cluster 1 | 3144 | -20 | -2 | 34 | Superior corona radiata L | 3.53 |
| Cluster 2 | 1558 | -46 | -28 | -13 | Superior fasciculus L | 3.20 |
| Cluster 3 | 211 | 49 | -19 | -26 | Splenium of corpus callosum | 3.16 |
| Cluster 4 | 173 | 33 | 34 | 23 | Uncinate fasciculus L | 3.15 |
| Cluster 5 | 118 | 26 | -30 | 32 | Posterior corona radiata R | 3.11 |
| Cluster 6 | 75 | 23 | 38 | 23 | Anterior thalamic radiation R | 2.52 |
| Cluster 7 | 51 | -28 | -74 | 11 | Inferior longitudinal fasciculus L | 2.43 |
| Cluster 8 | 23 | 0 | -42 | -46 | Corticospinal tract R | 2.06 |

For the DTI imaging, we only extracted FA maps for all subjects using the FMRIB diffusion toolbox. These extracted FA images were further processed and analyzed using the TBSS tool available in FSL. Table S30 shows the main affected areas in the HC vs. MCIs group obtained via DTI-FA imaging and the achieved voxel clusters including their detailed information. The minimum cluster size, in this case, was kept at 20 voxels because we found a huge number of changes in their WM brain regions while conducting two-sample *t-tests* in this set. Each cluster contained more than 20 adjacent voxels that displayed significant differences in those diffusion parameters. Table S30 shows that the left hemisphere of the superior corona radiata region (3144 voxels) had a significant loss in WM volume when comparing the HC group with the MCIs group. Their peak intensity (*T-value*) value was 3.53.

**Table S31.** Classification results for AD vs. HC, MCIs vs. MCIc, AD vs. MCIs, AD vs. MCIc, HC vs. MCIc, and HC vs. MCIs groups using ROI features (RBF-SVM).

| **Groups** | **Features** | **Classifier** | **Performance measure** | | | | | | |
| --- | --- | --- | --- | --- | --- | --- | --- | --- | --- |
|  |  |  | **AUC** | **ACC** | **SEN** | **SPEC** | **PRE** | **F1-score** | **Cohen’s kappa** |
| **AD vs. HC** | APOE | RBF-SVM | 81.43 | 79.02 | 85.67 | 62.31 | 79.58 | 82.52 | 0.6445 |
|  | sMRI |  | 90.61 | 90.35 | **92.87** | **93.55** | 90.84 | 91.84 | 0.7212 |
|  | FDG-PET |  | 90.35 | 89.71 | 89.16 | 91.57 | 90.88 | 90.01 | 0.8065 |
|  | AV45-PET |  | 89.06 | 89.22 | 86.09 | 89.26 | 88.97 | 87.5 | 0.7047 |
|  | rs-fMRI |  | 91.88 | 91.85 | 89.45 | 88.69 | 88.89 | 89.16 | 0.8371 |
|  | DTI-FA |  | 89.94 | 90.93 | 91.21 | 89.88 | 90.31 | 90.75 | 0.7812 |
|  | **Combined-ROI** |  | **93.77** | **93.84** | 92.78 | 91.54 | **92.5** | **92.63** | **0.8848** |
| **MCIs vs. MCIc** | APOE | RBF-SVM | 75.98 | 75.68 | 76.18 | 65.54 | 67.67 | 71.67 | 0.6142 |
|  | sMRI |  | 88.19 | 87.69 | 83.74 | 78.88 | 84.96 | 84.34 | 0.6968 |
|  | FDG-PET |  | 90.17 | 90.35 | 89.72 | **91.38** | 89.26 | 89.48 | 0.7275 |
|  | AV45-PET |  | 88.5 | 89.42 | 88.81 | 85.08 | **91.92** | **90.33** | 0.7176 |
|  | rs-fMRI |  | 87.18 | 86.02 | 86.65 | 72.36 | 81.71 | 84.1 | 0.6993 |
|  | DTI-FA |  | 90.61 | 89.29 | 79.41 | 88.37 | 85.24 | 82.22 | 0.7064 |
|  | **Combined-ROI** |  | **91.67** | **91.42** | **92.3** | 90.9 | 85.71 | 88.89 | **0.7634** |
| **AD vs. MCIs** | APOE | RBF-SVM | 79.28 | 78.47 | 73.14 | 71.52 | 78.94 | 75.92 | 0.6231 |
|  | sMRI |  | 86.21 | 85.07 | 84.02 | 76.05 | 85.73 | 84.86 | 0.6924 |
|  | FDG-PET |  | 91.67 | 90.39 | 90.61 | 88.5 | **91.6** | 91.1 | 0.7854 |
|  | AV45-PET |  | 87.97 | 87.94 | 85.12 | 78.88 | 85.05 | 85.08 | 0.6846 |
|  | rs-fMRI |  | 88.25 | 89.19 | 83.73 | 79.2 | 85.17 | 84.44 | 0.707 |
|  | DTI-FA |  | 90.97 | 90.81 | 87.27 | 91.95 | 90.18 | 88.7 | 0.7611 |
|  | **Combined-ROI** |  | **92.05** | **92.74** | **100** | **91.67** | 87.5 | **93.33** | **0.8902** |
| **AD vs. MCIc** | APOE | RBF-SVM | 81.68 | 78.47 | 82.81 | 68.43 | 78.84 | 80.77 | 0.6373 |
|  | sMRI |  | 86.24 | 86.14 | 82.18 | 72.48 | 80.93 | 81.55 | 0.6586 |
|  | FDG-PET |  | 88.26 | 87.01 | 85.71 | 79.8 | 85.3 | 85.5 | 0.7074 |
|  | AV45-PET |  | 87.16 | 87.57 | 88.93 | 80.78 | 91.53 | 90.21 | 0.6873 |
|  | rs-fMRI |  | 90.43 | 89.01 | 87.25 | 90.32 | 88.19 | 87.71 | 0.7244 |
|  | DTI-FA |  | 90.58 | 90.11 | 89.33 | 93.88 | 94.19 | **91.69** | 0.745 |
|  | **Combined-ROI** |  | **92.19** | **91.29** | **90** | **100** | **100** | 88.9 | **0.7879** |
| **HC vs. MCIc** | APOE | RBF-SVM | 75.48 | 71.43 | 72.78 | 69.48 | 77.67 | 75.14 | 0.6193 |
|  | sMRI |  | 88.96 | 88.6 | 89.7 | 92.68 | 90.65 | 91.17 | 0.7044 |
|  | FDG-PET |  | 86.57 | 86.233 | 82.9 | 89.01 | 86.45 | 84.63 | 0.6882 |
|  | AV45-PET |  | 90.17 | 90.11 | 87.64 | 92.17 | 90.45 | 89.02 | 0.7869 |
|  | rs-fMRI |  | 89.45 | 88.25 | 84 | 92.86 | 92.94 | 88.24 | 0.7637 |
|  | DTI-FA |  | 91.89 | 92.27 | 89.53 | **95.24** | **95.42** | 92.38 | 0.7911 |
|  | **Combined-ROI** |  | **92.76** | **93.47** | **100** | 90 | 90 | **94.74** | **0.8134** |
| **HC vs. MCIs** | APOE | RBF-SVM | 78.19 | 77.63 | 70.24 | 72.3 | 75.41 | 72.73 | 0.6342 |
|  | sMRI |  | 86.1 | 85.17 | 80.54 | 76.78 | 85.55 | 82.96 | 0.6513 |
|  | FDG-PET |  | 90.06 | 89.14 | 86.53 | 78.8 | 85.36 | 85.94 | 0.7292 |
|  | AV45-PET |  | 87.77 | 87.43 | 84.44 | 79.31 | 83.62 | 84.32 | 0.6966 |
|  | rs-fMRI |  | 89.05 | 88.25 | 86.99 | 78.1 | 84.93 | 85.94 | 0.7441 |
|  | DTI-FA |  | 90.81 | 89.95 | 83.92 | **89.48** | **87.1** | 85.48 | 0.7799 |
|  | **Combined-ROI** |  | **91.46** | **92.3** | **100** | 87.5 | 83.34 | **90.9** | **0.8231** |

**Table S32.** Classification results for AD vs. HC, MCIs vs. MCIc, AD vs. MCIs, AD vs. MCIc, HC vs. MCIc, and HC vs. MCIs groups using voxel-wise (VOI) features (RBF-SVM).

| **Groups** | **Features** | **Classifier** | **Performance measure** | | | | | | |
| --- | --- | --- | --- | --- | --- | --- | --- | --- | --- |
|  |  |  | **AUC** | **ACC** | **SEN** | **SPEC** | **PRE** | **F1-score** | **Cohen’s kappa** |
| **AD vs. HC** | APOE | RBF-SVM | 81.43 | 79.02 | 85.67 | 62.31 | 79.58 | 82.52 | 0.6445 |
|  | sMRI |  | 88.65 | 87.55 | 88.96 | 85.17 | 92.12 | 90.51 | 0.7834 |
|  | FDG-PET |  | 89.95 | 89.21 | 89.56 | **91.9** | 87.99 | 88.76 | 0.7861 |
|  | AV45-PET |  | 91.61 | 91.73 | 91.53 | 89.76 | 93.46 | 92.48 | 0.8245 |
|  | rs-fMRI-ALFF |  | 87.45 | 87.03 | 90.25 | 83.6 | 89.35 | 89.79 | 0.7413 |
|  | rs-fMRI-fALFF |  | 88.93 | 89.88 | 87.78 | 85.14 | 92.73 | 90.18 | 0.7857 |
|  | rs-fMRI-REHO |  | 90.06 | 91.75 | 91.37 | 89.61 | 93.51 | 92.47 | 0.8003 |
|  | DTI-FA |  | 87.47 | 87.06 | 90.38 | 86.14 | 90.81 | 90.59 | 0.7536 |
|  | **Combined-VOI** |  | **93.02** | **93.81** | **92.93** | 90.84 | **95.21** | **94.05** | **0.8674** |
| **MCIs vs. MCIc** | APOE | RBF-SVM | 75.98 | 75.68 | 76.18 | 65.54 | 67.67 | 71.67 | 0.6142 |
|  | sMRI |  | 88.56 | 87.85 | 88.87 | 85.05 | 92.07 | 90.44 | 0.7376 |
|  | FDG-PET |  | 85.93 | 84.21 | 86.16 | 83.45 | 88.41 | 87.27 | 0.6883 |
|  | AV45-PET |  | 87.47 | 86.91 | 88.13 | 84.63 | 91.04 | 89.56 | 0.7122 |
|  | rs-fMRI-ALFF |  | 91.25 | 90.55 | 91.11 | 88.42 | 94.09 | 92.57 | 0.7751 |
|  | rs-fMRI-fALFF |  | 89.62 | 89.92 | 89.84 | 87.22 | 92.02 | 90.96 | 0.7688 |
|  | rs-fMRI-REHO |  | 90.66 | 89.45 | 90.55 | 88.05 | 93.26 | 91.88 | 0.7737 |
|  | DTI-FA |  | 91.64 | 91.55 | 91.36 | 89.23 | 93.68 | 92.5 | 0.7964 |
|  | **Combined-VOI** |  | **92.94** | **92.14** | **92.87** | **91.09** | **94.8** | **93.82** | **0.8312** |
| **AD vs. MCIs** | APOE | RBF-SVM | 79.28 | 78.47 | 73.14 | 71.52 | 78.94 | 75.92 | 0.6231 |
|  | sMRI |  | 88.29 | 88.39 | 88.17 | 85.4 | 90.11 | 89.12 | 0.7753 |
|  | FDG-PET |  | 88.56 | 89.47 | **93.75** | 83.34 | 88.23 | 90.9 | 0.7403 |
|  | AV45-PET |  | 90.16 | 89.43 | 90.38 | 87.63 | 89.68 | 90.02 | 0.7949 |
|  | rs-fMRI-ALFF |  | 88.96 | 89.31 | 89.3 | 85.14 | 90.77 | 90.02 | 0.7637 |
|  | rs-fMRI-fALFF |  | 89.27 | 89.78 | 89.19 | 87.22 | 89.33 | 89.25 | 0.7879 |
|  | rs-fMRI-REHO |  | 87.09 | 88.81 | 90.32 | 87 | 91.94 | 91.12 | 0.7445 |
|  | DTI-FA |  | 90.47 | 90.2 | 90.33 | 87.07 | 91.87 | 91.09 | 0.8108 |
|  | **Combined-VOI** |  | **92.04** | **92.16** | 91.93 | **89.41** | **94.04** | **92.97** | **0.8544** |
| **AD vs. MCIc** | APOE | RBF-SVM | 81.68 | 78.47 | 82.81 | 68.43 | 78.84 | 80.77 | 0.6373 |
|  | sMRI |  | 87.81 | 88.67 | 88.04 | 85.23 | 90.39 | 89.19 | 0.7403 |
|  | FDG-PET |  | 88.05 | 87.96 | 88.35 | 84.72 | 91.38 | 89.83 | 0.764 |
|  | AV45-PET |  | 90.01 | 89.48 | 90.3 | 86.81 | 93.21 | 91.73 | 0.7716 |
|  | rs-fMRI-ALFF |  | 87.8 | 88.44 | 87.99 | 85.95 | 89.95 | 88.43 | 0.7566 |
|  | rs-fMRI-fALFF |  | 89.78 | 89.53 | 92.03 | 89.77 | **94.49** | 93.24 | 0.7839 |
|  | rs-fMRI-REHO |  | 90.18 | 90.34 | 90.45 | 87.13 | 93.23 | 91.84 | 0.8041 |
|  | DTI-FA |  | 89.33 | 90.24 | 91.23 | 88.96 | 93.7 | 92.44 | 0.7877 |
|  | **Combined-VOI** |  | **91.77** | **92.59** | **93.34** | **91.57** | 93.33 | **93.34** | **0.8218** |
| **HC vs. MCIc** | APOE | RBF-SVM | 75.48 | 71.43 | 72.78 | 69.48 | 77.67 | 75.14 | 0.6193 |
|  | sMRI |  | 87.66 | 87.06 | 86.31 | 86.53 | 89.8 | 88.02 | 0.754 |
|  | FDG-PET |  | 89.46 | 90.59 | 89.73 | 86.4 | 90.51 | 90.11 | 0.7967 |
|  | AV45-PET |  | 88.5 | 88.42 | 88.81 | 85.08 | 91.92 | 90.33 | 0.7791 |
|  | rs-fMRI-ALFF |  | 90.17 | 89.76 | 90 | 87.82 | 90.52 | 90.25 | 0.8007 |
|  | rs-fMRI-fALFF |  | 89.01 | 88.98 | 88.35 | 85.43 | 90.79 | 89.55 | 0.7889 |
|  | rs-fMRI-REHO |  | 88.57 | 89.88 | 87.82 | 85.32 | 91.75 | 89.74 | 0.7855 |
|  | DTI-FA |  | 90.93 | 90.48 | 89.81 | 87.21 | 92.65 | 91.06 | 0.8123 |
|  | **Combined-VOI** |  | **93.63** | **93.83** | **93.55** | **91.74** | **95.47** | **94.5** | **0.8621** |
| **HC vs. MCIs** | APOE | RBF-SVM | 78.19 | 77.63 | 70.24 | 72.3 | 75.41 | 72.73 | 0.6342 |
|  | sMRI |  | 90.05 | 89.97 | 89.91 | 86.56 | 88.54 | 89.21 | 0.7889 |
|  | FDG-PET |  | 88.56 | 88.7 | 88.14 | 90.11 | 91.66 | 89.86 | 0.763 |
|  | AV45-PET |  | 89.06 | 90.81 | 89.83 | 86.52 | 92.6 | 91.19 | 0.7791 |
|  | rs-fMRI-ALFF |  | 88.84 | 88.5 | 90.22 | 84.61 | 87.07 | 88.61 | 0.7517 |
|  | rs-fMRI-fALFF |  | 88.64 | 87.09 | 88.95 | 85.15 | 91.13 | 90.02 | 0.7428 |
|  | rs-fMRI-REHO |  | 90.63 | 90.73 | 90.52 | 87.84 | 89.42 | 89.96 | 0.7855 |
|  | DTI-FA |  | 91.29 | 91.58 | 91.2 | 89.05 | **92.53** | 91.86 | 0.8041 |
|  | **Combined-VOI** |  | **92.14** | **92.92** | **100** | **93.33** | 92.3 | **96** | **0.8334** |

**Table S33.** Classification result of AD vs. HC, MCIs vs. MCIc, AD vs. MCIs, AD vs. MCIc, HC vs. MCIc, and HC vs. MCIs groups using combined-(VOI+ROI) features, with both whole-brain and voxel-wise features (RBF-SVM).

| **Groups** | **Features** | **Classifier** | **Performance measure** | | | | | | |
| --- | --- | --- | --- | --- | --- | --- | --- | --- | --- |
|  |  |  | **AUC** | **ACC** | **SEN** | **SPEC** | **PRE** | **F1-score** | **Cohen’s kappa** |
| **AD vs. HC** | Combined-ROI | RBF-SVM | 93.77 | 93.84 | 92.78 | 91.54 | 92.5 | 92.63 | 0.8848 |
|  | Combined-VOI |  | 93.02 | 93.81 | 92.93 | 90.84 | **95.21** | 94.05 | 0.8674 |
|  | **Combined (VOI+ROI)** |  | **95.55** | **95.93** | **95.17** | **92.3** | 94.45 | **94.8** | **0.9036** |
| **MCIs vs. MCIc** | Combined-ROI | RBF-SVM | 91.67 | 91.42 | 92.3 | 90.9 | 85.71 | 88.89 | 0.7634 |
|  | Combined-VOI |  | 92.94 | 92.14 | 92.87 | **91.09** | **94.8** | **93.82** | 0.8312 |
|  | **Combined (VOI+ROI)** |  | **94.48** | **93.75** | **100** | 90.9 | 83.33 | 90.91 | **0.8445** |
| **AD vs. MCIs** | Combined-ROI | RBF-SVM | 92.05 | 92.74 | 100 | 91.67 | 87.5 | 93.33 | 0.8902 |
|  | Combined-VOI |  | 92.04 | 92.16 | 91.93 | 89.41 | **94.04** | 92.97 | 0.8544 |
|  | **Combined (VOI+ROI)** |  | **95.18** | **96.15** | **100** | **91.67** | 93.33 | **96.55** | **0.9122** |
| **AD vs. MCIc** | Combined-ROI | RBF-SVM | 92.19 | 91.29 | 90 | 100 | 100 | 88.9 | 0.7879 |
|  | Combined-VOI |  | 91.77 | 92.59 | **93.34** | 91.57 | **93.33** | **93.34** | 0.8218 |
|  | **Combined (VOI+ROI)** |  | **94.09** | **94.11** | 92.86 | **95** | 92.85 | 92.85 | **0.8872** |
| **HC vs. MCIc** | Combined-ROI | RBF-SVM | 92.76 | 93.47 | 100 | 90 | 90 | 94.74 | 0.8134 |
|  | Combined-VOI |  | 93.63 | 93.83 | 93.55 | **91.74** | **95.47** | 94.5 | 0.8621 |
|  | **Combined (VOI+ROI)** |  | **94** | **95.23** | **100** | 91.5 | 92.86 | **96.3** | **0.8919** |
| **HC vs. MCIs** | Combined-ROI | RBF-SVM | 91.46 | 92.3 | 100 | 87.5 | 83.34 | 90.9 | 0.8231 |
|  | Combined-VOI |  | 92.14 | 92.92 | **100** | 93.33 | 92.3 | **96** | 0.8334 |
|  | **Combined (VOI+ROI)** |  | **93.23** | **94.59** | 86.67 | **100** | **100** | 92.85 | **0.8699** |


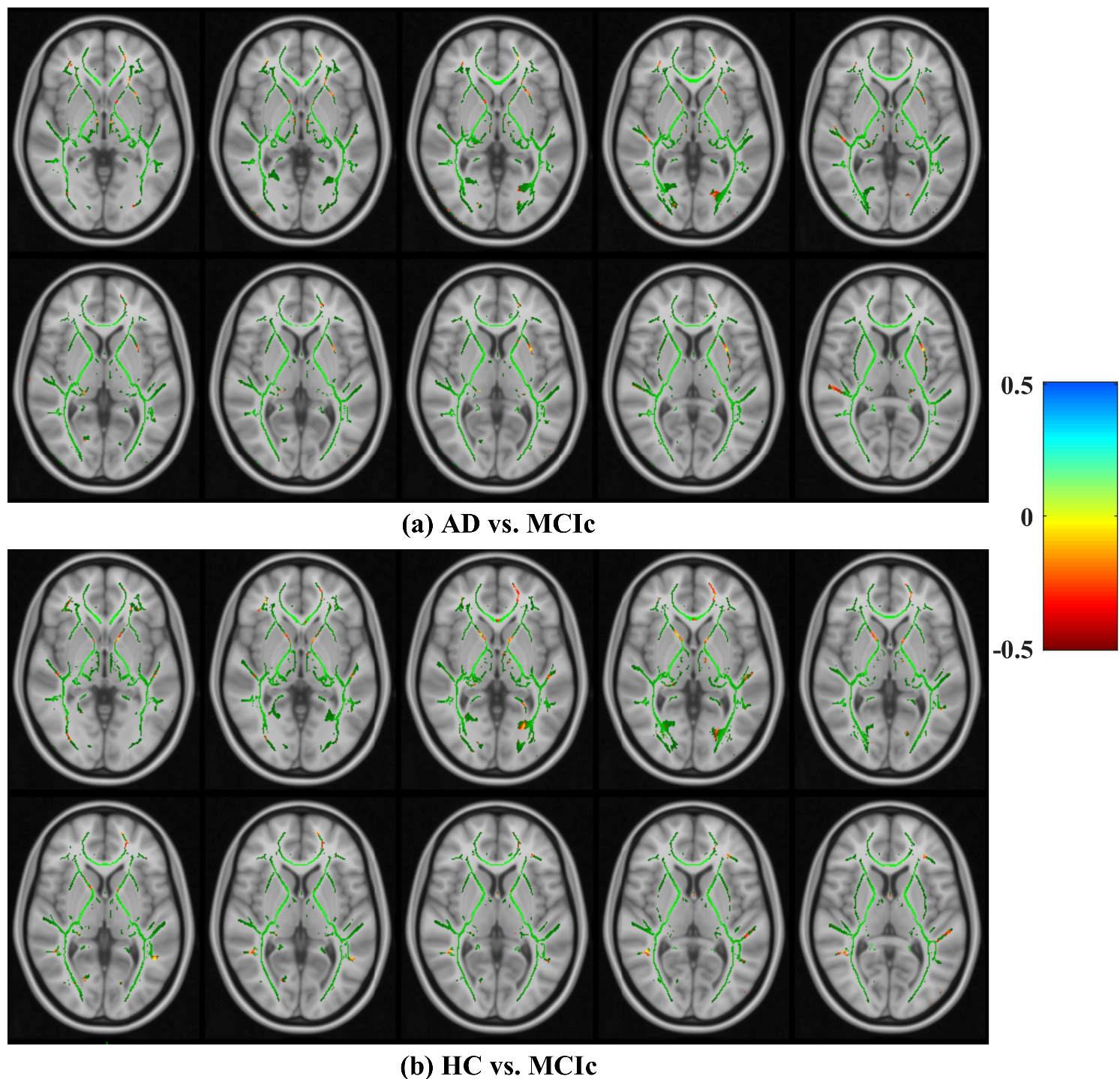


**Figure S1.** Selected WM voxels for the (a) AD vs. MCIc, and (b) HC vs. MCIc classification using DTI image.


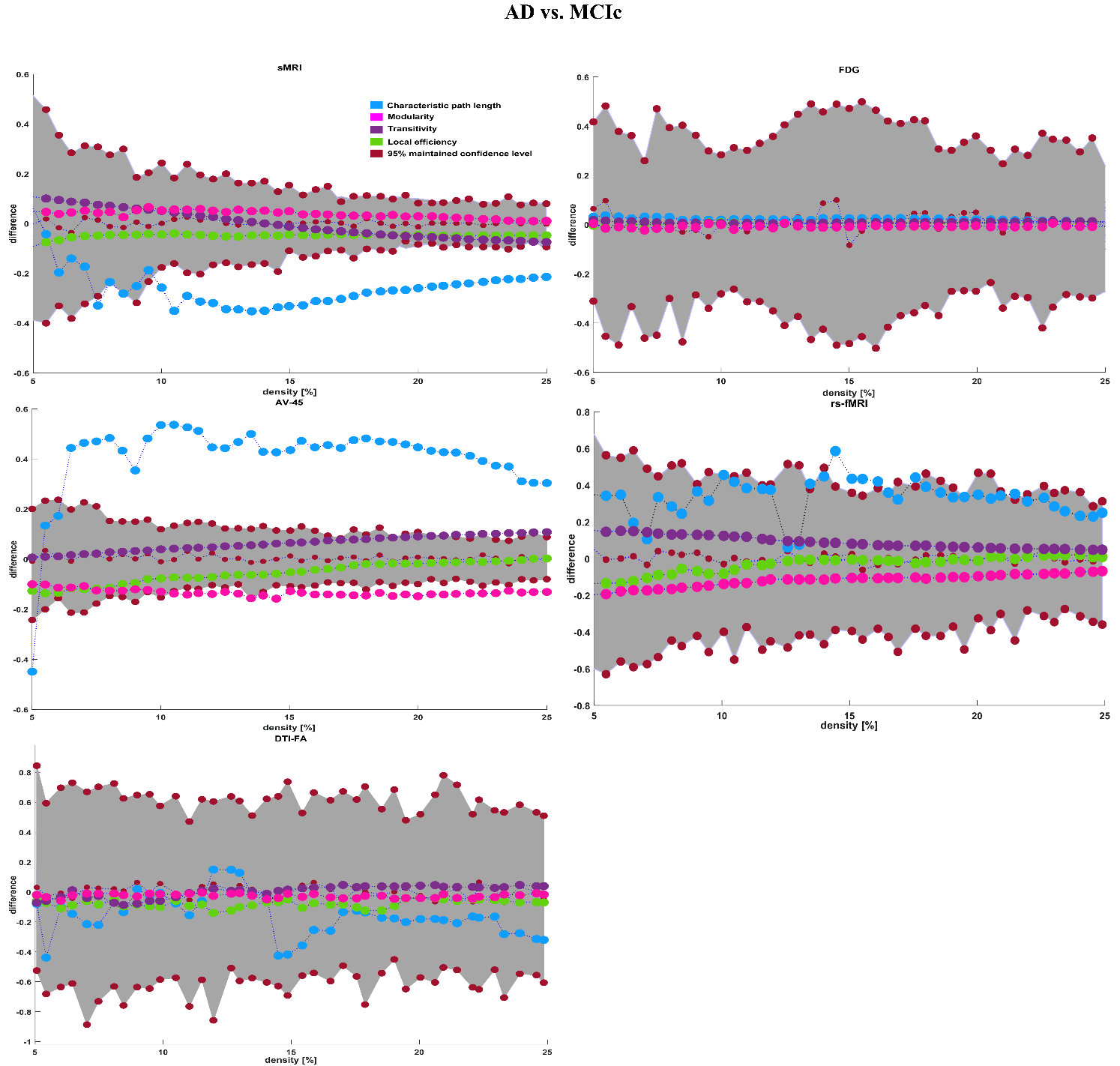


**Figure S2.** Differences between the AD vs. MCIc group in global structural topology. The blue sphere represents characteristics path length, green sphere represents local efficiency, pink sphere represents modularity, purple sphere represents transitivity, and the dark red sphere represents 95% confidence intervals for these measures.


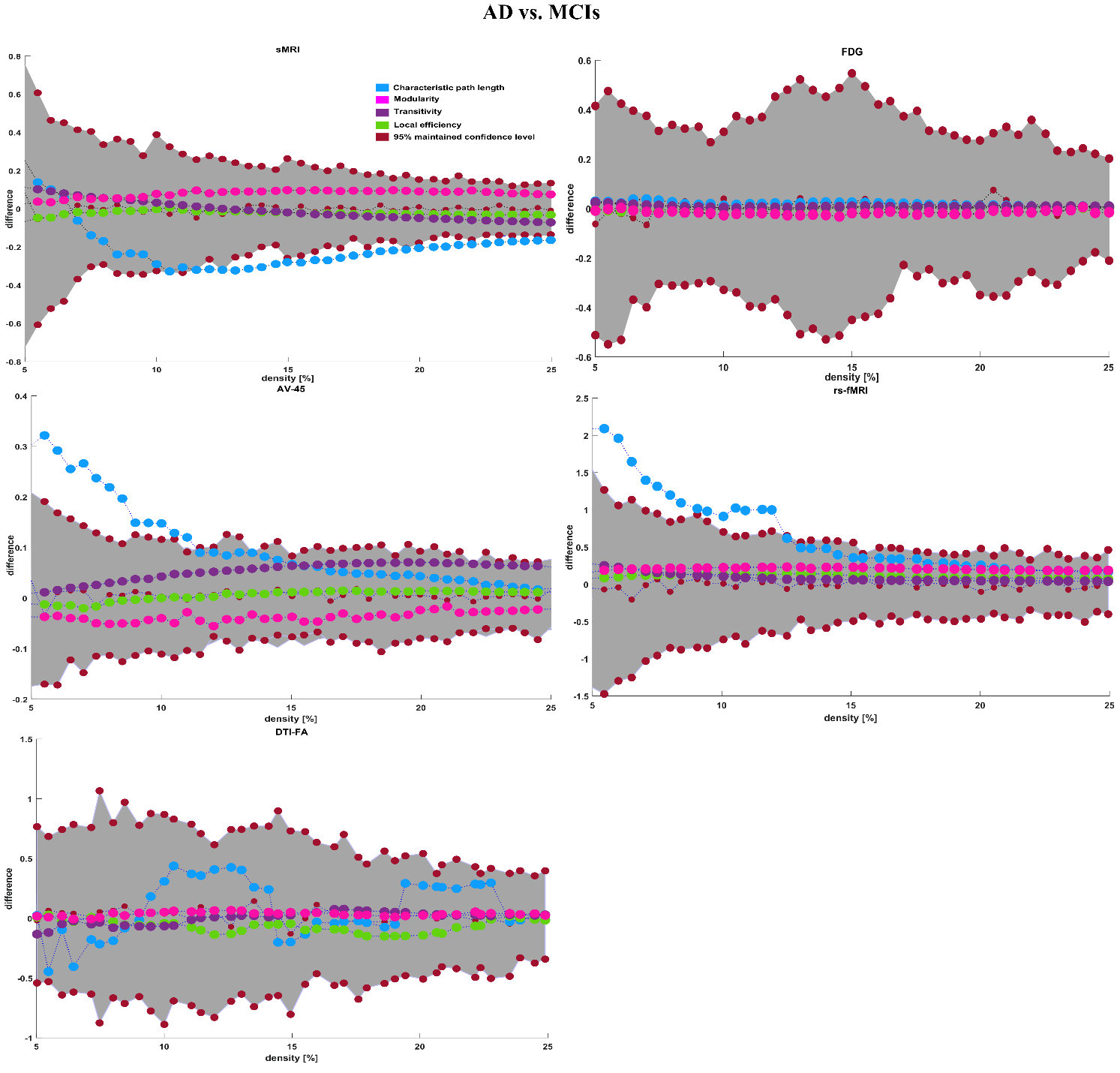


**Figure S3.** Differences between the AD vs. MCIs group in global structural topology. The blue sphere represents characteristics path length, green sphere represents local efficiency, pink sphere represents modularity, purple sphere represents transitivity, and the dark red sphere represents 95% confidence intervals for these measures.


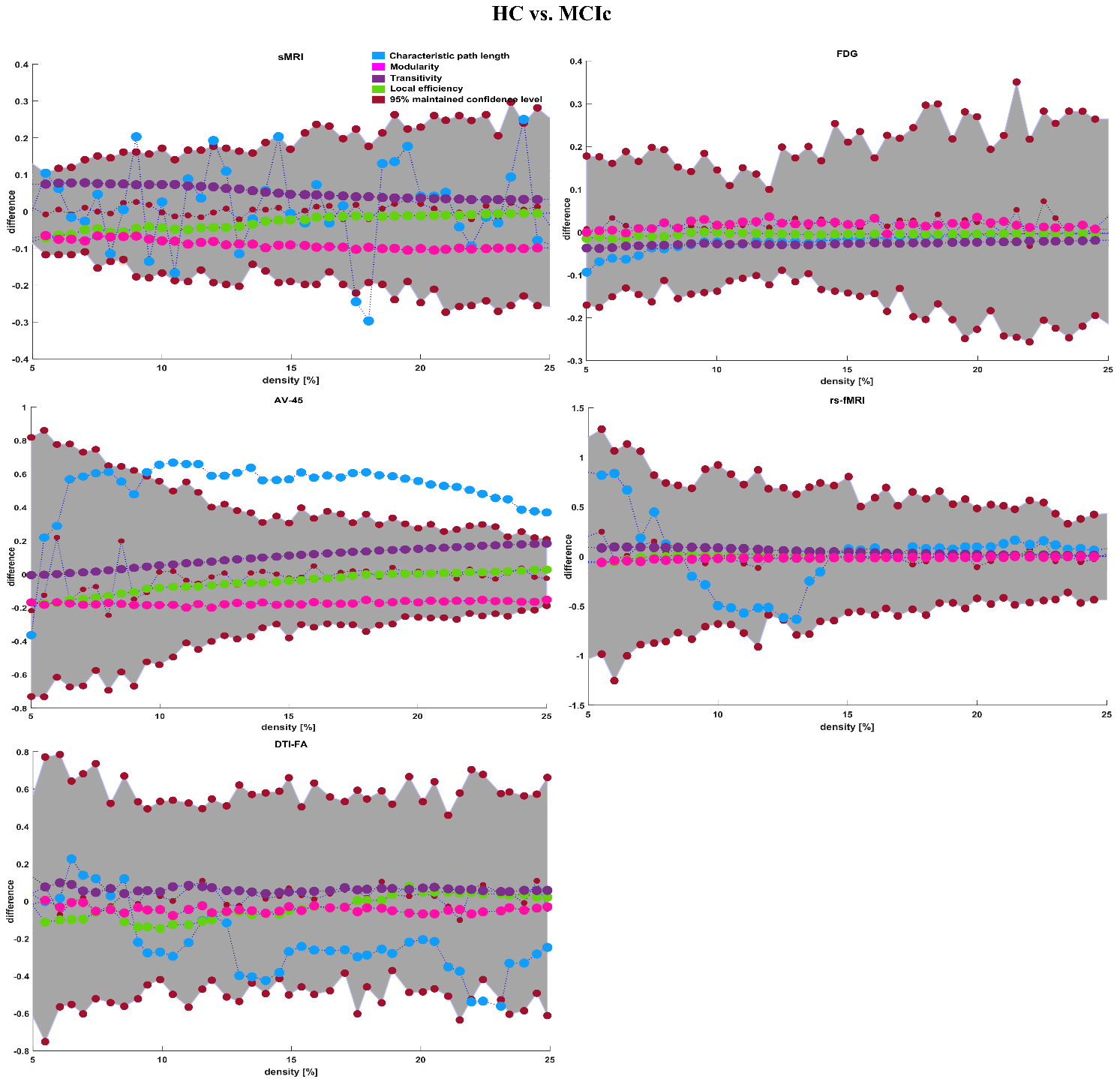


**Figure S4.** Differences between the HC vs. MCIc group in global structural topology. The blue sphere represents characteristics path length, green sphere represents local efficiency, pink sphere represents modularity, purple sphere represents transitivity, and the dark red sphere represents 95% confidence intervals for these measures.


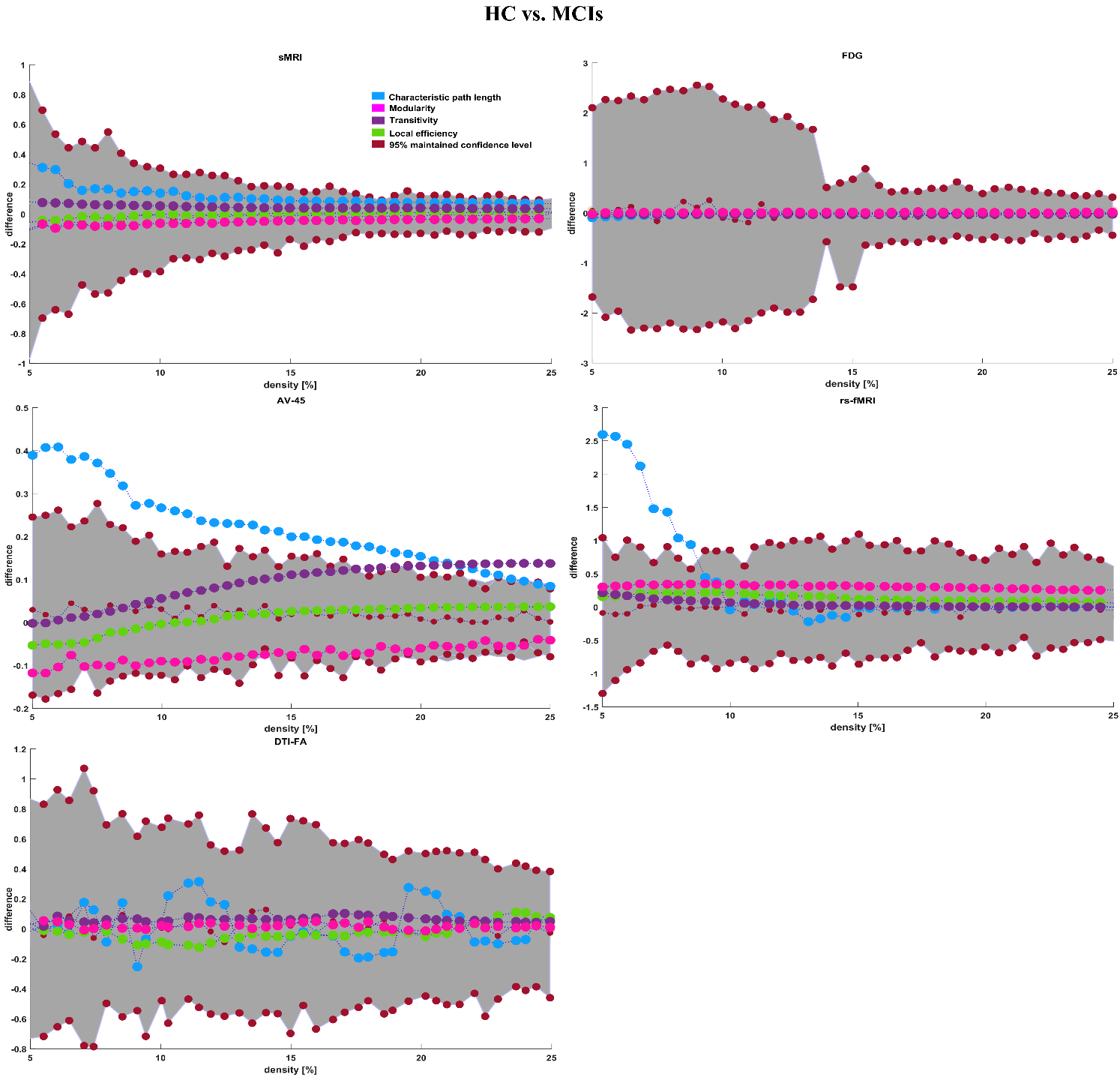


**Figure S5.** Differences between the HC vs. MCIs group in global structural topology. The blue sphere represents characteristics path length, green sphere represents local efficiency, pink sphere represents modularity, purple sphere represents transitivity, and the dark red sphere represents 95% confidence intervals for these measures.


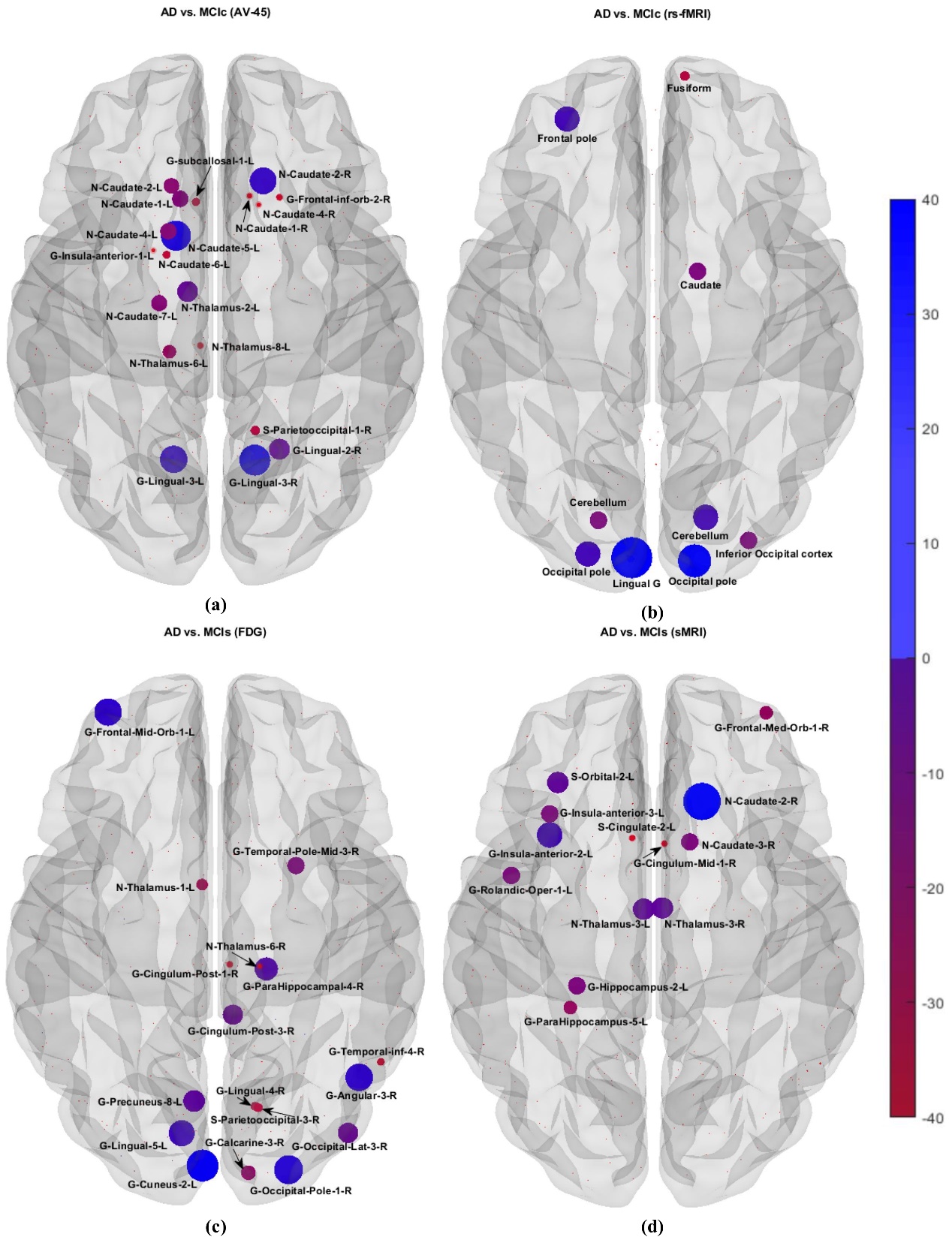


**Figure S6.** Brain maps representing the most predictive regions for distinguish between the AD vs. MCIc and AD vs. MCIs groups. Differences between groups in nodal measures. Nodes showing significant differences among groups in the nodal degree after FDR corrections. For the AD vs. MCIc classification group, AV45 and rs-fMRI show the most significantly affected regions, whereas, for AD vs. MCIs classification group,  FDG-PET and sMRI show the most significantly affected regions. Dark blue shows the most significant region, whereas light red indicates the least significant region.


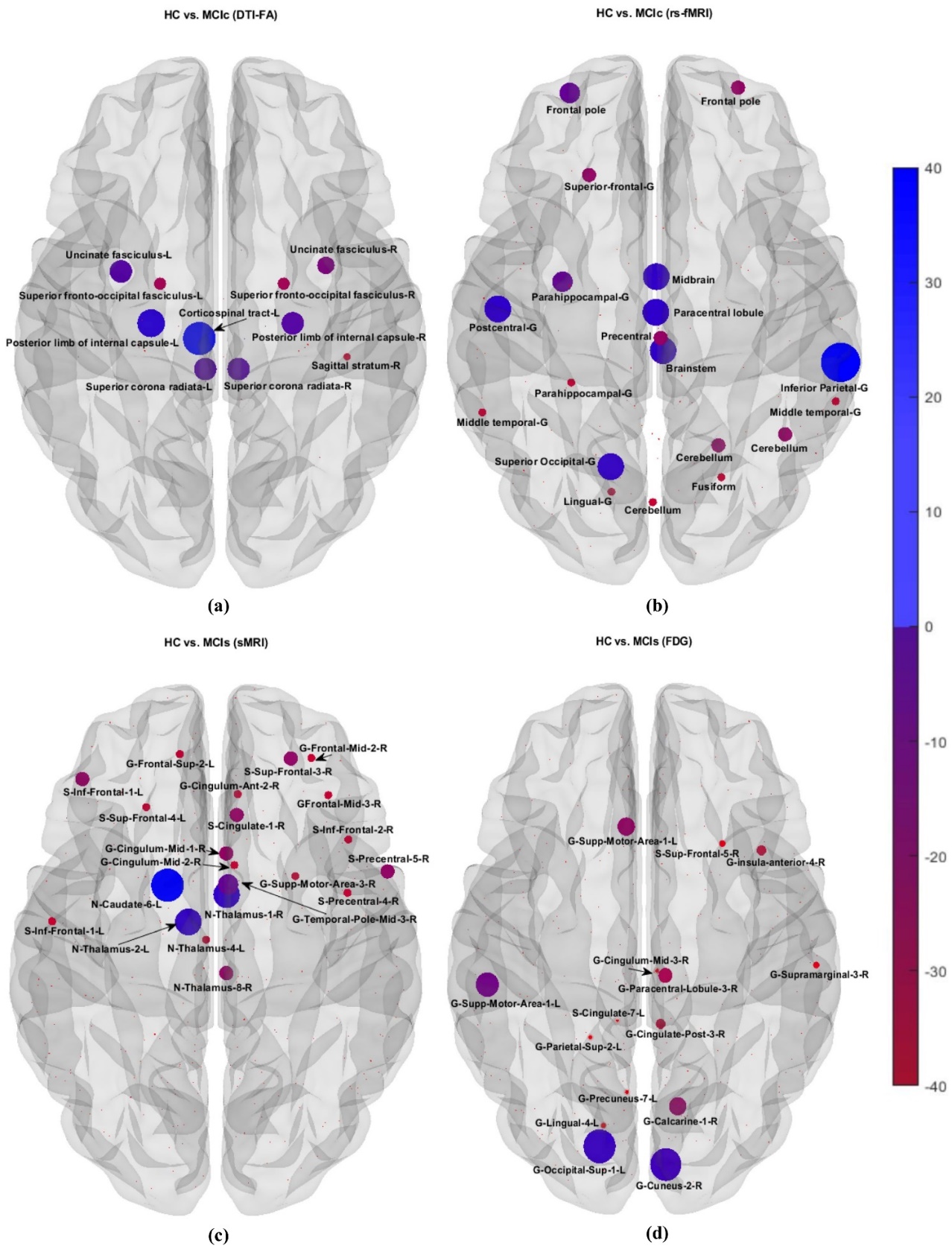


**Figure S7.** Brain maps representing the most predictive regions for distinguish between the HC vs. MCIc and HC vs. MCIs groups. Differences between groups in nodal measures. Nodes showing significant differences among groups in the nodal degree after FDR corrections. For the HC vs. MCIc classification group, DTI-FA and rs-fMRI show the most significantly affected regions, whereas, for HC vs. MCIs classification group,  FDG-PET and sMRI show the most significantly affected regions. Dark blue shows the most significant region, whereas light red indicates the least significant region.
